# IRF2BP2-FOSL2 interaction regulates SOX9-mediated cartilage regeneration in osteoarthritis models

**DOI:** 10.1172/JCI200039

**Published:** 2026-10-15

**Authors:** Hao Zhou, Tiantian Lu, Zhuoqun Gao, Can Cui, Zhiqiang Fang, Yu You, Jing Jiang, Jinjin Xu, Fang Wei, Jing Yu, Hu Zhou, Jinsong Li, Jian Luo, Lei Zhang, Yunyun Jin

**Affiliations:** 1 State Key Laboratory of Cell Biology, Center for Excellence in Molecular Cell Science, Shanghai Institute of Biochemistry and Cell Biology, Chinese Academy of Sciences, University of Chinese Academy of Sciences, Shanghai, China.; 2 Sheng Yushou Center of Cell Biology and Immunology, School of Life Sciences and Biotechnology, Shanghai Jiao Tong University (SJTU), Minhang, Shanghai, China.; 3 Yangzhi Rehabilitation Hospital (Shanghai Sunshine Rehabilitation Centre), Tongji University School of Medicine, Shanghai, China.; 4 Genome Tagging Project (GTP) Center, Shanghai Institute of Biochemistry and Cell Biology, Center for Excellence in Molecular Cell Science, Chinese Academy of Sciences, Shanghai, China.; 5 Department of Analytical Chemistry, State Key Laboratory of Drug Research, Shanghai Institute of Materia Medica, Chinese Academy of Sciences, Shanghai, China.; 6 Key Laboratory of Systems Health Science of Zhejiang Province, School of Life Science, Hangzhou Institute for Advanced Study, University of Chinese Academy of Sciences, Hangzhou, China.; 7 Department of Emergency and Critical Care Medicine, Shanghai Pudong New Area People’s Hospital, Shanghai Jiao Tong University School of Medicine, Shanghai, China.

**Keywords:** Bone biology, Cell biology, Cartilage, Osteoarthritis

## Abstract

Osteoarthritis (OA) is the most common degenerative joint disease. However, the molecular mechanisms underlying OA-induced cartilage degeneration remain elusive. Here, we identify IFN regulatory factor 2 binding protein 2 (IRF2BP2) as an important regulator of chondrocyte differentiation and articular cartilage regeneration. *Irf2bp2* deficiency promoted chondrogenesis and inhibited chondrocyte terminal differentiation by transcriptionally upregulating the key chondrogenic marker SRY-box transcription factor 9 (SOX9), inducing its ectopic expression in the hypertrophic zone. Cartilage-specific KO of *Irf2bp2* elevated SOX9 levels, attenuated OA progression in the destabilization of the medial meniscus (DMM) model, and enhanced cartilage regeneration and hyalinization in the microfracture model. Mechanistically, IRF2BP2 bound to and stabilized Fos-related antigen 2 (FOSL2), an activator protein 1 (AP-1) family member, thereby facilitating FOSL2/c-Jun complex formation and repressing SOX9 expression. Adeno-associated virus–mediated (AAV-mediated) FOSL2 overexpression reversed the protective phenotype observed in *Irf2bp2*-deficient mice. These findings establish the Hippo/IRF2BP2/FOSL2/SOX9 regulatory axis as critical for cartilage regeneration. Importantly, AAV-mediated IRF2BP2 knockdown, pharmacological targeting of the IRF2BP2/AP-1 axis using T-5224, and delivery of a peptide that disrupts IRF2BP2-FOSL2 binding via intra-articular injection all preserved cartilage integrity, offering potential therapeutic strategies for OA and cartilage regeneration.

## Abstract 2

Blocking the IRF2BP2–FOSL2 pathway reduces osteoarthritis progression and enhances cartilage regeneration.

## Graphical abstract

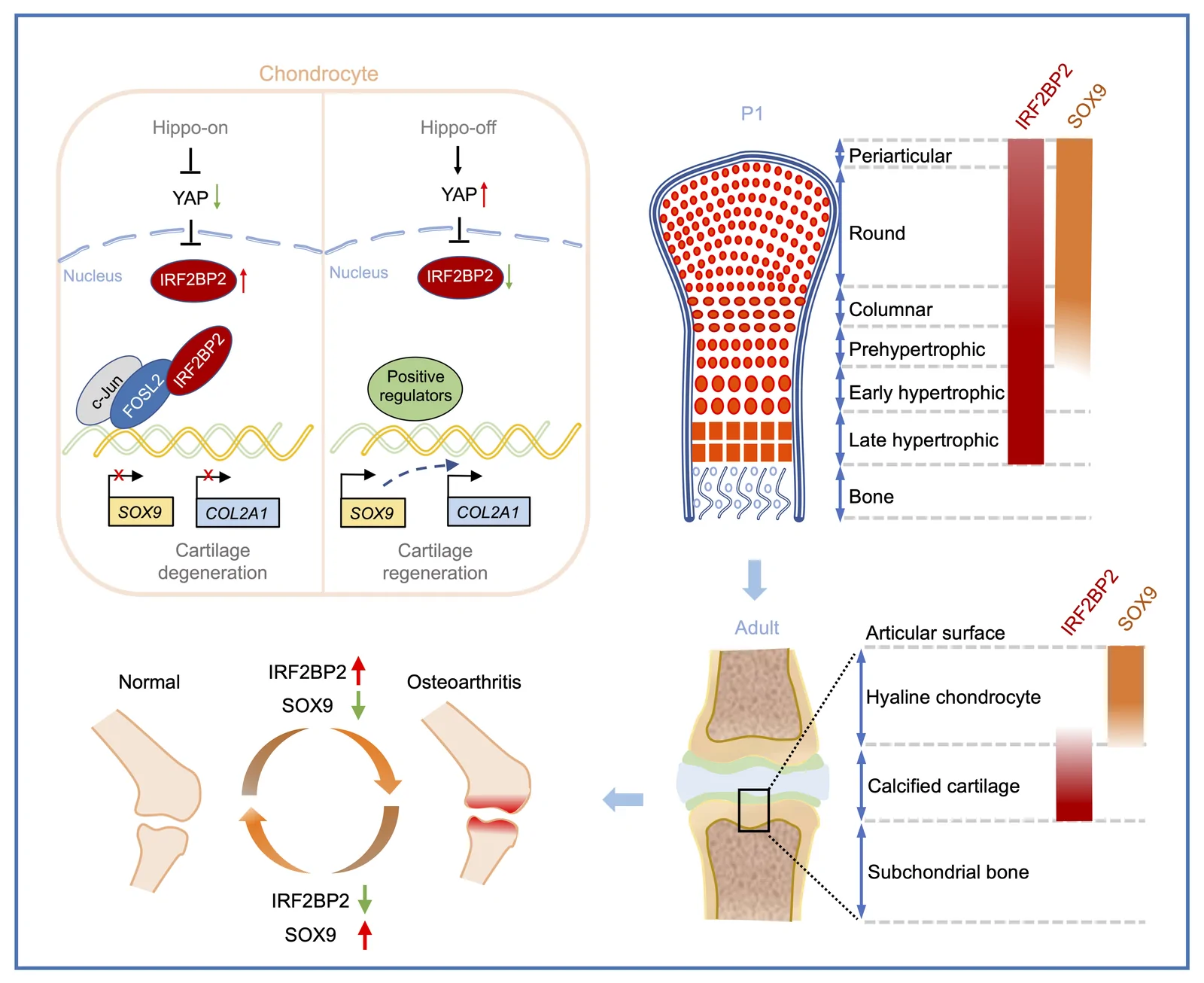

## Introduction

Osteoarthritis (OA) is a complex degenerative joint disease predominantly affecting the elderly population (1). The pathogenesis involves cartilage matrix degradation, synovial hyperplasia, and aberrant subchondral bone remodeling, ultimately leading to osteophyte formation and joint dysfunction (2). The formation of structurally and functionally competent hyaline cartilage at articular defect sites continues to pose fundamental challenges in OA therapeutics (3).

Articular chondrocytes maintain metabolic homeostasis by regulating the dynamic balance of key extracellular matrix (ECM) components, including collagen and proteoglycans (4–6). SRY-box transcription factor 9 (SOX9) is a master regulator of chondrogenesis and the homeostasis of cartilage (7–9). During chondrogenesis, SOX9 is continuously expressed in proliferating and prehypertrophic chondrocytes. Its activity is tightly regulated by various factors in a spatiotemporal manner (10, 11). In the prehypertrophic cartilage zone, the activator protein 1 (AP-1) complex and SOX9 cooperatively bind to the enhancer region of collagen type X alpha 1 chain (COL10A1), regulating chondrocyte hypertrophic differentiation (12, 13). Under pathological conditions, articular cartilage exhibits marked downregulation of SOX9, with reduced expression of type II collagen (COL2A1) and aggrecan (ACAN). Concurrently, matrix metalloproteinases (MMPs) are upregulated, ultimately leading to cartilage breakdown (14). Upregulation of SOX9 in chondrocytes inhibits apoptosis and promotes chondrocyte proliferation and differentiation, thereby attenuating articular cartilage degeneration and protecting articular cartilage from OA.

The Hippo pathway is evolutionarily conserved and is pivotal for regulating organ size and tissue homeostasis (15–18). Its core effector, Yes-associated protein (YAP), orchestrates chondrocyte differentiation during endochondral ossification and cartilage repair. Extensive research has revealed the dual regulatory role of Hippo/YAP signaling in OA pathogenesis. Activated YAP exerts chondroprotective effects by promoting mesenchymal stem cell chondrogenesis, while inhibiting chondrocyte hypertrophy and synovial inflammation. In contrast, YAP inactivation alleviates OA progression through subchondral bone remodeling (19–21). However, the precise molecular mechanisms by which YAP regulates chondrogenesis remain incompletely understood, particularly the identity of the downstream transcriptional effectors that mediate YAP’s functions in chondrocytes.

A recent study has identified IFN regulatory factor 2 binding protein 2 (IRF2BP2) as a transcriptional target of Hippo/YAP signaling in hepatic tissues, where its expression inversely correlates with YAP activity (22), making it a potential downstream candidate for YAP-mediated chondrocyte differentiation. Beyond its role in Hippo signaling, IRF2BP2 acts as a transcriptional regulator that modulates diverse biological processes, including metabolic syndrome, neurological disorders, and tumorigenesis (23–27). However, the role of IRF2BP2 in the skeletal system remains poorly understood, although preliminary evidence suggests its involvement in regulating osteoblast and osteoclast differentiation (28). Elucidating the function and mechanism of IRF2BP2 in chondrogenesis and cartilage regeneration could provide crucial insights into OA pathogenesis.

Here, we demonstrate that cartilage-specific *Irf2bp2* deficiency promoted chondrogenesis and articular cartilage regeneration in OA by upregulating SOX9. We identified Fos-related antigen 2 (FOSL2) as a binding partner of IRF2BP2. IRF2BP2 stabilized FOSL2, facilitating AP-1 complex formation and modulating its transcriptional activity. Furthermore, we found that IRF2BP2 was regulated by the Hippo pathway and served as a hub component in the YAP/SOX9 regulatory axis. Therapeutically, suppression of IRF2BP2 in articular cartilage attenuated OA progression and promoted cartilage regeneration in both destabilization of the medial meniscus (DMM) and microfracture (MF) models. Intra-articular injections of the AP-1 inhibitor T-5224 promoted cartilage repair through pharmacological targeting of the IRF2BP2/AP-1 axis. Notably, a peptide that disrupts the IRF2BP2-FOSL2 interaction effectively attenuated OA progression in vivo, establishing IRF2BP2 as a promising therapeutic target for OA.

## Results

### IRF2BP2 deficiency leads to defective skeletal development.

To investigate the function of IRF2BP2 during skeletal development, we generated *Irf2bp2*-KO mice (Supplemental Figure 1A; supplemental material available online with this article; https://doi.org/10.1172/JCI200039DS1). These neonatal mice died between P1 and P3. *Irf2bp2^–/–^* mice were smaller and lighter than the WT and *Irf2bp2^+/–^* littermate control mice at P1 (Supplemental Figure 1, B and C, and Figure 1, A and B). *Irf2bp2^–/–^* mice showed delayed ossification, characterized by shortened limbs and ribs, bifid xiphoid processes, and a complete absence of ossification in the intermediate phalanges of both the forelimbs and hind limbs. Additionally, fusion of the interfrontal suture was delayed, indicating impaired intramembranous ossification (Figure 1C and Supplemental Figure 1, D and E). Histological analysis revealed marked impairment in bone development, with decreased bone length (Figure 1, D and E, and Supplemental Figure 1F). These phenotypes indicate that IRF2BP2 plays indispensable roles in skeletal development by regulating chondrogenic and osteogenic differentiation.

To examine the role of IRF2BP2 in chondrogenic differentiation, primary articular chondrocytes from WT and *Irf2bp2^–/–^* mice were cultured in chondrogenic medium for 14 and 21 days. Alcian blue staining revealed that *Irf2bp2* deletion markedly enhanced chondrogenic differentiation (Figure 1, F and G). This enhanced chondrogenesis was further confirmed by upregulation of the anabolic genes *Sox9* and *Col2a1* and downregulation of the catabolic genes *Mmp3* and *Mmp13* in *Irf2bp2^–/–^* chondrocytes (Figure 1H).

To investigate the expression pattern of IRF2BP2 during bone development, we generated *HA-Irf2bp2* reporter mice by knocking in an HA tag at the N-terminus of IRF2BP2, thereby reflecting endogenous IRF2BP2 expression (Supplemental Figure 1G). Immunofluorescence (IF) staining revealed distinct spatiotemporal patterns of HA-IRF2BP2 expression. We observed robust expression from the resting zone to the hypertrophic zone at P1 (Figure 1I, upper panels), whereas in the articular cartilage of adult mice (12 weeks), HA signals were mainly detected in hypertrophic chondrocytes (Figure 1I, lower panels). Western blot analysis demonstrated dynamic expression of HA-IRF2BP2 during chondrogenic differentiation, with progressive upregulation during early differentiation (days 0–7) followed by subsequent downregulation in later stages (days 7–14) (Supplemental Figure 1H). Together, our results suggest that *Irf2bp2* KO promotes chondrogenic differentiation and results in abnormal bone development.

### Cartilage-specific deletion of IRF2BP2 induces ectopic expression of SOX9 in hypertrophic chondrocytes and enhances chondrocyte maturation.

SOX9 is expressed in resting, proliferating, and prehypertrophic chondrocytes within the epiphysis and is sharply downregulated during hypertrophy (29). Interestingly, we observed elevated SOX9 and Ki67 expression in the growth plate, along with ectopic expression of both markers within the hypertrophic zone of *Irf2bp2^–/–^* mice at P1 (Figure 1, J and K, and Supplemental Figure 2A). Similarly, we detected elevated SOX9 expression in *Irf2bp2*-KO mice at E14.5 (Supplemental Figure 2, B and C). Primary articular chondrocytes isolated from *Irf2bp2^–/–^* mice exhibited an increased proliferative capacity compared with WT controls (Supplemental Figure 2, D and E).

To further explore the role of IRF2BP2 in cartilage, we generated cartilage-specific *Irf2bp2*-KO mice (*Acan-Cre Irf2bp2^fl/fl^*, hereafter referred to as *Irf2bp2^Acan^*). These mice exhibited progressive skeletal dysplasia from neonatal to adult stages (Figure 1, L and M, and Supplemental Figure 3, A–E). H&E staining of knee joints at 4 and 20 weeks showed increased articular cartilage thickness, with an expanded hyaline cartilage zone, accompanied by a decrease in growth plate thickness (Supplemental Figure 3, F–I). *Irf2bp2^Acan^* mice recapitulated both the enhanced chondrogenesis and ectopic SOX9 expression in hypertrophic chondrocytes observed in *Irf2bp2^–/–^* mice (Figure 1, N and O, and Supplemental Figure 3, J–L). Furthermore, KO of *Irf2bp2* decreased the expression of COL10A1 and inhibited chondrocyte terminal differentiation (Supplemental Figure 3, M and N). Collectively, these findings demonstrate that IRF2BP2 regulated chondrocyte proliferation and differentiation by modulating SOX9 activity and its spatiotemporal expression, thereby ensuring proper chondrocyte maturation.

### Irf2bp2 deletion in cartilage attenuates OA progression and promotes cartilage repair.

Given the established roles of IL-1β and TNF-α in OA pathogenesis (30, 31), we investigated their effects on IRF2BP2 expression in primary articular chondrocytes from *HA-Irf2bp2* mice. Western blot and quantitative reverse transcription PCR (qRT-PCR) analyses revealed that IL-1β or TNF-α treatment upregulated HA-IRF2BP2 and MMP3 expression, while downregulating SOX9 and COL2A1 expression (Figure 2A and Supplemental Figure 4A). Furthermore, this upregulation of IRF2BP2 was recapitulated in vivo, as evidenced by a remarkable increase of IRF2BP2 expression in articular cartilage in the DMM surgery–induced mouse OA model (Figure 2B).

To identify the role of IRF2BP2 in OA, we used 2 independent cartilage-specific *Irf2bp2*-KO mouse lines (*Irf2bp2^Acan^* and *Irf2bp2^Col2-ERT2^*) in the DMM model (Figure 2C). Behavioral pain assessments demonstrated that *Irf2bp2*-deficient mice had higher mechanical pain thresholds in the von Frey assay and prolonged thermal withdrawal latencies in the thermal hyperalgesia test compared with controls 8 weeks after surgery (Figure 2, D and E). Safranin O and Fast Green (SO) staining showed that KO of *Irf2bp2* substantially inhibited cartilage destruction after DMM surgery (Figure 2, F and G). Consistently, Osteoarthritis Research Society International (OARSI) scores were decreased in the *Irf2bp2*-deficient mice after DMM surgery, quantitatively confirming the attenuation of cartilage damage (Figure 2, H and I). This protective effect was further confirmed at the molecular level by IF staining, which demonstrated increased expression of SOX9 and COL2A1, along with decreased MMP3 levels in *Irf2bp2*-deficient cartilage after DMM surgery (Figure 2, J–M, and Supplemental Figure 4, B–D). Notably, meniscal histology scores revealed that *Irf2bp2* deletion also promoted meniscal repair following DMM surgery (Supplemental Figure 4E). Taken together, these results indicate that cartilage-specific deletion of *Irf2bp2* protects articular cartilage from OA and reduces joint pain.

### Loss of Irf2bp2 promotes cartilage regeneration and hyalinization in the MF surgery model.

We next used the MF surgery–induced cartilage defect model to assess the role of IRF2BP2 in cartilage regeneration. *Irf2bp2^Acan^* mice exhibited accelerated cartilage regeneration at both 4 and 8 weeks after MF surgery. As shown in the macroscopic images, the cartilage surface exhibited smoother and more intact filling in *Irf2bp2^Acan^* mice. SO staining analysis demonstrated that *Irf2bp2* KO promoted cartilage regeneration, resulting in higher International Cartilage Repair Society (ICRS) macroscopic scores, as well as stronger proteoglycan staining compared with controls (Figure 3, A–D).

Given that fibrocartilage formation during cartilage repair is inevitable and that fibrocartilage is primarily composed of type I collagen (COL1A1), this compromises functional outcomes through biomechanical inferiority and increased friction by decreasing lubricin secretion (32). Studies have identified that increased SOX9 expression in the defect region promotes cartilage regeneration (33). At 4 weeks after MF, the repair tissue was positive for COL1A1 expression, suggesting an initial fibrocartilaginous phase. By 8 weeks, however, *Irf2bp2^Acan^* mice exhibited fibrocartilage hyalinization, characterized by decreased COL1A1 expression and increased COL2A1 expression (Figure 3, E–H). This enhanced regeneration phenotype was further confirmed in *Irf2bp2^Col2-ERT2^* mice at post-surgery week 8 (Supplemental Figure 5, A–H). Collectively, our findings demonstrate that ablation of *Irf2bp2* enhanced articular cartilage regeneration and hyalinization after MF surgery, which was associated with increased *Sox9* and its downstream target *Col2a1* expression in the cartilage defect zones.

### IRF2BP2 interacts with and stabilizes FOSL2 to promote AP-1–mediated repression of SOX9.

To elucidate the molecular mechanism by which IRF2BP2 modulates chondrocyte proliferation and differentiation, we hypothesized that IRF2BP2 represses SOX9 expression and its downstream transcriptional programs. Gain- and loss-of-function experiments confirmed this hypothesis, as overexpression of IRF2BP2 markedly decreased SOX9 protein levels, whereas IRF2BP2 knockdown promoted SOX9 expression (Figure 4, A and B). Consistently, IRF2BP2 overexpression suppressed the activity of the *Sox9* promoter luciferase reporter (SOX9-Luc) (Figure 4C), indicating that IRF2BP2 regulated SOX9 transcription. Additionally, IRF2BP2 downregulated the 4 × 48-p89-Luc reporter, which contained a reiterated SOX9 binding enhancer sequence coupled to the mouse *Col2a1* minimal promoter (Figure 4D), suggesting that IRF2BP2 also inhibited SOX9-mediated transcriptional regulation.

To identify high-confidence IRF2BP2 interactors, we used proximity-dependent biotin identification (BioID) coupled with mass spectrometry (MS) to characterize the IRF2BP2 interactome. Cells expressing MYC-IRF2BP2-BirA underwent affinity purification followed by liquid chromatography–tandem mass spectrometry (LC-MS/MS) to identify IRF2BP2-interacting proteins (Supplemental Figure 6, A and B). We identified FOSL2 as a putative binding partner of IRF2BP2. Co-IP validated a direct interaction between IRF2BP2 and FOSL2 (Figure 4E).

Previous studies have demonstrated stage-specific enrichment of FOSL2 and c-Jun in prehypertrophic and hypertrophic chondrocytes, where they form functional AP-1 transcription complexes. Direct co-occupancy of SOX9 and AP-1 at shared genomic motifs synergistically regulates the transcription of target genes, such as *Col10a1* and *Col2a1* (12). To determine whether IRF2BP2 functionally cooperates with FOSL2 in AP-1–mediated repression of SOX9 and COL2A1 expression, we performed Western blot analysis. Notably, the expression trends of IRF2BP2, FOSL2, and c-Jun during chondrogenic differentiation were negatively correlated with the levels of COL2A1 and SOX9 (Supplemental Figure 6C). This coexpression pattern is consistent with a potential functional partnership in a shared regulatory pathway. Luciferase assays using SOX9-Luc and 4 × 48-p89-Luc demonstrated that the FOSL2–c-Jun complex suppressed the transcriptional activity of chondrogenic markers *Sox9* and *Col2a1*. Strikingly, overexpression of IRF2BP2 further enhanced AP-1–mediated repression of SOX9 expression, and IRF2BP2 functioned with the AP-1 complex to repress the transcriptional activity of SOX9 (Figure 4F and Supplemental Figure 6D). Co-IP confirmed that IRF2BP2 enhanced the interaction between FOSL2 and c-Jun, thereby promoting AP-1 complex assembly (Figure 4G). ChIP-quantitative PCR (ChIP-qPCR) analysis further showed that IRF2BP2 increased FOSL2 occupancy at the promoter region of *Sox9*, whereas no direct IRF2BP2 binding was detected (Figure 4H and Supplemental Figure 6, E and F). Conversely, ChIP-qPCR analyses indicated AP-1 occupancy at the *Sox9* regulatory region in an IRF2BP2-dependent manner, as AP-1 occupancy was reduced in IRF2BP2-knockdown cells (Supplemental Figure 6G).

Next, we further explored how IRF2BP2 regulates FOSL2 through their interaction. Western blot analysis revealed that IRF2BP2 positively modulated FOSL2 protein levels upon IRF2BP2 overexpression or knockdown. Notably, *Fosl2* mRNA levels remained unchanged, suggesting that the regulatory effect of IRF2BP2 on FOSL2 protein expression was not mediated through transcriptional mechanisms (Figure 4, I and J, and Supplemental Figure 6, H and I). IF staining analysis also confirmed that deletion of *Irf2bp2* reduced the expression of FOSL2 in the hypertrophic zone of P1 femurs (Figure 4, K and L). To assess the protein stability of FOSL2, we then monitored endogenous FOSL2 protein levels after treatment with the protein synthesis inhibitor cycloheximide (CHX). IRF2BP2 overexpression markedly prolonged the half-life of endogenous FOSL2, whereas IRF2BP2 knockdown shortened its half-life (Figure 4, M–P). We next tested whether IRF2BP2 protects FOSL2 from degradation by inhibiting FOSL2 ubiquitination. Our results showed that IRF2BP2, rather than the IRF2BP2-HC2A mutant, dramatically repressed FOSL2 ubiquitination (Supplemental Figure 6J). Collectively, our results demonstrate that IRF2BP2 directly interacted with FOSL2 and stabilized FOSL2 protein levels, thereby enhancing the assembly of the AP-1 complex and modulating its transcriptional repressive activity.

### IRF2BP2 requires its interaction with FOSL2 to suppress SOX9 activity.

To investigate whether IRF2BP2-mediated regulation of SOX9 activity depends on FOSL2, we initially mapped the critical residues governing the IRF2BP2-FOSL2 interaction by generating various truncations encoding different domains of IRF2BP2 and FOSL2. Using alanine-scanning mutagenesis, we identified 2 critical amino acids (H527 and C530) within the RING domain of IRF2BP2 that are crucial for this interaction. Moreover, 3 key amino acids (R99, V102, and I103) within the bZip domain of FOSL2 were determined to be essential for the interaction (Figure 5A).

Mutations in either IRF2BP2 or FOSL2 abolished their interaction (Figure 5B and Supplemental Figure 7A). Consistent with the loss of binding, the IRF2BP2-HC2A mutant failed to stabilize the protein levels of FOSL2 or repress SOX9 expression (Figure 5C). Luciferase reporter assays revealed that the IRF2BP2-HC2A mutant partially impaired the inhibitory function of IRF2BP2 with regard to SOX9 expression and transcriptional activity (Figure 5, D and E). Co-IP analysis confirmed that the IRF2BP2-HC2A mutant failed to promote the interaction between FOSL2 and c-Jun (Supplemental Figure 7B). Additionally, CHX assays confirmed that the IRF2BP2-HC2A mutant did not stabilize the protein levels of FOSL2 (Figure 5, F and G).

Although our data establish that the IRF2BP2-HC2A mutant disrupted the IRF2BP2-FOSL2 interaction and abrogated IRF2BP2’s regulatory effect on FOSL2, previous work indicates that this mutation also impairs IRF2BP2’s interactions with other proteins (22). To definitively test whether IRF2BP2 regulates SOX9 activity exclusively through FOSL2 interaction, we used the FOSL2 mutant (FOSL2-I/A) and FOSL2-deficient cells to assess whether the function of IRF2BP2 was compromised. We found that, while the FOSL2-I/A mutant retained its interaction with c-Jun, IRF2BP2 failed to promote the interaction between FOSL2-I/A and c-Jun (Supplemental Figure 7, C and D). Most conclusively, Western blot analysis demonstrated that IRF2BP2-WT was unable to suppress SOX9 expression in FOSL2-deficient ATDC5 cells, whereas overexpression of FOSL2-WT in IRF2BP2-knockdown cells reversed the regulatory effects of *Irf2bp2* deficiency (Supplemental Figure 7, E and F). Western blot analysis revealed that IL-1β or TNF-α treatment upregulated FOSL2 expression, consistent with the upregulation of IRF2BP2 (Supplemental Figure 7G).

To confirm that IRF2BP2-mediated SOX9 repression depends on FOSL2 in vivo, we used 2 approaches. First, we conducted adeno-associated virus–mediated (AAV-mediated) FOSL2 overexpression in the knee joints of *Irf2bp2^Acan^* mice following DMM surgery. Notably, AAV2/9-FOSL2 injection robustly reversed the protective effects of *Irf2bp2* KO, as evidenced by increased OARSI scores (Figure 5, H and I) and decreased SOX9 and COL2A1 staining (Supplemental Figure 8A). Second, we performed intra-articular injections of 15S-HPETE (a potent AP-1 activator) in cartilage-specific *Irf2bp2*-deficient mice after sham or DMM surgery (34). Activation of the AP-1 complex through 15S-HPETE partially reversed the chondroprotective effects of *Irf2bp2* KO, as evidenced by increased OARSI scores compared with the *Irf2bp2*-KO group (Supplemental Figure 8, B and C). IF staining demonstrated that 15S-HPETE treatment decreased the number of SOX9^+^ cells in *Irf2bp2*-cKO mice after DMM surgery, further confirming that 15S-HPETE attenuated the protective effect of *Irf2bp2* deficiency (Supplemental Figure 8D). Meanwhile, IHC analysis further showed that deletion of *Irf2bp2* reduced FOSL2 and c-Jun expression in hypertrophic chondrocytes (Supplemental Figure 8, E and F). Collectively, these findings indicate that the functional IRF2BP2-FOSL2 interaction is indispensable for IRF2BP2-mediated regulation of SOX9 repression and cartilage regeneration.

### IRF2BP2 represents a promising therapeutic target for OA treatment.

Our study identifies IRF2BP2 as a critical regulator of chondrocyte homeostasis and a potential therapeutic target for OA treatment based on its repression of SOX9. We propose that therapeutic strategies could simultaneously suppress IRF2BP2 activity and enhance endogenous SOX9 function to facilitate articular cartilage regeneration.

To assess the therapeutic potential of IRF2BP2, we performed intra-articular injections of AAV2/9-Cre or AAV2/9-sh-IRF2BP2 into the knee joints of *Irf2bp2^fl/fl^* mice after sham or DMM surgery (Figure 6A). SO staining showed that *Irf2bp2^fl/fl^* mice injected with AAV2/9-Cre or AAV2/9-sh-IRF2BP2 had lower OARSI scores than did control mice, suggesting attenuated cartilage degeneration (Figure 6, B, C, E, and F). IF staining further showed that SOX9 and COL2A1 expression were increased in *Irf2bp2^fl/fl^* mice injected with AAV2/9-Cre or AAV2/9-sh-IRF2BP2, indicating enhanced cartilage matrix synthesis (Figure 6, D and G). Together, these results demonstrate that AAV-mediated *Irf2bp2* knockdown via intra-articular injection promoted cartilage regeneration, highlighting its potential as a therapeutic strategy for cartilage repair. In addition, KO of *Irf2bp2* using adenovirus-mediated Cre recombinase (Ad-iCre) also attenuated cartilage degeneration (Supplemental Figure 9, A–H).

T-5224, a selective AP-1 inhibitor, suppresses the transcriptional activity of FOSL2–c-Jun complexes by blocking their DNA-binding capacity. It has been shown to suppress the expression of MMPs, thereby preventing cartilage destruction in an OA model (35, 36). We found that T-5224 treatment dose-dependently downregulated IRF2BP2, FOSL2, c-Jun, and MMP13 protein levels, but upregulated SOX9 levels (Supplemental Figure 10A). Furthermore, T-5224 attenuated the IL-1β induced upregulation of IRF2BP2 and FOSL2, while enhancing COL2A1 and SOX9 expression (Supplemental Figure 10B). qRT-PCR analysis showed that T-5224 treatment did not change *Irf2bp2* mRNA levels (Supplemental Figure 10C). Ubiquitination assays further showed that T-5224 treatment increased IRF2BP2 ubiquitination (Supplemental Figure 10D). These results suggest that T-5224 regulated IRF2BP2 at the posttranslational level. To examine functional cooperation between IRF2BP2 and the AP-1 complex in cartilage repair, we inhibited AP-1 activity through intra-articular administration of T-5224 in vivo. As expected, combined *Irf2bp2* KO and T-5224 treatment failed to further enhance cartilage repair compared with *Irf2bp2* KO alone (Supplemental Figure 10, E–G). Together with the results that AAV2/9-FOSL2 and the AP-1 activator 15S-HPETE attenuated the chondroprotective effects of *Irf2bp2* KO, these findings indicate that inhibition of the IRF2BP2/AP-1 axis protected cartilage integrity during OA. Thus, targeting the IRF2BP2/AP-1 axis represents a promising therapeutic strategy for OA and warrants further validation.

Moreover, competitive peptide-mediated disruption of the IRF2BP2-FOSL2 interaction represents another promising therapeutic strategy to promote cartilage regeneration. Using IRF2BP2- and FOSL2-truncated constructs (IRF2BP2-526-531 and FOSL2-99-105), we demonstrated effective disruption of the IRF2BP2-FOSL2 interaction (Supplemental Figure 11, A and B). On the basis of our findings, we designed 2 inhibitor peptides targeting the IRF2BP2-FOSL2 interaction for therapeutic purposes. Co-IP showed that both the IRF2BP2-peptide and FOSL2-peptide disrupted the interaction between IRF2BP2 and FOSL2 (Figure 7A). In ATDC5 cells, FOSL2-peptide treatment upregulated SOX9 and COL2A1 expression in a dose-dependent manner (Supplemental Figure 11C). Under IL-1β–induced inflammatory conditions, FOSL2-peptide restored SOX9 expression and suppressed MMP13 protein levels, suggesting a protective effect against inflammation-induced catabolism (Figure 7B). Consistently, intra-articular injections of FOSL2-peptide attenuated cartilage degeneration following DMM surgery, as evidenced by SO staining and decreased OARSI scores (Figure 7, C and D). IF staining further revealed that FOSL2-peptide treatment promoted SOX9 and COL2A1 expression in articular cartilage (Figure 7, E and F, and Supplemental Figure 11, D and E). Cytotoxicity assays showed that neither peptide exhibited toxicity in ATDC5 cells at concentrations up to 40 μg/mL (Supplemental Figure 11F). Uptake studies using FITC-labeled FOSL2-peptide demonstrated efficient cellular entry in vitro and penetration into articular cartilage in vivo (Supplemental Figure 11, G and H). Collectively, these findings provide functional support for further development of peptide-based therapeutics targeting the IRF2BP2-FOSL2 interaction in OA treatment.

### IRF2BP2 responds to Hippo/YAP signaling in chondrocytes and is upregulated in human osteoarthritic cartilage.

IRF2BP2 has been identified as a downstream target of the Hippo pathway (22). To validate whether YAP negatively regulates IRF2BP2 in chondrocytes, we overexpressed YAP or YAP-5SA in ATDC5 cells, which markedly reduced endogenous IRF2BP2 expression (Figure 8A). Luciferase reporter assays further confirmed that YAP and YAP-5SA (a constitutively active mutant) repressed *IRF2BP2* promoter activity, whereas YAP-6SA (a TEA domain transcription factor-binding defective mutant) failed to repress luciferase activity (Figure 8B). Consistently, we found that YAP activation induced by serum deprivation/recovery decreased IRF2BP2 expression and upregulated SOX9 levels in ATDC5 cells (Figure 8C). Conversely, high cell density conditions that inactivate YAP upregulated IRF2BP2 expression in a density-dependent manner (Supplemental Figure 12A). To further validate YAP-mediated regulation of SOX9 activity through IRF2BP2, we generated IRF2BP2-knockdown ATDC5 cells, which almost abolished YAP activation–induced SOX9 and COL2A1 upregulation in the serum recovery assays, suggesting that IRF2BP2 is a hub component of the YAP/SOX9 axis in chondrocytes (Figure 8D).

To establish clinical relevance, we analyzed IRF2BP2 and FOSL2 expression in human OA cartilage samples obtained from 11 individuals undergoing total knee replacement. Western blot and qRT-PCR analyses showed that both IRF2BP2 and FOSL2 were upregulated in damaged regions compared with relatively intact regions of human OA cartilage (Figure 8, E and F). IF staining also revealed that IRF2BP2 expression was increased on the articular cartilage surface of damaged regions, accompanied by reduced SOX9 expression (Figure 8G). Consistently, public RNA-seq data (Gene Expression Omnibus [GEO] accessions GSE243421 and GSE286154) showed that *IRF2BP2* and *FOSL2* were upregulated in human OA samples (Supplemental Figure 12, B and C). Together, our findings suggest that elevated IRF2BP2 and FOSL2 expression is clinically relevant to OA pathogenesis.

## Discussion

This study identifies IRF2BP2 as a transcriptional repressor of SOX9 through interaction with and stabilization of FOSL2, thereby enhancing AP-1–mediated repression of SOX9. We identified the key residues mediating the IRF2BP2-FOSL2 interaction and demonstrated that the regulatory function of IRF2BP2 was largely FOSL2 dependent. Inhibition of IRF2BP2 or disruption of its binding with FOSL2 both enhanced SOX9 activity and promoted cartilage regeneration in vivo. Taken together, these results support a Hippo/IRF2BP2/FOSL2/SOX9 regulatory axis that regulates chondrocyte differentiation and articular cartilage regeneration (Figure 9).

Previous studies have established that the AP-1 signaling pathway regulates cartilage catabolism during OA progression (37–39). Additionally, cooperative binding of SOX9 and AP-1 has been shown to govern the chondrocyte hypertrophic program (12). Several promising AP-1–targeting therapeutic candidates, including T-5224, have been developed for OA treatment (35). Emerging evidence indicates the functional relationship between IRF2BP2 and AP-1 complexes in transcriptional regulation (40, 41). In this study, we identified the interaction between IRF2BP2 and FOSL2 using MS, a finding that expands the role of IRF2BP2 in chondrocytes. Nevertheless, the structural basis of the IRF2BP2-FOSL2 interaction will require future investigation.

Using the DMM and MF models together with OA patient samples, we investigated the role of IRF2BP2 in cartilage repair and regeneration. We observed a correlation between IRF2BP2 expression and OA progression, suggesting that IRF2BP2 may serve as a potential biomarker of OA. The MF model, which creates a full-thickness cartilage defect, not only recapitulates a clinical treatment approach for promoting cartilage regeneration in OA but also simulates the cartilage regeneration process following trauma-induced OA (42, 43). Recent studies have identified the ability of resident skeletal stem cells (SSCs) and chondroprogenitors (CPCs) to regenerate cartilage, with these cells progressively differentiating into aggrecan-expressing chondrocytes (42, 44, 45). In addition, the MF surgical procedure exposes subchondral bone, which may recruit bone marrow–derived progenitors (46, 47). Our findings suggest that downregulation of IRF2BP2 was associated with enhanced cartilage regeneration in the MF model. Although our data support a role for IRF2BP2 during cartilage repair, the precise cell populations mediating these effects remain to be determined. Future lineage-tracing studies will be needed to dissect the contributions of distinct cell populations. We also investigated the role of IRF2BP2 in aging-induced degeneration of articular cartilage. Our results suggest that *Irf2bp2* deficiency may increase articular cartilage thickness in 65-week-old mice (data not shown). Further studies are required to determine if IRF2BP2 is involved in aging-related OA.

OA is a whole-joint disease involving multiple tissues, including the synovium, meniscus, and other joint structures (48, 49). Intra-articular injection of AAVs may affect these tissues and enable sustained transgene expression, supporting its potential for global treatment of knee OA (50–52). In the present study, the protective effects observed after AAV-mediated *Irf2bp2* deletion highlight the therapeutic potential of targeting IRF2BP2 in OA. Because intra-articular AAV delivery may affect multiple joint compartments, further studies should define the tissue-specific contributions to the observed effects.

There were certain limitations in this study. Intra-articular injections are subject to pharmacokinetic constraints, variable bioavailability, and limited tissue penetration (53). Adenoviral vectors can elicit strong immune responses, and repeated injections may cause excessive inflammation (54). Therefore, the protective effects of Ad-mediated IRF2BP2 knockdown should be interpreted with caution. Safety assessment of the FOSL2-peptide remains preliminary, and long-term safety studies are required. ICRS scoring was performed on a slide that contained 4 standardized sections corresponding to the largest defect area in the MF model to ensure consistent evaluation across samples, which may not have fully captured the heterogeneity of cartilage repair. Further studies using multiple serial sections will provide a more comprehensive evaluation. In addition, we only detected a correlation between IRF2BP2 expression and OA in human samples. Additional studies are needed to determine the role of IRF2BP2 in patients with OA. Finally, the development of selective IRF2BP2 inhibitors represents a key therapeutic strategy for OA, with considerable translational potential.

Extensive studies have indicated that exogenous SOX9 expression markedly alleviates cartilage degeneration and reduces OA-associated inflammatory cytokines (33, 55). However, direct delivery of SOX9 remains challenging because current gene delivery approaches are limited by safety and transduction efficiency. Moreover, uncontrolled or excessive SOX9 activation may lead to undesirable effects (56–58). Therefore, identifying and targeting the upstream regulatory network governing cartilage-intrinsic SOX9 expression represents a safer and more controllable therapeutic strategy. Here, we identify a Hippo/IRF2BP2/FOSL2/SOX9 regulatory axis and propose 4 therapeutic strategies for cartilage repair: (a) genetic ablation of *Irf2bp2*; (b) pharmacological targeting of the IRF2BP2/AP-1 axis, such as with T-5224; (c) targeted disruption of the IRF2BP2-FOSL2 protein-protein interaction through engineered competitive peptides; and (d) upstream pathway modulation via YAP activation to relieve IRF2BP2-mediated SOX9 repression. We propose that these strategies hold substantial therapeutic potential for treating cartilage defects and promoting cartilage regeneration in OA. Our findings provide preclinical evidence supporting the translational potential of these strategies for clinical applications.

## Methods

### Sex as a biological variable.

Sex was not systematically analyzed as a biological variable in the human cartilage samples. In animal experiments, only male mice were used because male mice have been reported to develop more severe and consistent OA phenotypes than female mice in surgically induced OA models (59). Thus, the in vivo findings should be interpreted in the context of male mice, and future studies will be needed to determine whether these findings extend to female mice.

### Collection and preparation of human articular cartilage.

Human articular cartilage samples were collected from patients with OA undergoing total knee replacement surgery. The full-thickness cartilage tissues (approximately 3 × 3 mm) were harvested from the tibial plateau, avoiding subchondral bone. Cartilage tissues classified as relatively intact (from non-weight-bearing areas) and those classified as damaged (from weight-bearing lesioned areas) were collected from patients and analyzed using unpaired statistical analysis for qRT-PCR data. After collection, cartilage samples were immediately snap-frozen in liquid nitrogen and pulverized for subsequent RNA or protein extraction, or fixed for histological analysis. For histological evaluation, cartilage tissues were embedded and sectioned along the coronal plane. All samples were collected with informed consent from the patients and immediately processed for further analysis or stored at –80°C until use.

### Mouse lines.

All mice were kept in a specific pathogen-free (SPF) environment at SJTU with protocols approved by Laboratory Animal Center. All genetically modified mice were backcrossed onto the C57BL/6J background for more than 10 generations.

*Irf2bp2*-KO (*Irf2bp2^–/–^*), *Irf2bp2*-floxed (*Irf2bp2^fl/fl^*), and *HA-Irf2bp2*-knockin mice were generated using CRISPR/Cas9 technology. Cartilage-specific *Irf2bp2*-KO mice were generated by crossing *Irf2bp2^fl/fl^* mice with *Aggrecan-Cre* (*Acan-Cre*) mice to generate *Acan-Cre Irf2bp2^fl/fl^* (*Irf2bp2^Acan^*) mice, or with *Col2-CreERT2* mice to generate tamoxifen-inducible *Col2-CreERT2 Irf2bp2^fl/fl^* (*Irf2bp2^Col2-ERT2^*) mice. Detailed information on mouse generation, including sgRNA sequences, targeting strategies, and genotyping protocols, is provided in the Supplemental Methods.

### Mouse model.

For the DMM surgery–induced OA mouse model, 12-week-old male mice were anesthetized by i.p. injection of avertin (250 mg/kg). The right knee joint was exposed through a midline skin incision, and the patella was laterally dislocated with forceps to access the medial meniscotibial ligament (MMTL). The MMTL was transected using microsurgical scissors, resulting in destabilization of the medial meniscus. Sham-operated control mice underwent identical procedures except for MMTL transection.

For the MF surgery–induced cartilage defect mouse model, 12-week-old male littermates were used to evaluate articular cartilage regeneration following MF surgery. Under general anesthesia with avertin, a medial parapatellar incision was made to expose the femoral condyles. A customized 26 gauge needle (sheathed to limit penetration depth to 400 μm below the needle tip) was used to create full-thickness articular cartilage defects. The full-thickness articular cartilage defects were created in the right femoral patella groove, which is anatomically located in the anterior compartment of the knee joint. The central weight-bearing area of the trochlea was identified under direct visualization via an anteromedial arthrotomy. The left femur was sham-operated, in which the patella was exposed without damaging the cartilage. The patella was repositioned, and joint capsule and skin were sutured separately. Four and 8 weeks after sham or MF surgery, the repaired articular cartilage was histologically analyzed and scored. For histological analysis, serial sections (10 μm thickness) were cut coronally through the entire defect region. The defect boundaries were monitored continuously from the first appearance of the defect until its disappearance. The section displaying the maximum defect area was selected as the representative section for each sample. The defect region was defined as the area between the 2 intact cartilage margins flanking the defect. The defect was identified by (a) disruption of the articular surface, (b) loss of safranin O staining, and (c) the presence of fibrocartilage tissue. For each sample, the region of interest (ROI) was delineated on the representative section showing the maximum defect area, and this section was used for subsequent quantification and ICRS scoring. Adjacent sections were used for immunofluorescence staining and other experiments. Cartilage repair was evaluated using the ICRS macroscopic or histological scoring system (60). Two independent observers, blinded to genotype and treatment, scored each sample.

For intra-articular injections of adenovirus or drugs, mice were prepared for surgery as described above. Mice were anesthetized and placed in a supine position with the target limb extended. A small skin incision was made to expose the patella and tendon. A 10 μL microsyringe was inserted through the tendon and into the space between the patella and the femur, and a total volume of 6 μL was injected. Detailed information on injection concentrations and frequencies is provided in the Supplemental Methods.

### Mechanical threshold sensory test.

Mechanical withdrawal thresholds were assessed using an electronic von Frey device (BIO-EVF3). Mice were placed in individual enclosures on a wire mesh platform and allowed to acclimate for 30 minutes. A rigid probe was applied perpendicularly to the mid-plantar surface of the right hind paw with gradually increasing force. The threshold was defined as the force at which the mouse withdrew its paw, automatically recorded by the device. All measurements were performed by a single experimenter blinded to genotype and treatment. Data are presented as absolute thresholds, and all comparisons are within the same experimental cohort under identical testing conditions.

### Design of IRF2BP2 and FOSL2 peptides.

The amino acid sequence of the IRF2BP2 peptide was as follows: GRKKRRQRRRPPQGGGGSGGGGSPKKKRKVSHKFCF. The amino acid sequence of the FOSL2 peptide was as follows: GRKKRRQRRRPPQGGGGSGGGGSPKKKRKVRPGVIKT. Both peptides were synthesized by GenScript Biotech.

### Cell culture.

The ATDC5 murine chondrogenic cell line was provided in-house. Cells were cultured in a 1:1 mixture of DMEM/F12 medium (Meilunbio, MA0214-2) supplemented with 10% FBS (Excell Bio, 021C0420A) and 1% penicillin-streptomycin solution (Meilunbio, MA0110-1). The human chondrosarcoma cell line SW1353 was purchased from the Cell Bank of the Chinese Academy of Sciences. Cells were cultured in DMEM medium (Meilunbio, MA0212-2) supplemented with 10% FBS and 1% penicillin-streptomycin solution. Both cell lines were maintained at 37°C in a humidified atmosphere containing 5% CO_2_. The HEK-293T cell line was cultured as previously described (22) and was obtained from the American Type Culture Collection (ATCC).

### Luciferase reporter assay.

The SOX9-Luc and 4 × 48-p89-Luc reporter plasmids were constructed as previously described (61). The *IRF2BP2* promoter luciferase reporter construct contained approximately 2 kb of the human *IRF2BP2* promoter region upstream of the transcription start site. Luciferase plasmids, other indicated plasmids, and the CMV-*Renilla* plasmid were transfected into ATDC5 cells. After 36 hours, luciferase activity was measured following the standard protocol of the Dual-Luciferase Assay kit (Promega, E1960) on a GloMax 20/20 luminometer (Promega).

### Statistics.

Comparisons between 2 groups were performed using a 2-tailed, unpaired Student’s *t* test in GraphPad Prism 10 (GraphPad Software). Comparisons among more than 2 groups were performed using 1-or 2-way ANOVA, followed by Tukey’s multiple-comparison test. The results are shown as the mean ± SD.

### Study approval.

All animal experiments were conducted in accordance with the guidelines of the Laboratory Animal Center of SJTU and were approved by the university’s IACUC (approval no. A2023063-003).

For human studies, articular cartilage samples were obtained from patients with OA undergoing total knee arthroplasty at Shanghai Sixth People’s Hospital. This study was approved by the Human Ethics Committee of Shanghai Sixth People’s Hospital (2021-048). Written informed consent was obtained from all patients prior to sample collection.

### Data availability.

The authors confirm that the data supporting the findings of this study are available within the article and its supplemental materials, including the Supporting Data Values file. Additional experimental procedures are described in the supplemental material.

## Author contributions

Hao Zhou conducted most of the experiments, analyzed the data, and wrote the manuscript. TL maintained the mice and screened the IRF2BP2-interacting proteins. ZG performed the chondrogenic differentiation experiments. CC and ZF assisted with the experiments and analyzed the data. J Luo provided the human samples, and YY helped with these experiments. J Li, Hu Zhou, JY, FW, JX, and JJ provided reagents and comments. YJ and LZ conceived and supervised the study, revised the manuscript, and provided funding.

## Conflict of interest

LZ and YJ hold the patent no. 202610155105.7, titled “Use of IRF2BP2 as a target in the preparation of drugs for regulating chondrocyte differentiation and promoting articular cartilage regeneration or repair.”

## Funding support

- National Natural Science Foundation of China (grant nos. 32293233, 32221002, and 32030025, to LZ; grant no. 32370751, to YJ).
- Shanghai Municipal Science and Technology Major Project (to LZ).
- Shanghai Leading Talents Program (to LZ).

## Supplementary Material

Supplemental data

Unedited blot and gel images

Supporting data values

## Figures and Tables

**Figure 1:**
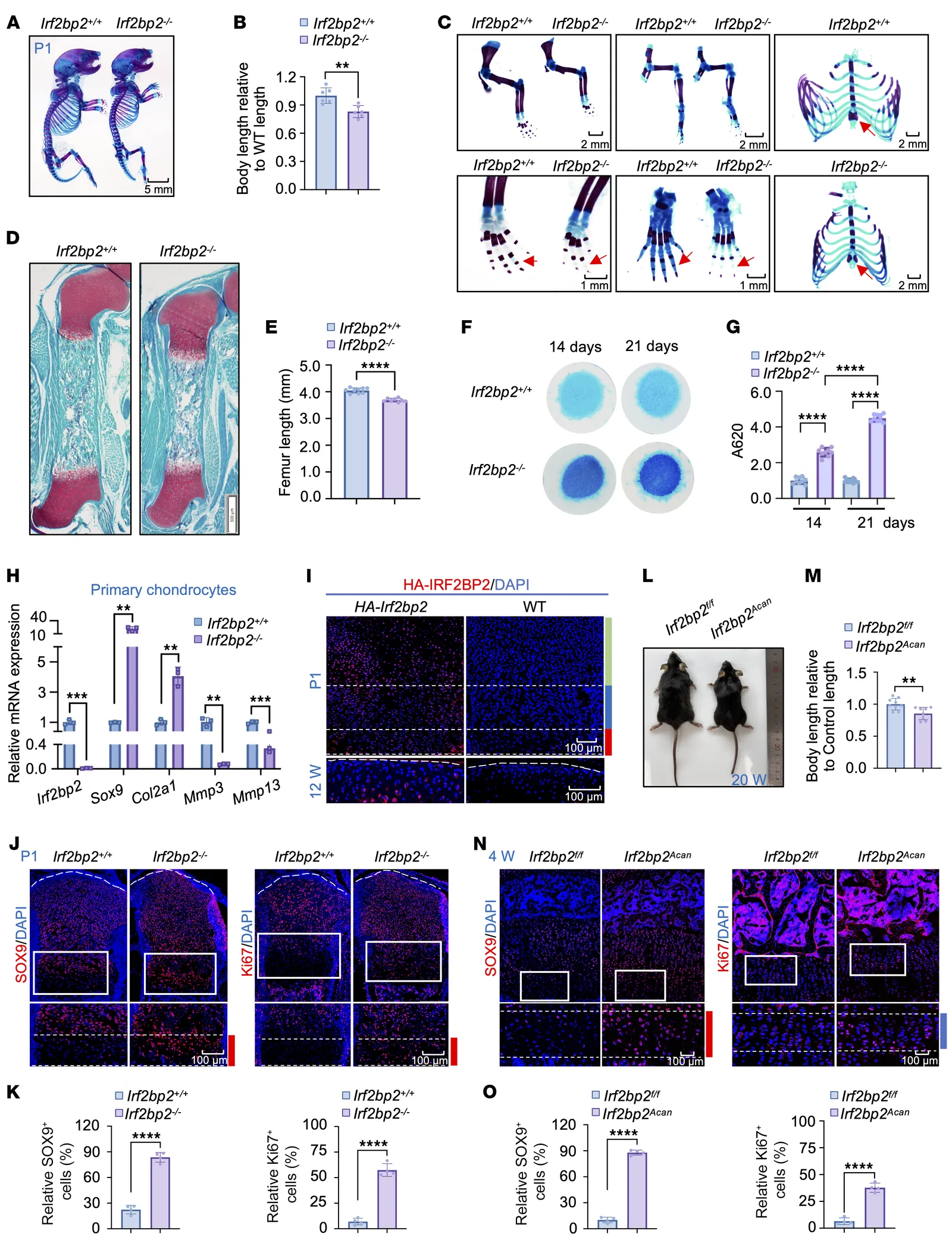
*Irf2bp2* deficiency leads to abnormal bone development and promotes SOX9 expression in cartilage. (**A**) Whole-mount skeletal staining with alizarin red and Alcian blue of WT and *Irf2bp2^–/–^* mice at P1. Scale bar: 5 mm. (**B**) Quantification of body length in **A** (*n* = 6). (**C**) Limb and sternum staining at P1. Scale bars: 1 mm and 2 mm. (**D**) SO staining of femurs at P1. Scale bar: 500 μm. (**E**) Quantification of the femur lengths in **D** (*n* = 8). (**F**) Alcian blue staining of primary articular chondrocytes from WT and *Irf2bp2^–/–^* newborn mice after 14 or 21 days of chondrogenic culturing. (**G**) Quantification of the Alcian blue staining in **F** (*n* = 9). (**H**) qRT-PCR analysis of *Irf2bp2*, *Sox9*, *Col2a1*, *Mmp3*, and *Mmp13* mRNA levels in primary articular chondrocytes from WT and *Irf2bp2^–/–^* mice. (**I**) IF staining for HA in knee joints from WT and *HA-Irf2bp2* mice at P1 and 12 weeks. Scale bars: 100 μm. (**J**) IF staining for SOX9 and Ki67 in WT and *Irf2bp2^–/–^* mice at P1. Scale bars: 100 μm. Original magnification, ×10. (**K**) Quantification of SOX9 and Ki67 in **J** (*n* = 4). (**L**) Representative view of 20-week-old *Irf2bp2^fl/fl^* and *Irf2bp2^Acan^* mice. (**M**) Quantification of body lengths in **L** (*n* = 8). (**N**) IF staining for SOX9 and Ki67 in *Irf2bp2^fl/fl^* and *Irf2bp2^Acan^* mice at 4 weeks. Scale bars: 100 μm. Original magnification, ×10. (**O**) Quantification of SOX9 and Ki67 in **N** (*n* = 4). In **B**, **E**, **G**, **H**, **K**, **M**, and **O**, data are shown as the mean ± SD. *P < 0.05, **P < 0.01, ***P < 0.001, and ****P < 0.0001, by 2-tailed, unpaired Student’s *t* test (**B**, **E**, **H**, **K**, **M**, and **O**) and 1-way ANOVA, followed by Tukey’s multiple-comparison test (**G**). In **I** and **J**, dotted and curved lines indicate the surface of articular cartilage. Color bars denote skeletal zones: red indicates hypertrophic chondrocytes; blue indicates proliferative and prehypertrophic chondrocytes; and green indicates resting chondrocytes.

**Figure 2:**
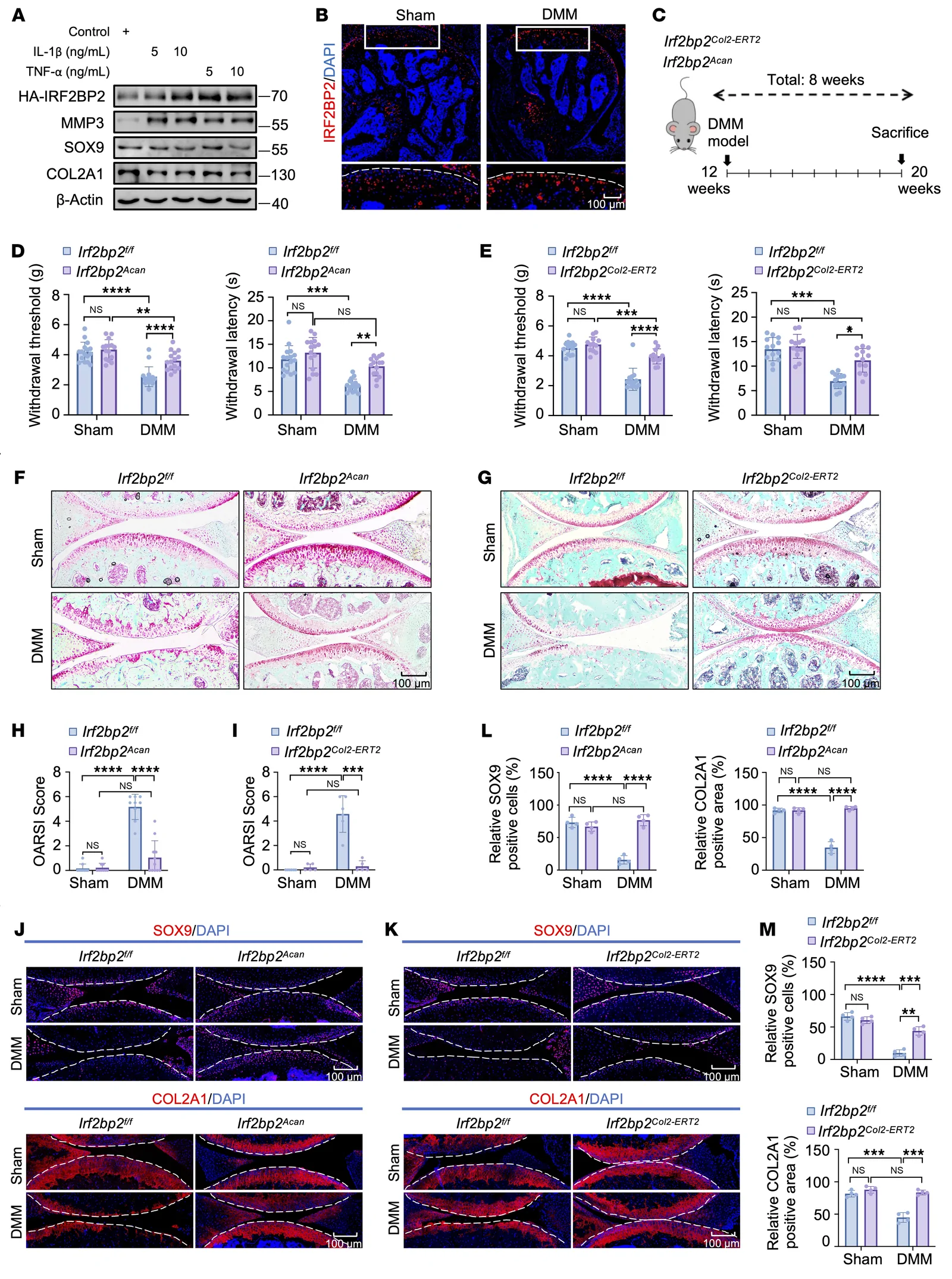
Cartilage-specific deletion of *Irf2bp2* attenuates OA progression and associated pain. (**A**) Western blot analysis of HA-IRF2BP2 expression in primary articular chondrocytes isolated from *HA-Irf2bp2* mice at P1 following a 24-hour treatment with IL-1β and TNF-α. Data are representative of 3 independent biological replicates. (**B**) IF staining analysis of IRF2BP2 expression in 12-week-old WT mice 8 weeks after sham or DMM surgery. Scale bar: 100 μm. Original magnification, ×4. (**C**) Schematic outline of sham and DMM surgery after deletion of *Irf2bp2* in male *Irf2bp2^Acan^* and *Irf2bp2^Col2-ERT2^* mice. (**D** and **E**) Pain was measured by von Frey assay and a radiant heat paw-withdrawal test in *Irf2bp2^fl/fl^* and *Irf2bp2^Acan^* mice (*n* = 15) (**D**) or *Irf2bp2^fl/fl^* and *Irf2bp2^Col2-ERT2^* mice (*n* = 13) (**E**). (**F** and **G**) SO staining of knee joint cartilage in *Irf2bp2^fl/fl^* and *Irf2bp2^Acan^* mice (**F**) or *Irf2bp2^fl/fl^* and *Irf2bp2^Col2-ERT2^* mice (**G**) after sham or DMM surgery. Scale bars: 100 μm. (**H** and **I**) OARSI scores for samples in **F** (*n* = 9) and (**G**) (*n* = 5). (**J** and **K**) IF staining for SOX9 and COL2A1 in *Irf2bp2^fl/fl^* and *Irf2bp2^Acan^* mice (**J**) or *Irf2bp2^fl/fl^* and *Irf2bp2^Col2-ERT2^* mice (**K**) after sham or DMM surgery. Scale bars: 100 μm. Original magnification, ×10. (**L** and **M**) Quantification of the expression of SOX9 and COL2A1 in **J** and **K** (*n* = 4). Data are shown as the mean ± SD. *P < 0.05, **P < 0.01, ***P < 0.001, and ****P < 0.0001, by 2-way ANOVA, followed by Tukey’s multiple-comparison test (**D**, **E**, **H**, **I**, **L**, and **M**). In **B**, **J**, and **K**, dotted and curved lines indicate the surface of articular cartilage.

**Figure 3:**
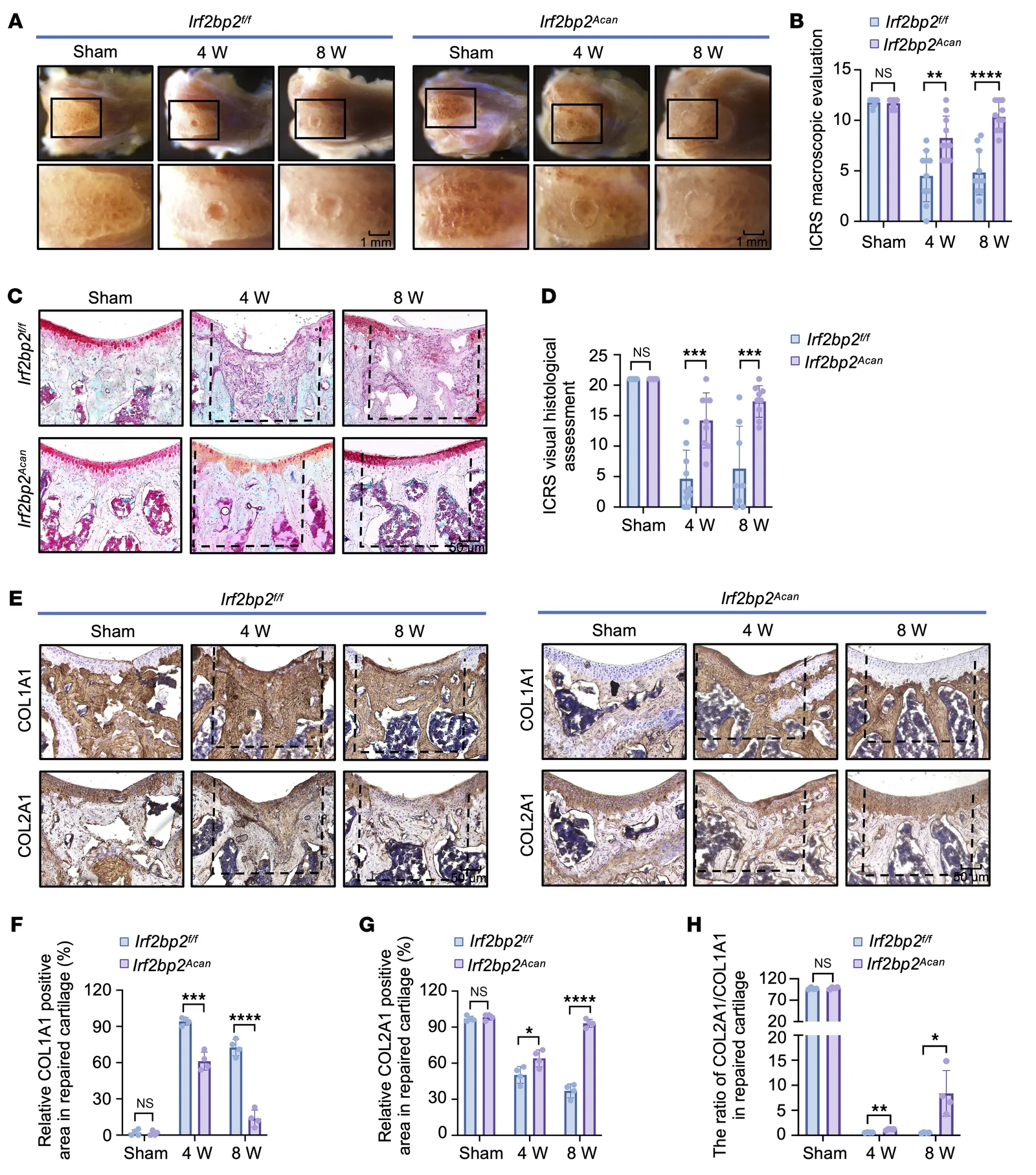
Cartilage-specific loss of *Irf2bp2* inhibits fibrocartilage formation, facilitating cartilage regeneration and hyalinization. (**A**) Representative images of the distal femur from sham- or MF-operated *Irf2bp2^fl/fl^* and *Irf2bp2^Acan^* mice, 4 and 8 weeks after surgery. Scale bars: 1 mm. (**B**) ICRS macroscopic evaluation score assessment for the images shown in **A** (*n* = 7). (**C**) SO staining of the articular cartilage defect sections. Scale bar: 50 μm. (**D**) ICRS visual histological score of the regenerated tissues in **C** (*n* = 8). (**E**) Representative images of IHC staining for COL1A1 and COL2A1 in articular cartilage defect sections. Scale bars: 50 μm. (**F** and **G**) Quantification of the expression of COL1A1 (**F**) and COL2A1 (**G**) in the articular cartilage defect sections shown in **E** (*n* = 4). (**H**) Quantification of the COL2A1/COL1A1 intensity ratio in the repaired cartilage in **E** (*n* = 4). In **B**, **D**, and **F**–**H**, data are shown as the mean ± SD. *P < 0.05, **P < 0.01, ***P < 0.001, and ****P < 0.0001, by 2-way ANOVA, followed by Tukey’s multiple-comparison test (**B**, **D**, and **F**–**H**). In **C** and **E**, the ROIs are indicated by black dotted lines.

**Figure 4:**
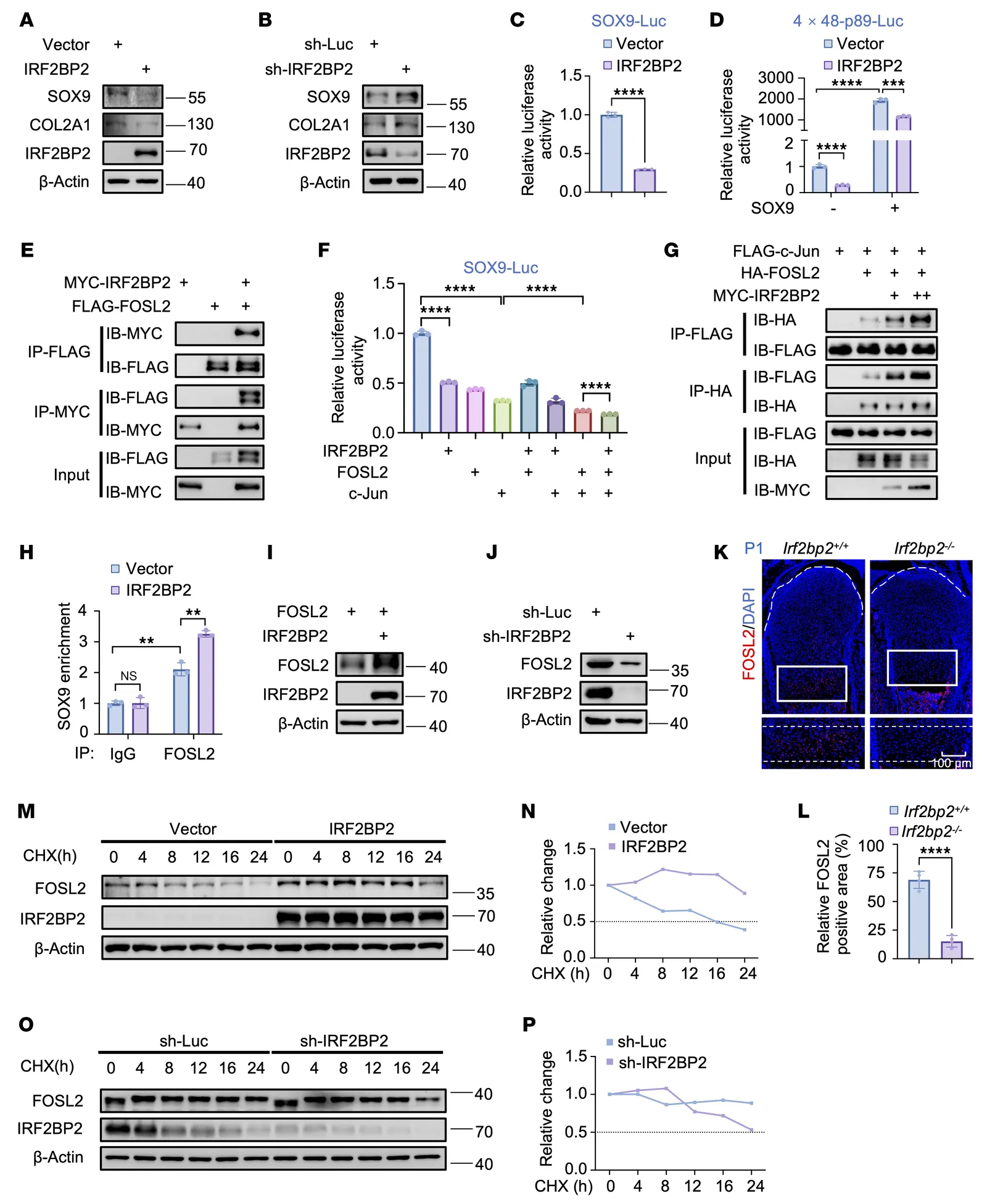
IRF2BP2 interacts with and stabilizes FOSL2 to inhibit SOX9 transcriptional activation. (**A** and **B**) Western blot analysis of SOX9 and COL2A1 in ATDC5 cells after IRF2BP2 overexpression (**A**) or knockdown (**B**). (**C**) *Sox9* promoter–luciferase reporter in ATDC5 cells. (**D**) 4 × 48-p89-Luc activity in ATDC5 cells cotransfected with SOX9 and IRF2BP2. (**E**) A co-IP experiment of IRF2BP2 and FOSL2 was performed using HEK-293T cells. (**F**) Effects of IRF2BP2, FOSL2, and c-Jun on the *Sox9* promoter reporter in ATDC5 cells. (**G**) Co-IP analysis of the effect of IRF2BP2 on the interaction between c-Jun and FOSL2. (**H**) ChIP-qPCR analysis of the effect of IRF2BP2 on FOSL2 binding to the promoter region of *SOX9* in SW1353 cells. (**I** and **J**) Protein levels of FOSL2 were determined in ATDC5 cells after IRF2BP2 overexpression (**I**) or knockdown (**J**). (**K**) IF staining for FOSL2 in WT and *Irf2bp2^–/–^* mice at P1. Scale bar: 100 μm. (**L**) Quantification of FOSL2 staining in **K** (*n* = 4). (**M**) ATDC5 cells were transfected with vector or IRF2BP2, treated with CHX, and harvested at the indicated time points. The protein levels of endogenous FOSL2 were determined. (**N**) Quantitative analysis of the FOSL2 panels in **M** using ImageJ. (**O**) Control (sh-Luc) and IRF2BP2-knockdown ATDC5 cells were treated with CHX and harvested at the indicated time points. The protein levels of FOSL2 were determined. (**P**) Quantitative analysis of FOSL2 panels in **O** using ImageJ. In **C**, **D**, **F**, **H**, and **L**, data are shown as the mean ± SD. **P < 0.01, ***P < 0.001, and ****P < 0.0001, by 2-tailed, unpaired Student’s *t* test (**C** and **L**), 1-way ANOVA, followed by Tukey’s multiple-comparison test (**F**), and 2-way ANOVA, followed by Tukey’s multiple-comparison test (**D** and **H**). Dotted lines in **K** indicate the surface of articular cartilage. IB, immunoblot.

**Figure 5:**
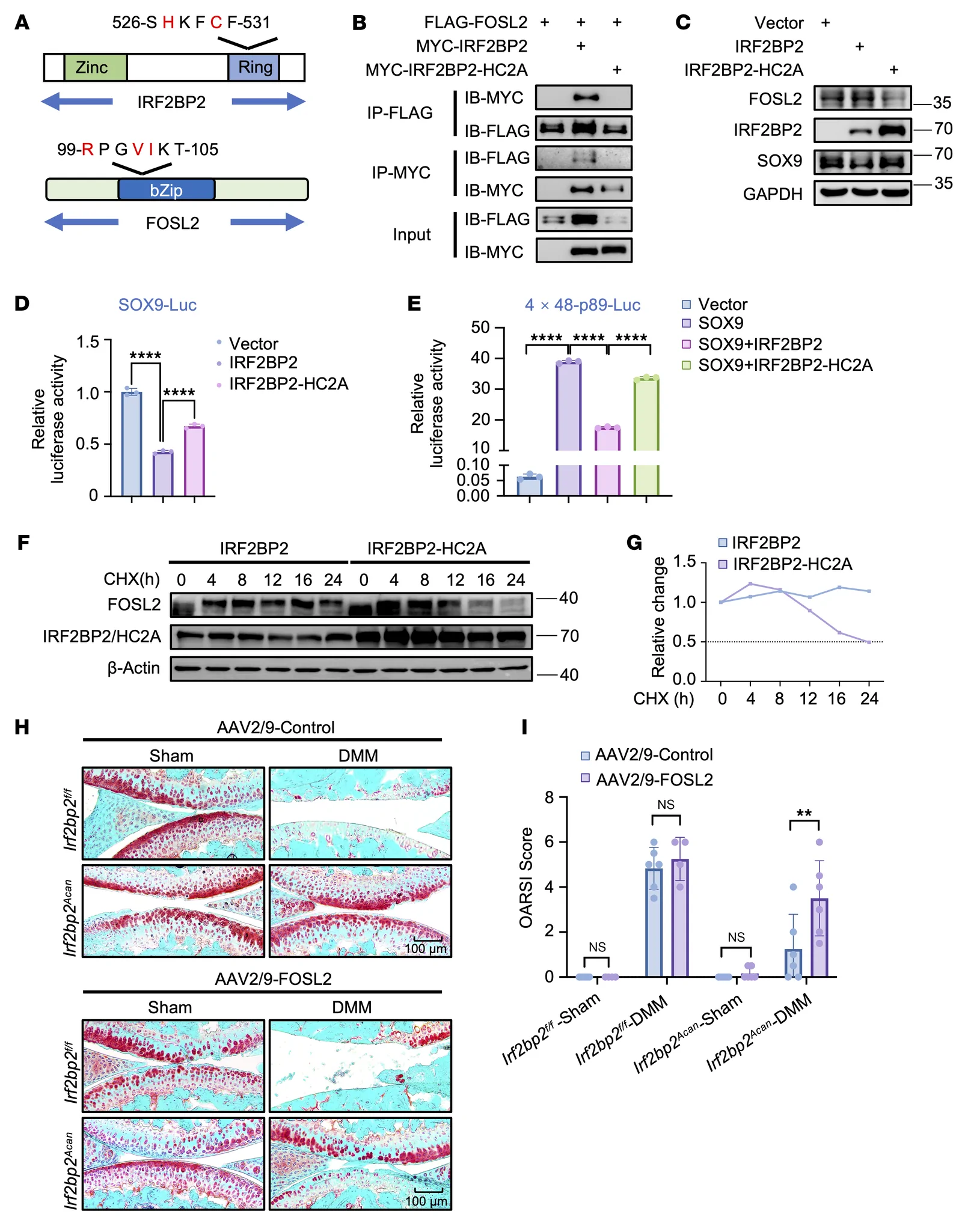
IRF2BP2 requires FOSL2 interaction to inhibit SOX9 activity. (**A**) Schematic illustration of the domain organization for human IRF2BP2 and FOSL2, identifying the crucial interaction residues. (**B**) Co-IP assays of IRF2BP2/IRF2BP2-HC2A and FOSL2 were performed in HEK-293T cells. (**C**) Protein levels of FOSL2 were determined by Western blotting in ATDC5 cells overexpressing IRF2BP2 or IRF2BP2-HC2A. (**D** and **E**) *Sox9* promoter-luciferase (**D**) and 4 × 48-p89-Luc (**E**) activity was measured in ATDC5 cells. (**F**) ATDC5 cells were transfected with IRF2BP2 or IRF2BP2-HC2A, then CHX was added and the cells were harvested at the indicated time points. The protein levels of endogenous FOSL2 were determined by Western blotting. (**G**) Quantitative analysis of FOSL2 panels in **F** using ImageJ. (**H**) SO staining of cartilage of the knee joints in *Irf2bp2^fl/fl^* and *Irf2bp2^Acan^* mice treated with AAV2/9-control or AAV2/9-FOSL2 (1 × 10^13^ vg/mL) after sham or DMM surgery. (**I**) OARSI scores for samples in **H** (*n* = 6 mice per group, except for *Irf2bp2^fl/fl^*-sham-AAV-FOSL2 and *Irf2bp2^fl/fl^*-DMM-AAV-FOSL2 groups, which included 4). In **D**, **E**, and **I**, data are shown as the mean ± SD. **P < 0.01 and ****P < 0.0001, by 1-way ANOVA, followed by Tukey’s multiple-comparison test (**D** and **E**) and 2-way ANOVA, followed by Tukey’s multiple-comparison test (**I**).

**Figure 6:**
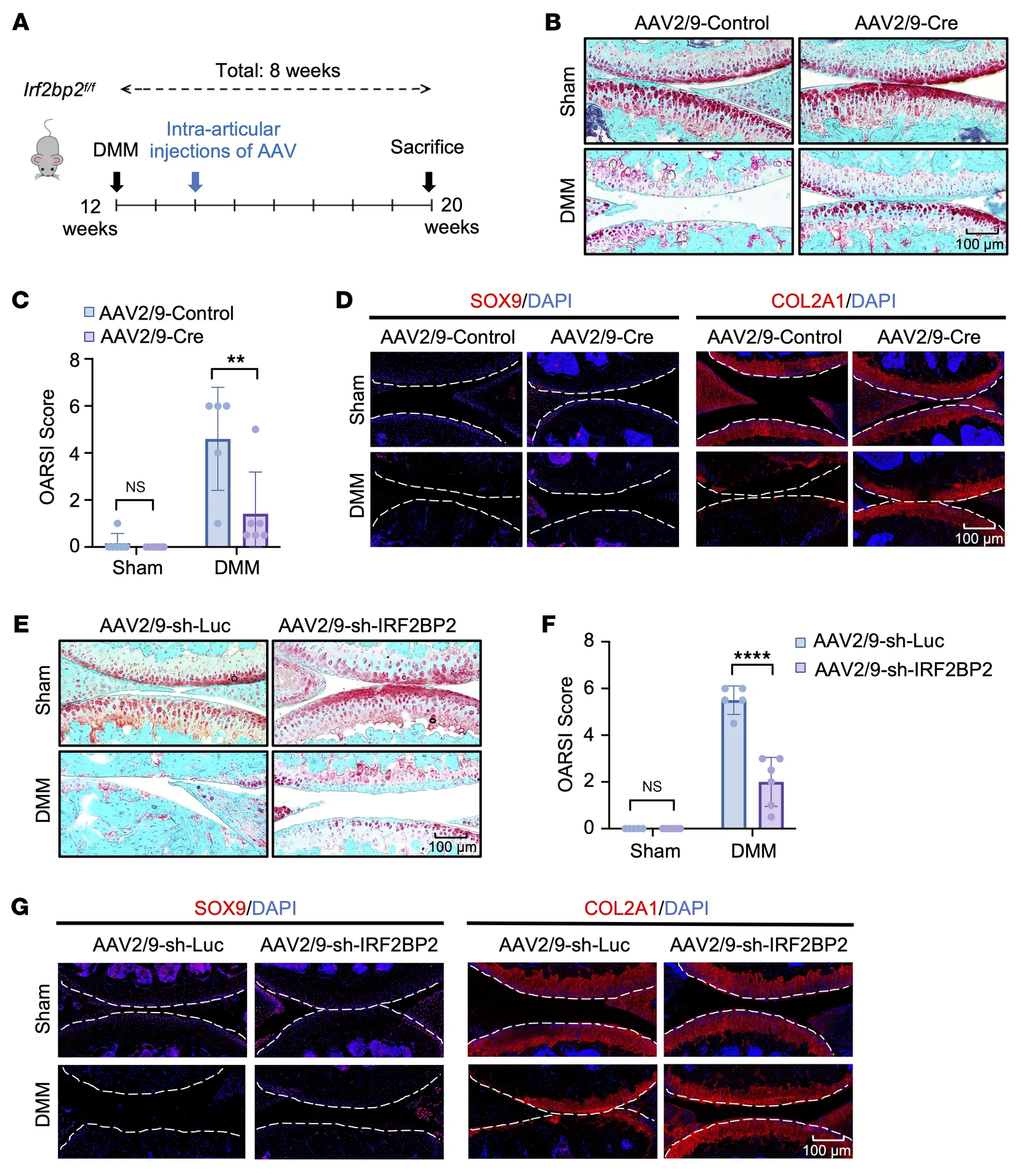
IRF2BP2 represents a promising therapeutic target for OA treatment. (**A**) Schematic outline of intra-articular injections of AAV (AAV2/9-control or AAV2/9-Cre and AAV2/9-sh-Luc or AAV2/9-sh-IRF2BP2, 1 × 10^13^ vg/mL) to knock out *Irf2bp2* after sham and DMM surgery. (**B**) SO staining of cartilage of the knee joints in *Irf2bp2^fl/fl^* mice following intra-articular injections of AAV2/9-control or AAV2/9-Cre after sham or DMM surgery. (**C**) OARSI scores for the samples in **B** (*n* = 6 mice per group, except for DMM-AAV2/9-control group, which had 5 mice per group). (**D**) IF staining for SOX9 and COL2A1 in *Irf2bp2^fl/fl^* mice with intra-articular injections of AAV2/9-control or AAV2/9-Cre after sham or DMM surgery. Scale bar: 100 μm. (**E**) SO staining of cartilage of the knee joints in *Irf2bp2^fl/fl^* mice following intra-articular injections of AAV2/9-sh-Luc or AAV2/9-sh-IRF2BP2 after sham or DMM surgery. Scale bar: 100 μm. (**F**) OARSI scores for the samples in **E** (*n* = 5 in the sham-AAV2/9-sh-Luc and DMM-AAV2/9-sh-Luc groups; *n* = 6 in the sham-AAV2/9-sh-IRF2BP2 and DMM-AAV2/9-sh-IRF2BP2 groups). (**G**) IF staining for SOX9 and COL2A1 in *Irf2bp2^fl/fl^* mice with intra-articular injections of AAV2/9-sh-Luc or AAV2/9-sh-IRF2BP2 after sham or DMM surgery. Scale bar: 100 μm. In **C** and **F**, data are shown as the mean ± SD. **P < 0.01 and ****P < 0.0001, by 2-way ANOVA, followed by Tukey’s multiple-comparison test (**C** and **F**). Dotted lines in **D** and **G** indicate the surface of articular cartilage.

**Figure 7:**
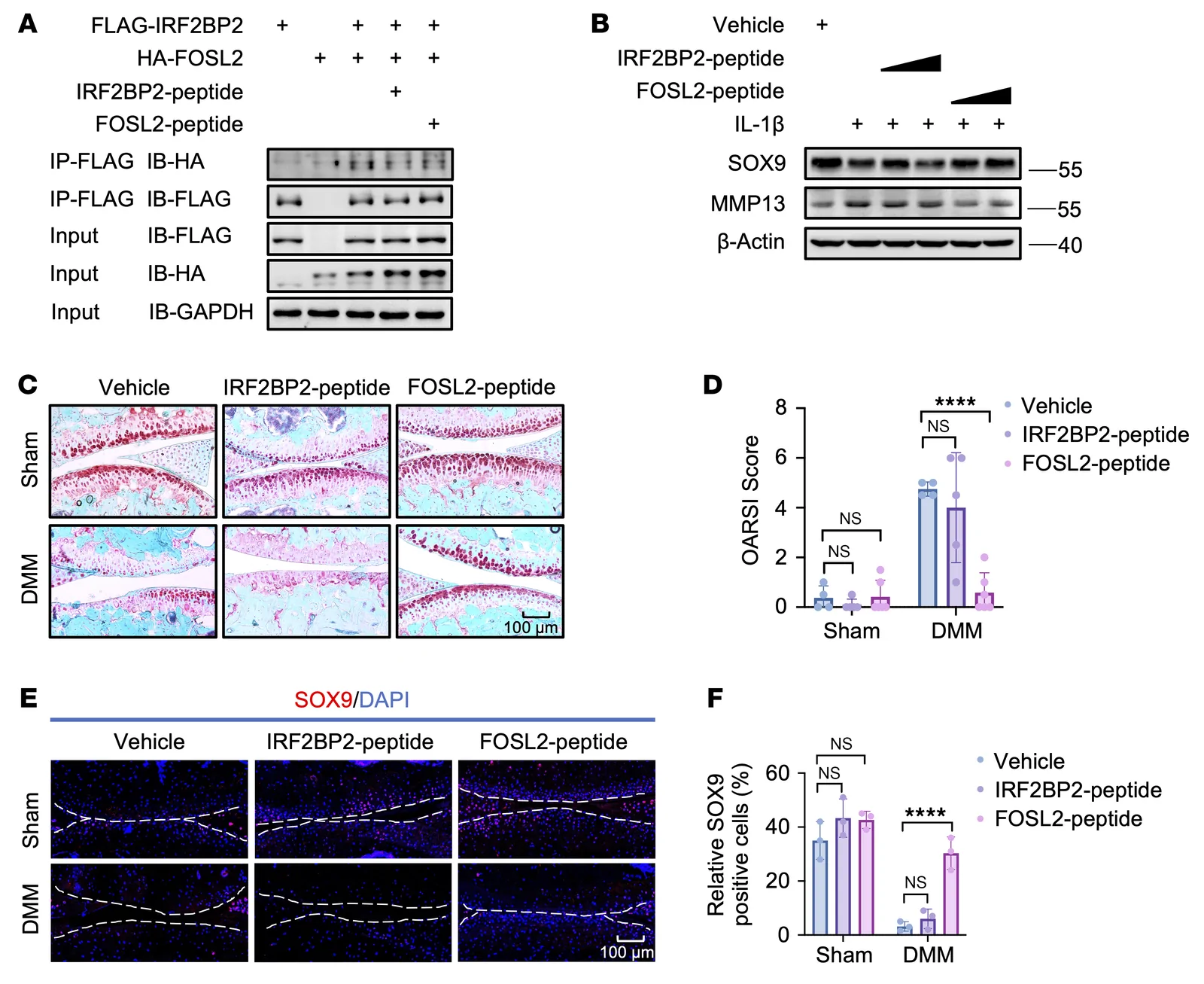
Therapeutic peptides targeting the IRF2BP2-FOSL2 interface attenuate OA progression. (**A**) Co-IP analysis of the competitive effects of IRF2BP2-peptide and FOSL2-peptide (4 μg/mL) on the interaction between IRF2BP2 and FOSL2 in HEK-293T cells. (**B**) Western blot analysis of SOX9 and MMP13 in ATDC5 cells treated with increasing concentrations of IRF2BP2-peptide or FOSL2-peptide (4 or 16 μg/mL) under IL-1β (10 ng/mL) stimulation. (**C**) SO staining of cartilage of the knee joints in WT mice following intra-articular injections of vehicle, IRF2BP2-peptide, or FOSL2-peptide (4 mg/mL) after sham or DMM surgery. Scale bar: 100 μm. (**D**) OARSI scores for the samples in **C** (*n* = 4 in the sham-vehicle and DMM-vehicle groups; *n* = 5 in the sham-IRF2BP2-peptide and DMM-IRF2BP2-peptide groups; *n* = 6 in the sham-FOSL2-peptide and DMM-FOSL2-peptide groups). (**E**) IF staining for SOX9 in WT mice with intra-articular injections of vehicle, IRF2BP2-peptide, or FOSL2-peptide after sham or DMM surgery. Scale bar: 100 μm. (**F**) Quantification of SOX9 expression in **E** (*n* = 3). In **D** and **F**, data are shown as the mean ± SD. ****P < 0.0001, by 2-way ANOVA, followed by Tukey’s multiple-comparison test (**D** and **F**). Dotted lines in **E** indicate the surface of articular cartilage.

**Figure 8:**
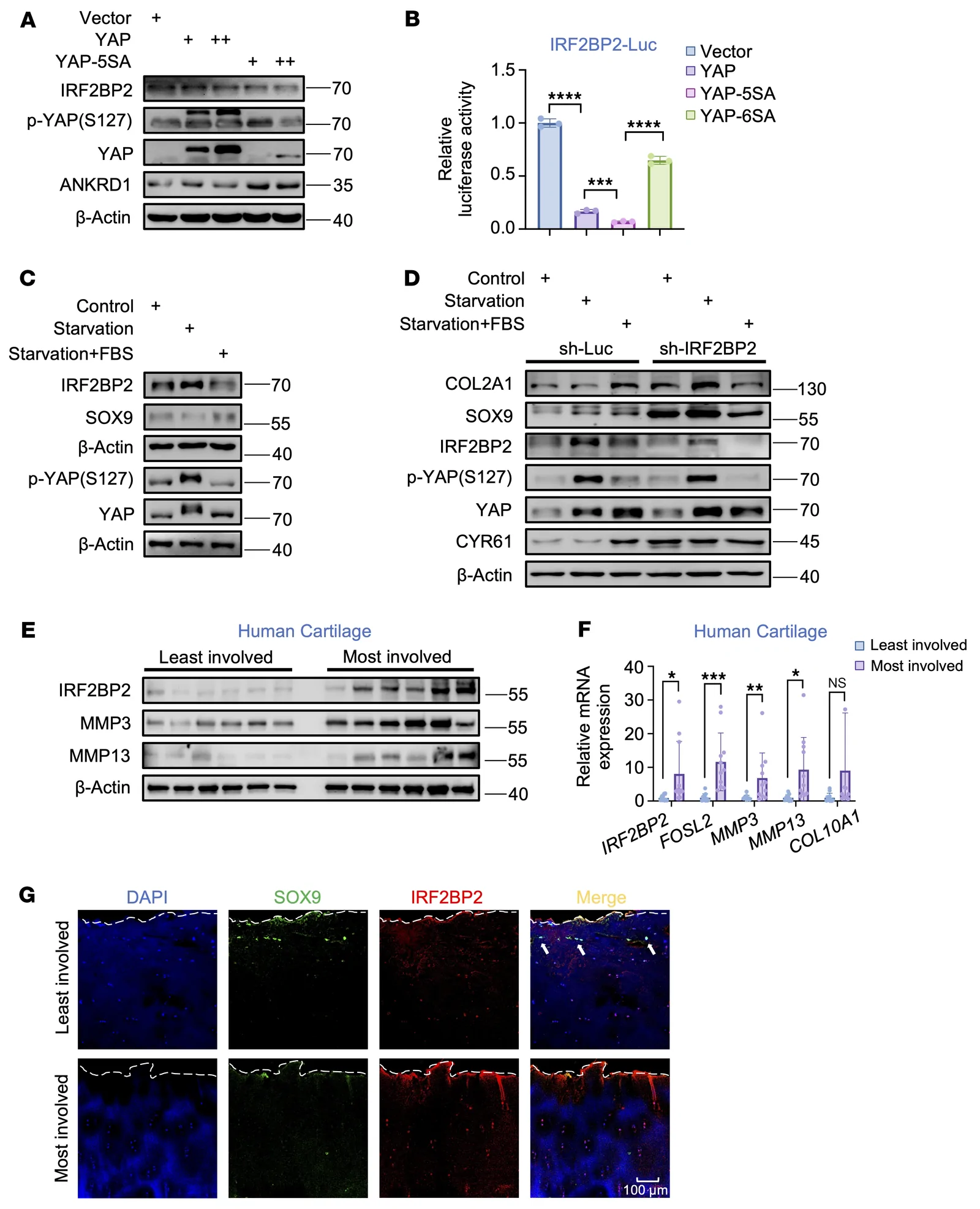
IRF2BP2 acts as a Hippo pathway hub component to modulate SOX9 expression and is upregulated in human osteoarthritic cartilage. (**A**) Western blot analysis of IRF2BP2 protein levels in ATDC5 cells transfected with increasing doses of YAP or YAP-5SA. (**B**) Luciferase reporter assays of *IRF2BP2* promoter activity in ATDC5 cells transfected with YAP, YAP-5SA, or YAP-6SA. (**C**) Western blot analysis of IRF2BP2 expression in ATDC5 cells following serum starvation (12 hours) and subsequent serum recovery (10% FBS, 1 hour). (**D**) Western blot analysis of SOX9 and COL2A1 protein levels in control and IRF2BP2-knockdown ATDC5 cells in response to serum recovery. (**E**) Western blot analysis of IRF2BP2, MMP3 and MMP13 expression in relatively intact and damaged regions of human osteoarthritic cartilage (*n* = 6). (**F**) qRT-PCR analysis of *IRF2BP2*, *FOSL2*, *MMP3*, *MMP13*, and *COL10A1* mRNA levels in relatively intact and damaged regions of human osteoarthritic cartilage (*n* = 11). (**G**) IF staining for SOX9 and IRF2BP2 in relatively intact and damaged regions of human osteoarthritic cartilage. Scale bar: 100 μm. In **B** and **F**, data are shown as the mean ± SD. *P < 0.05, **P < 0.01, ***P < 0.001, and ****P < 0.0001, by 1-way ANOVA, followed by Tukey’s multiple-comparison test (**B**) and 2-tailed, unpaired Student’s *t* test (**F**). Dotted lines in **G** indicate the surface of articular cartilage, and arrows indicate increased SOX9^+^ cells. p-YAP, phosphorylated YAP.

**Figure 9:**
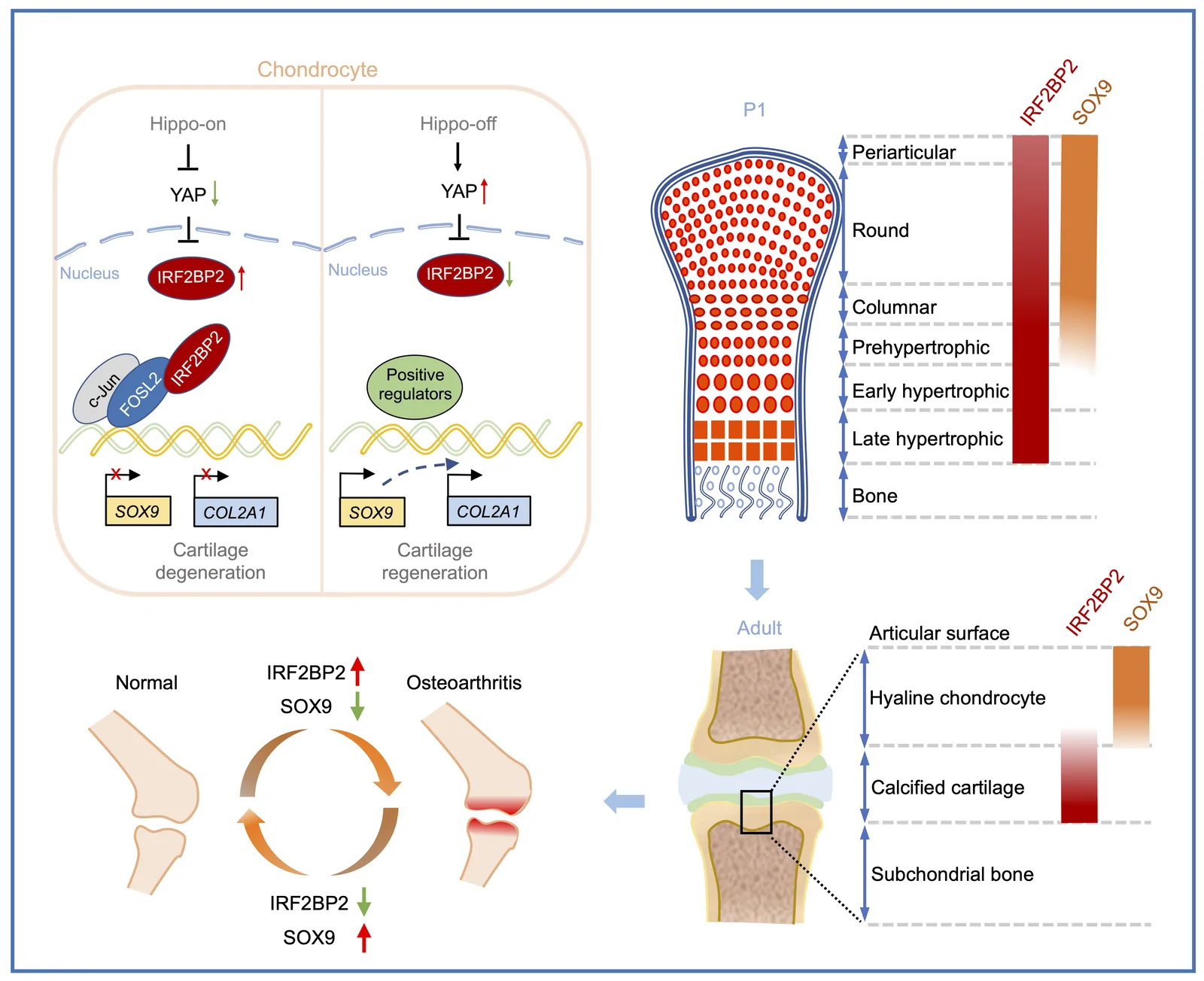
Schematic model representing the Hippo/IRF2BP2/FOSL2/SOX9 axis in cartilage homeostasis and OA progression. The model depicts 3 key aspects. Mechanistically, Hippo/YAP signaling transcriptionally regulates IRF2BP2, which stabilizes FOSL2 to enhance AP-1–mediated repression of SOX9 and COL2A1. Developmentally, in adult cartilage, IRF2BP2 is predominantly localized to the hypertrophic zone, while SOX9 is enriched in the hyaline cartilage zone, showing an inverse correlation. Therapeutically, in OA, IRF2BP2 is upregulated and SOX9 is downregulated. AAV-mediated IRF2BP2 knockdown and a FOSL2-derived peptide disrupting the IRF2BP2-FOSL2 interface restore SOX9 expression and attenuate OA progression.

## Version 1 10/15/2026

Electronic publication
