# Supplementary material for: IRF2BP2-FOSL2 interaction regulates SOX9-mediated cartilage regeneration in osteoarthritis models
Source: J Clin Invest. 2026 Oct 15;136(20):e200039. doi: 10.1172/JCI200039 (PMC13668630; doi:10.1172/JCI200039)
Supplement: Supplementary file 1 [file jci-136-200039-s064.pdf]

Supplemental Figures

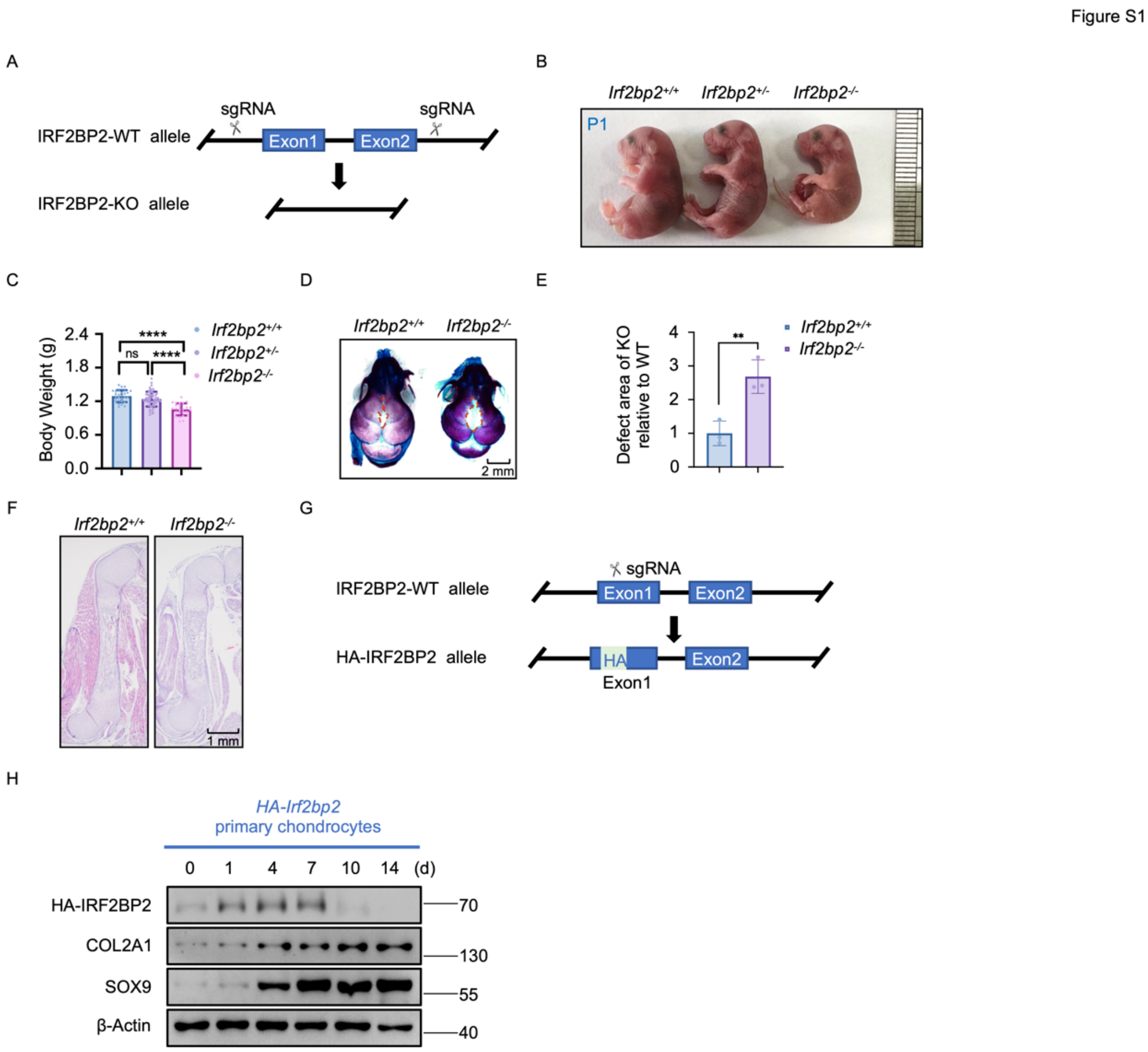

Figure S1 | Loss of *Irf2bp2* causes abnormal skeletal morphogenesis.

(A) Strategy for generating *Irf2bp2*$^{-/-}$ mice. **(B)** Representative images of WT, *Irf2bp2*$^{+/-}$, and *Irf2bp2*$^{-/-}$ mice at P1. **(C)** Quantification of body weight in (B) (n = 20). **(D)** The skull preparations from WT and *Irf2bp2*$^{-/-}$ mice were double-stained with Alizarin red and Alcian blue at P1. **(E)** Quantification of skull defect area in (D) (n = 3). **(F)** H&E staining of femur from WT and *Irf2bp2*$^{-/-}$ mice at P1. **(G)** Strategy for generating *HA-Irf2bp2* mice. **(H)** Western blot analysis of IRF2BP2 expression during chondrogenic differentiation in primary articular chondrocytes of *HA-Irf2bp2* mice. In (C) and (E), data are shown as mean ± SD. *p < 0.05, **p < 0.01, ***p < 0.001, ****p < 0.0001. ns: no significance. In (E), two-tailed unpaired Student's t-test. In (C), one-way ANOVA was used, followed by Tukey's multiple-comparisons test.

Figure S2

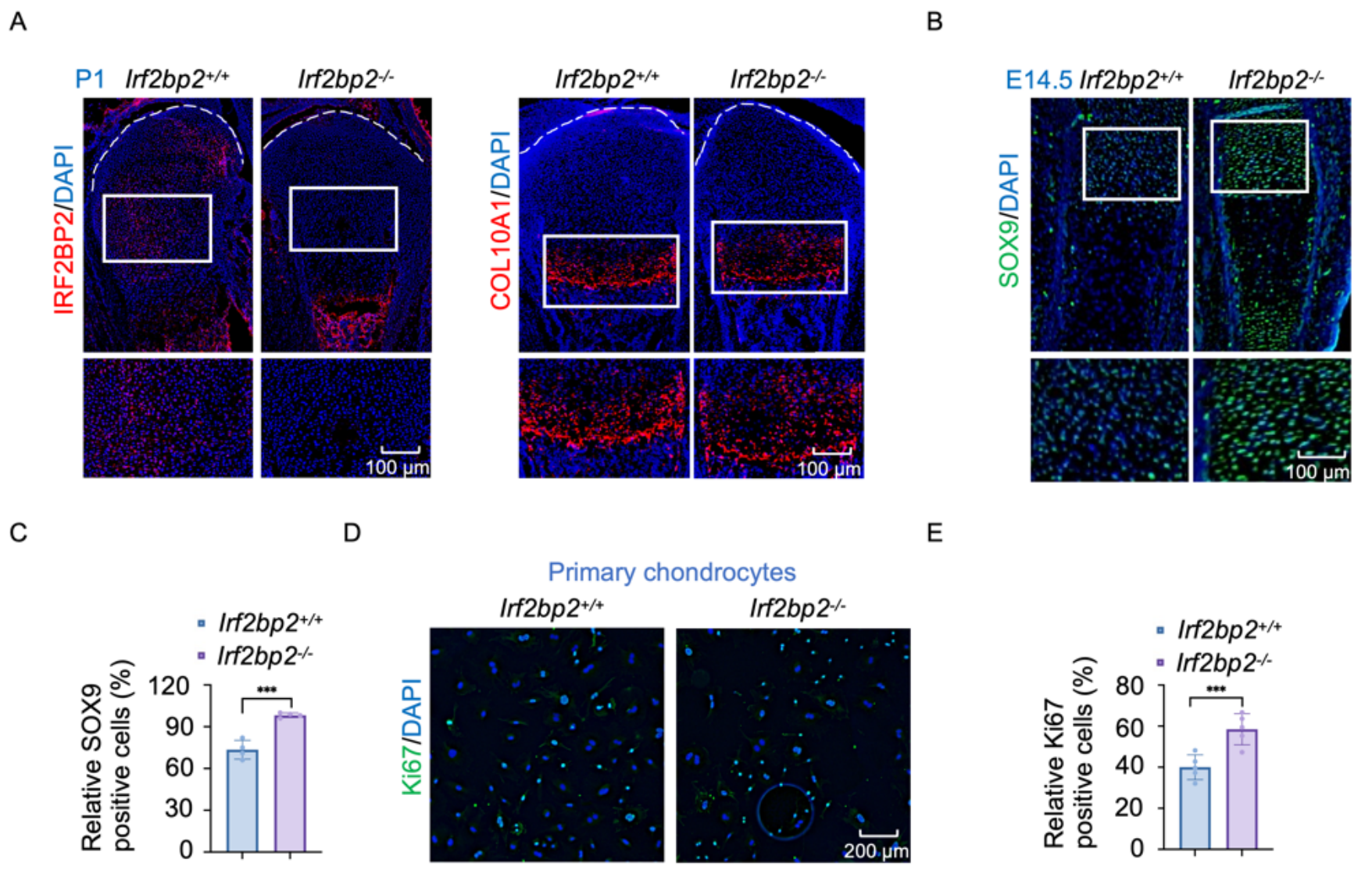

Figure S2 | Loss of *Irf2bp2* alters chondrocyte differentiation and proliferation.

(A) IF staining of IRF2BP2 and COL10A1 in WT and *Irf2bp2*$^{-/-}$ mice at P1. **(B)** IF staining of SOX9 in WT and *Irf2bp2*$^{-/-}$ mice at E14.5. **(C)** Quantification of SOX9 expression in (B) (n = 4). **(D)** IF staining of Ki67 in primary articular chondrocytes of WT and *Irf2bp2*$^{-/-}$ mice at P1. **(E)** Quantification of Ki67 expression in (D) (n = 4). In (C) and (E), data are shown as mean ± SD. *p < 0.05, **p < 0.01, ***p < 0.001, ****p < 0.0001. ns: no significance. Two-tailed unpaired Student's t-test. In (A), dotted lines indicate the surface of articular cartilage.

Figure S3

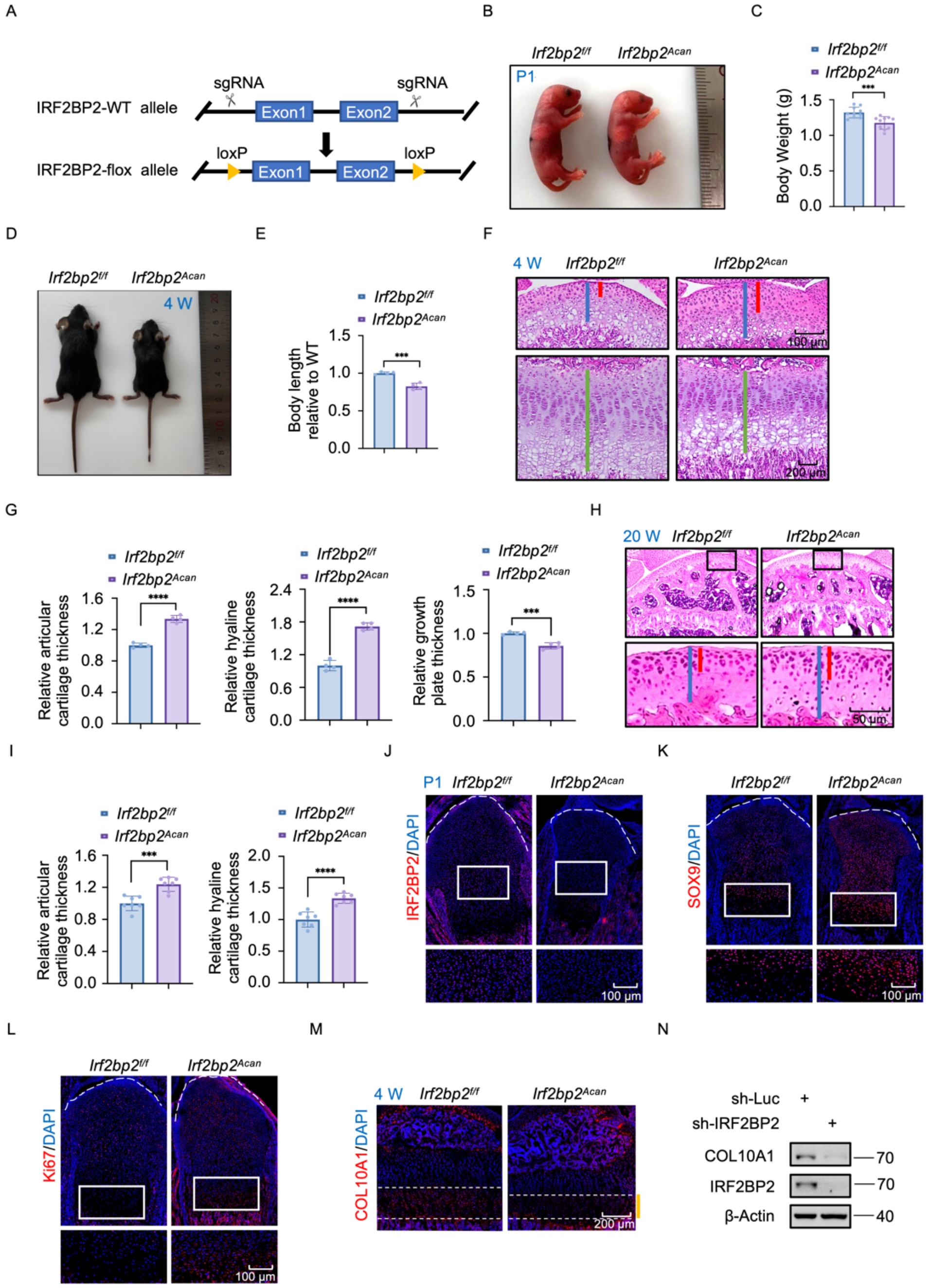

Figure S3 | Deficiency of *Irf2bp2* in cartilage induces ectopic expression of SOX9 in the hypertrophic zone and increases the thickness of articular cartilage.

(A) Strategy for generating *Irf2bp2*$^{f/f}$ mice. **(B)** Representative images of *Irf2bp2*$^{f/f}$ and *Irf2bp2*^{Acan} mice at P1. **(C)** Quantification of body weight in (B) (n = 10). **(D)** Representative view of *Irf2bp2*$^{f/f}$ and *Irf2bp2*^{Acan} mice at 4 weeks. **(E)** Quantification of

body length in (D) (n = 4). **(F)** H&E staining of articular cartilage of *Irf2bp2*$^{f/f}$ and *Irf2bp2*^{Acan} mice at 4 weeks. **(G)** Quantification of articular cartilage thickness, hyaline cartilage thickness, and growth plate thickness in (F) (n = 4). **(H)** H&E staining of articular cartilage of *Irf2bp2*$^{f/f}$ and *Irf2bp2*^{Acan} mice at 20 weeks. **(I)** Quantification of articular cartilage thickness and hyaline cartilage thickness in (H) (n = 6). **(J-L)** IF staining of IRF2BP2 (J), SOX9 (K), and Ki67 (L) in *Irf2bp2*$^{f/f}$ and *Irf2bp2*^{Acan} mice at P1. **(M)** IF staining of COL10A1 in *Irf2bp2*$^{f/f}$ and *Irf2bp2*^{Acan} mice at 4 weeks. **(N)** Western blot analysis of COL10A1 and IRF2BP2 in control (sh-Luc) and IRF2BP2-knockdown (sh-IRF2BP2) ATDC5 stable cells. In (C), (E), (G), and (I), data are shown as mean ± SD. *p < 0.05, **p < 0.01, ***p < 0.001, ****p < 0.0001. ns: no significance. Two-tailed unpaired Student's t-test. In (J-L), dotted lines indicate the surface of articular cartilage. In (F), (H), and (M), color bars indicate distinct skeletal zones, blue: articular cartilage; red: hyaline cartilage; green: growth plate; orange: prehypertrophic and hypertrophic chondrocytes.

Figure S4

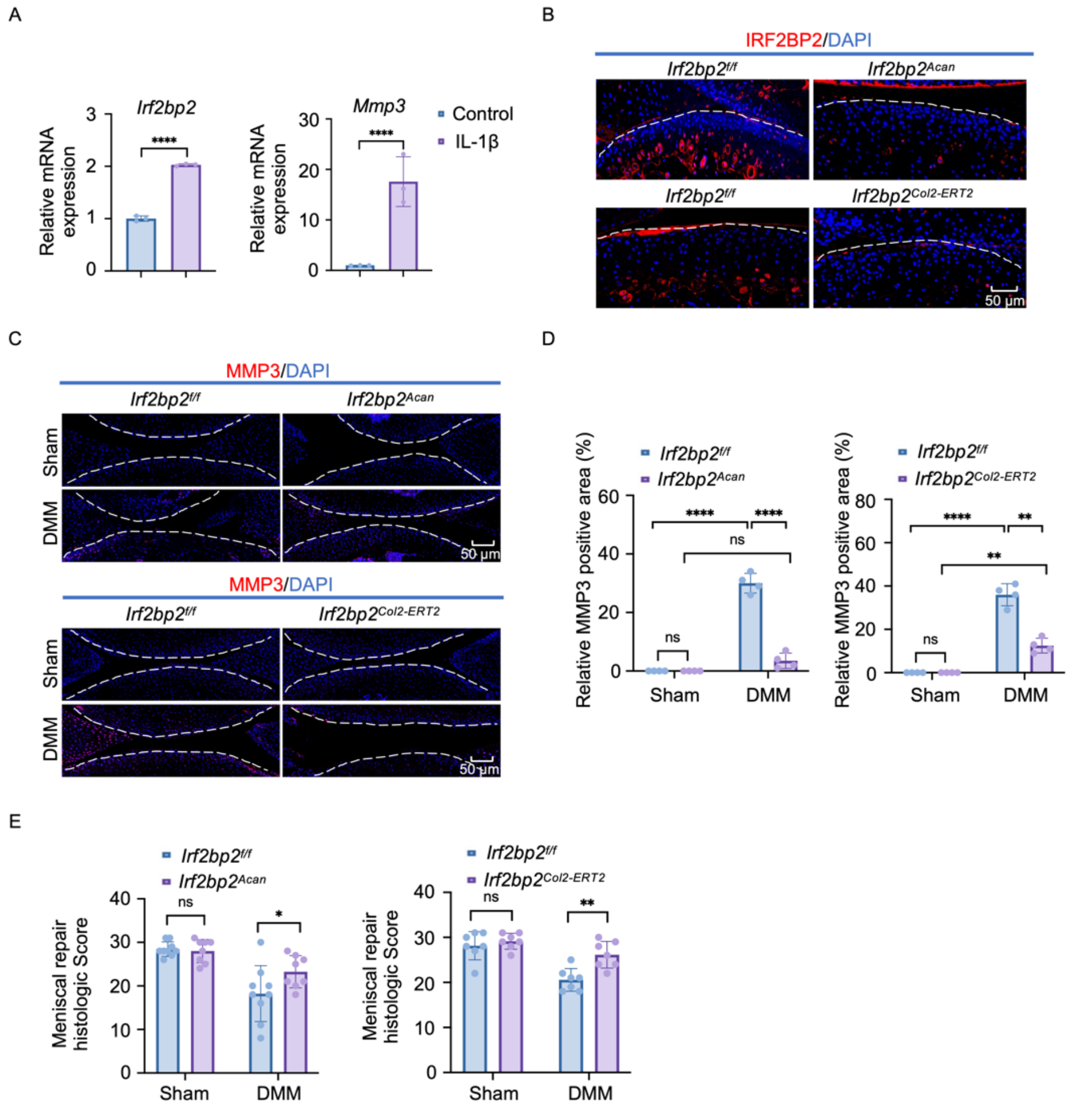

Figure S4 | IRF2BP2 expression is upregulated with IL-1β treatment in vitro and in the mouse model of OA.

(A) qRT-PCR analysis of *Irf2bp2* and *Mmp3* mRNA levels in ATDC5 cells stimulated with IL-1β (10 ng/mL) for 24 h. **(B)** IF staining of IRF2BP2 in *Irf2bp2*$^{f/f}$, *Irf2bp2*^{Acan}, and *Irf2bp2*$^{Col2\text{-}ERT2}$ mice at 20 weeks. **(C)** IF staining of MMP3 in *Irf2bp2*$^{f/f}$, *Irf2bp2*^{Acan}, and *Irf2bp2*$^{Col2\text{-}ERT2}$ mice after Sham or DMM surgery. **(D)** Quantification of MMP3 expression in (C) (n = 4). **(E)** Meniscal repair scores in *Irf2bp2*$^{f/f}$ and *Irf2bp2*^{Acan} mice (left) or *Irf2bp2*$^{f/f}$ and *Irf2bp2*$^{Col2\text{-}ERT2}$ mice (right) after Sham or DMM surgery (n = 7). In (A), (D), and (E), data are shown as mean ± SD. *$p < 0.05$, **$p < 0.01$, ***$p < 0.001$, ****$p < 0.0001$. ns: no significance. In (A), two-tailed unpaired Student's t-test. In (D) and (E), two-way ANOVA was used, followed by Tukey's multiple-comparisons test. In (B) and (C), dotted lines indicate the surface of articular cartilage.

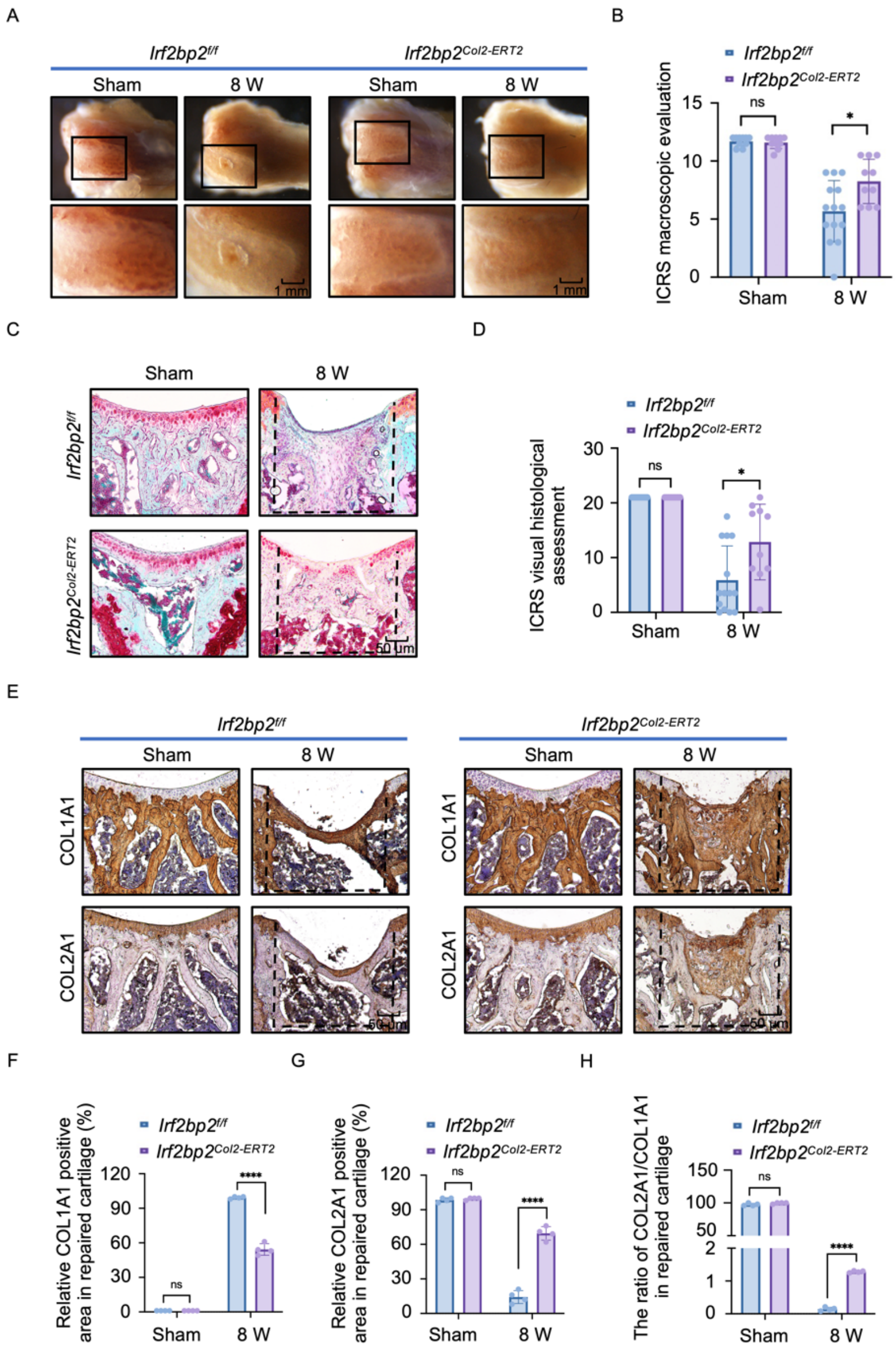

Figure S5 | Knockout *Irf2bp2* promotes cartilage regeneration in *Irf2bp2*$^{Col2\text{-}ERT2}$ mice.

(A) Representative images of distal femur from Sham- or MF-operated *Irf2bp2*$^{f/f}$ and *Irf2bp2*$^{Col2\text{-}ERT2}$ mice at 8 weeks after surgery. **(B)** ICRS macroscopic evaluation scores assessment in (A) (n = 10). **(C)** S.O. staining of the articular cartilage defect sections. **(D)** ICRS visual histological scores of regenerated tissues in (C) (n = 10). **(E)** Representative images of IHC staining for COL1A1 and COL2A1 in articular cartilage defect sections. **(F and G)** Quantification of the expression of COL1A1 (F) and

COL2A1 (G) in articular cartilage defect sections in (E) (n = 4). **(H)** Quantification of the COL2A1/COL1A1 intensity ratio in repaired cartilage in (E) (n = 4). In (B), (D), and (F-H), data are shown as mean ± SD. *$p < 0.05$, **$p < 0.01$, ***$p < 0.001$, ****$p < 0.0001$. ns: no significance. Two-way ANOVA was used, followed by Tukey's multiple-comparisons test. In (C) and (E), the region of interest (ROI) is indicated by the black dotted lines.

Figure S6

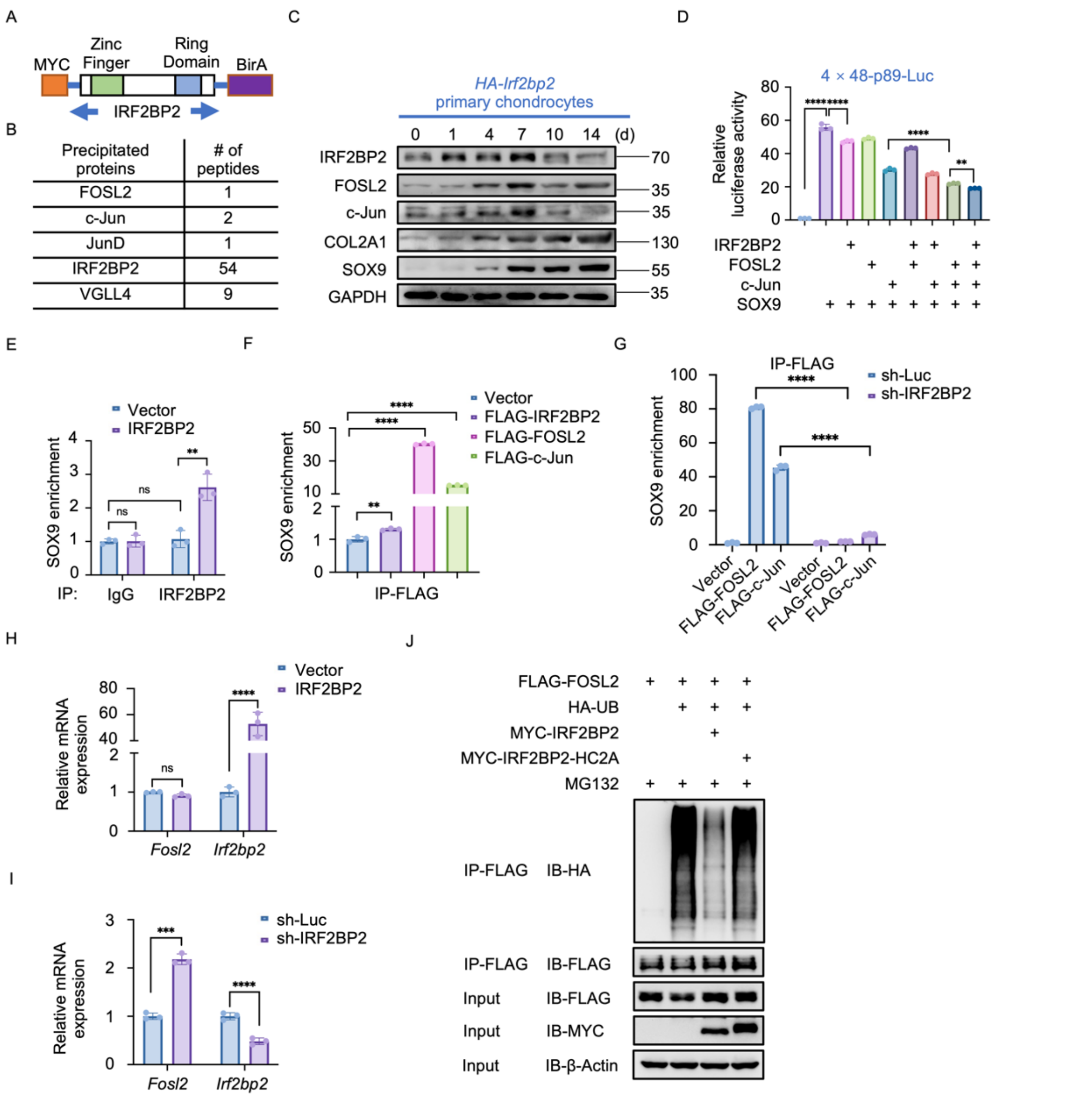

Figure S6 | Identify the interaction proteins of IRF2BP2.

(A) Schematic illustration of MYC-IRF2BP2-BirA. **(B)** Some of the potential interaction proteins of IRF2BP2 by mass spectrometry analysis. **(C)** Western blot analysis of IRF2BP2, FOSL2, and c-Jun expression during chondrocyte differentiation in primary articular chondrocytes of *HA-Irf2bp2* mice. **(D)** 4 × 48-p89-Luc activity was determined in ATDC5 cells. **(E)** ChIP-qPCR analysis of the direct association of IRF2BP2 with the *Sox9* promoter. **(F)** ChIP-qPCR analysis of the FLAG-IRF2BP2, FLAG-FOSL2, or FLAG-c-Jun with the *Sox9* promoter. **(G)** ChIP-qPCR analysis of the FLAG-FOSL2 or FLAG-c-Jun with the *Sox9* promoter in sh-Luc and sh-IRF2BP2 ATDC5 cells. **(H and I)** qRT-PCR analysis of *Fosl2* and *Irf2bp2* expression in ATDC5 cells when

overexpression (H) or knockdown (I) IRF2BP2. **(J)** Ubiquitination assay of FLAG-FOSL2 was performed in ATDC5 cells. In (D-I), data are shown as mean ± SD. *$p < 0.05$, **$p < 0.01$, ***$p < 0.001$, ****$p < 0.0001$. ns: no significance. In (H) and (I), two-tailed unpaired Student's t-test. In (D) and (F), one-way ANOVA was used, followed by Tukey's multiple-comparisons test. In (E) and (G), two-way ANOVA was used, followed by Tukey's multiple-comparisons test.

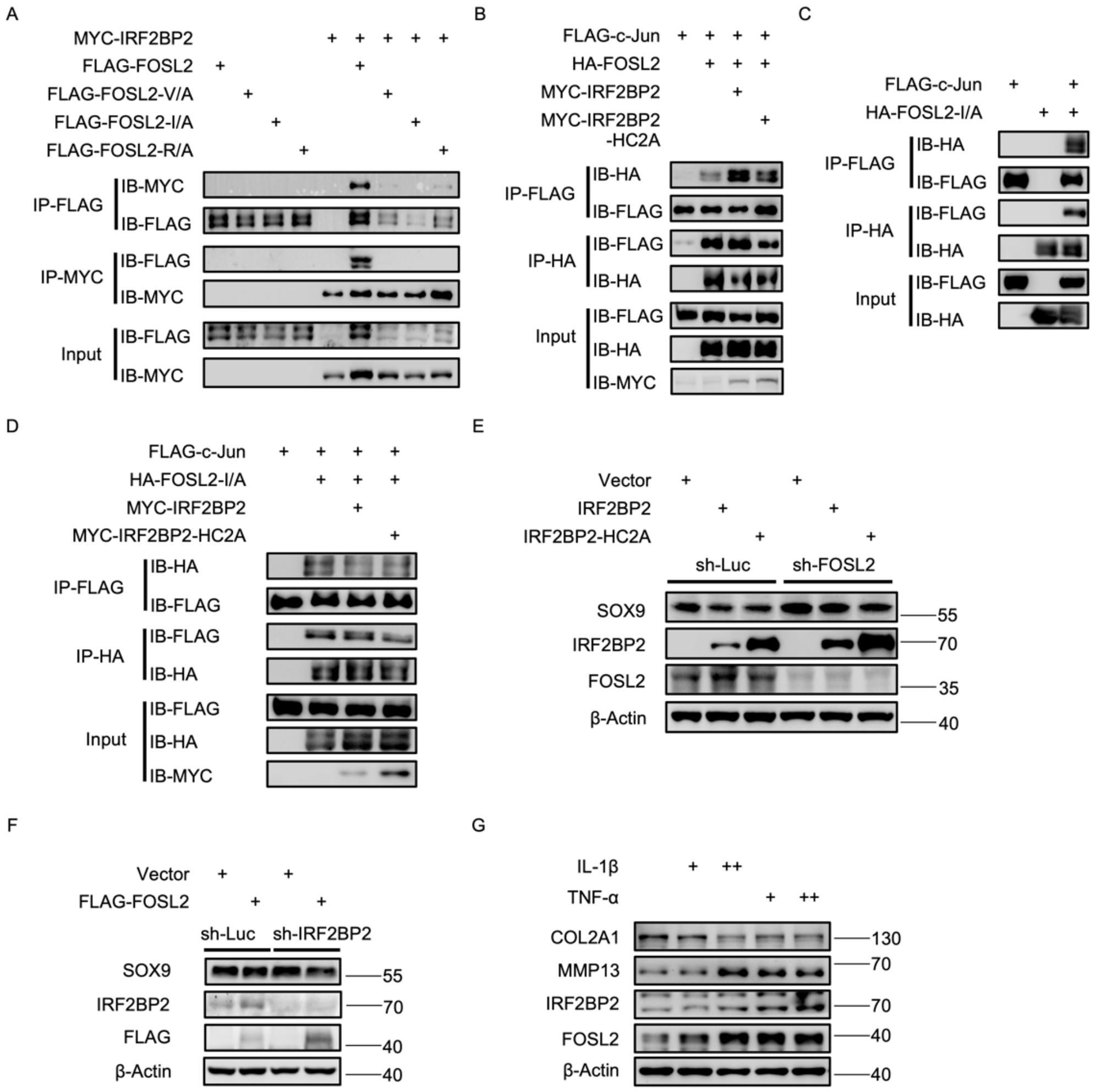

Figure S7 | IRF2BP2 regulates SOX9 activity in a FOSL2-dependent manner.

(A) Co-IP analysis of IRF2BP2 interaction with FOSL2 and FOSL2 mutants in HEK-293T cells. **(B)** Co-IP experiment of c-Jun, FOSL2, and IRF2BP2-WT/HC2A in HEK-293T cells. **(C)** Co-IP experiment of c-Jun and FOSL2-I/A in HEK-293T cells. **(D)** Co-IP experiment of IRF2BP2-WT/HC2A, FOSL2-WT/mutants, and c-Jun in HEK-293T cells. **(E)** Western blot analysis of SOX9, IRF2BP2, and FOSL2 in sh-Luc and sh-FOSL2 ATDC5 cells. **(F)** Western blot analysis of SOX9 in sh-Luc and sh-IRF2BP2 ATDC5 cells transfected with FOSL2-WT or vector. **(G)** Western blot analysis of FOSL2 and IRF2BP2 in ATDC5 cells stimulated with IL-1β or TNF-α (5 or 10 ng/mL) for 24 h.

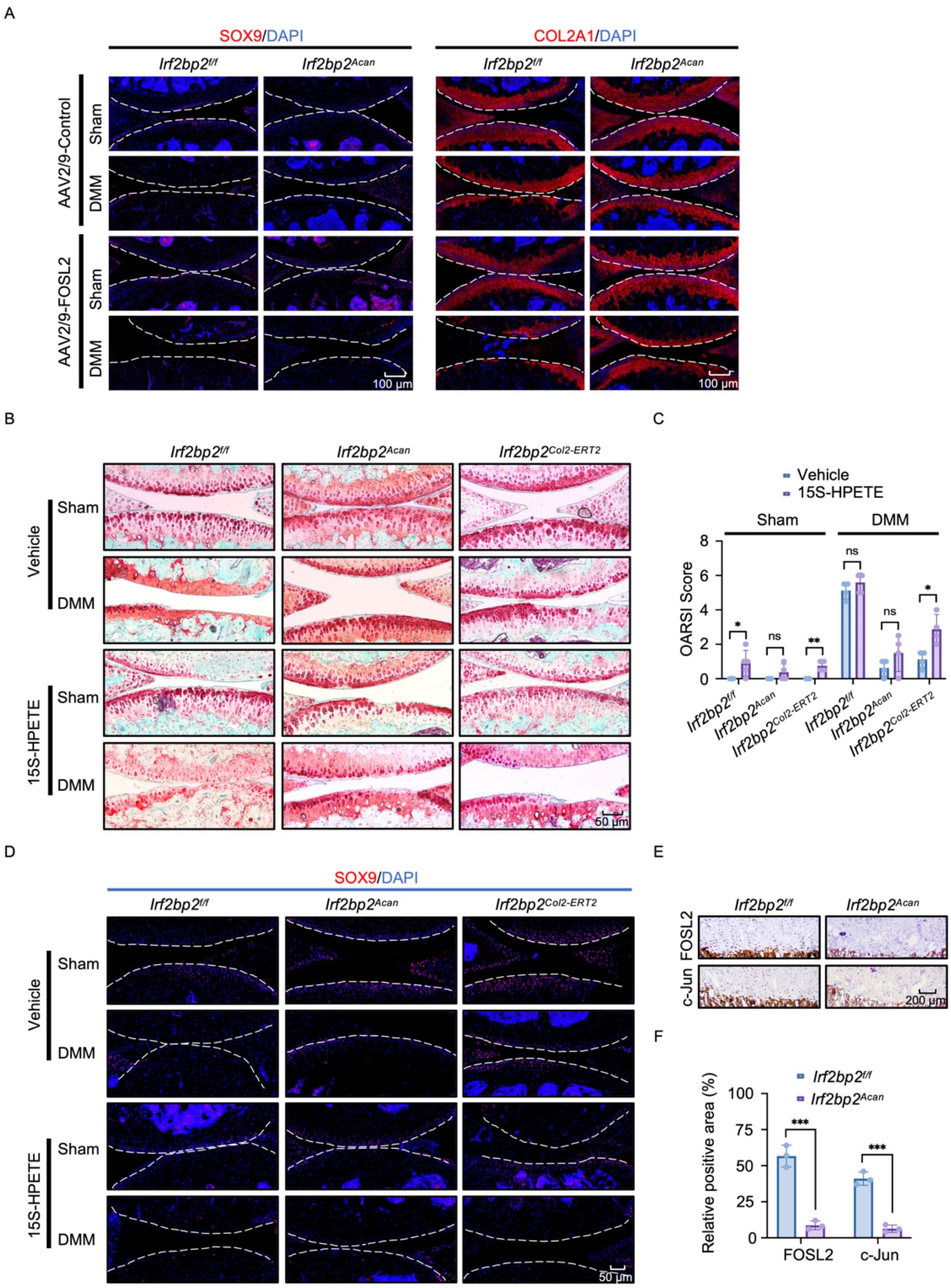

Figure S8 | IRF2BP2 regulates OA progression in a FOSL2-dependent manner in vivo.

(A) IF staining of SOX9 in cartilage of the knee joints of *Irf2bp2*$^{f/f}$ and *Irf2bp2*^{Acan} mice treated with AAV2/9-Control or AAV2/9-FOSL2 after Sham or DMM surgery. **(B)** S.O. staining of cartilage of the knee joints in *Irf2bp2*$^{f/f}$, *Irf2bp2*^{Acan} and *Irf2bp2*$^{Col2\text{-}ERT2}$ mice treated with vehicle or 15S-HPETE (10 μg/mL) after Sham or DMM surgery. **(C)** OARSI

scores for samples shown in (B) (n = 4 mice per group, except for *Irf2bp2*$^{f/f}$-Sham-15S-HPETE and *Irf2bp2*$^{f/f}$-DMM-15S-HPETE groups, which had n = 5). **(D)** IF staining of SOX9 in cartilage of the knee joints from samples shown in (B). **(E)** Representative images of IHC staining for FOSL2 and c-Jun in *Irf2bp2*$^{f/f}$ and *Irf2bp2*^{Acan} mice at 4 weeks. **(F)** Quantification of FOSL2 and c-Jun expression in (E) (n = 4). In (C) and (F), data are shown as mean ± SD. *p < 0.05, **p < 0.01, ***p < 0.001, ****p < 0.0001. ns: no significance. In (F), two-tailed unpaired Student's t-test. In (C), two-way ANOVA was used, followed by Tukey's multiple-comparisons test. In (A) and (D), dotted lines indicate the surface of articular cartilage.

Figure S9

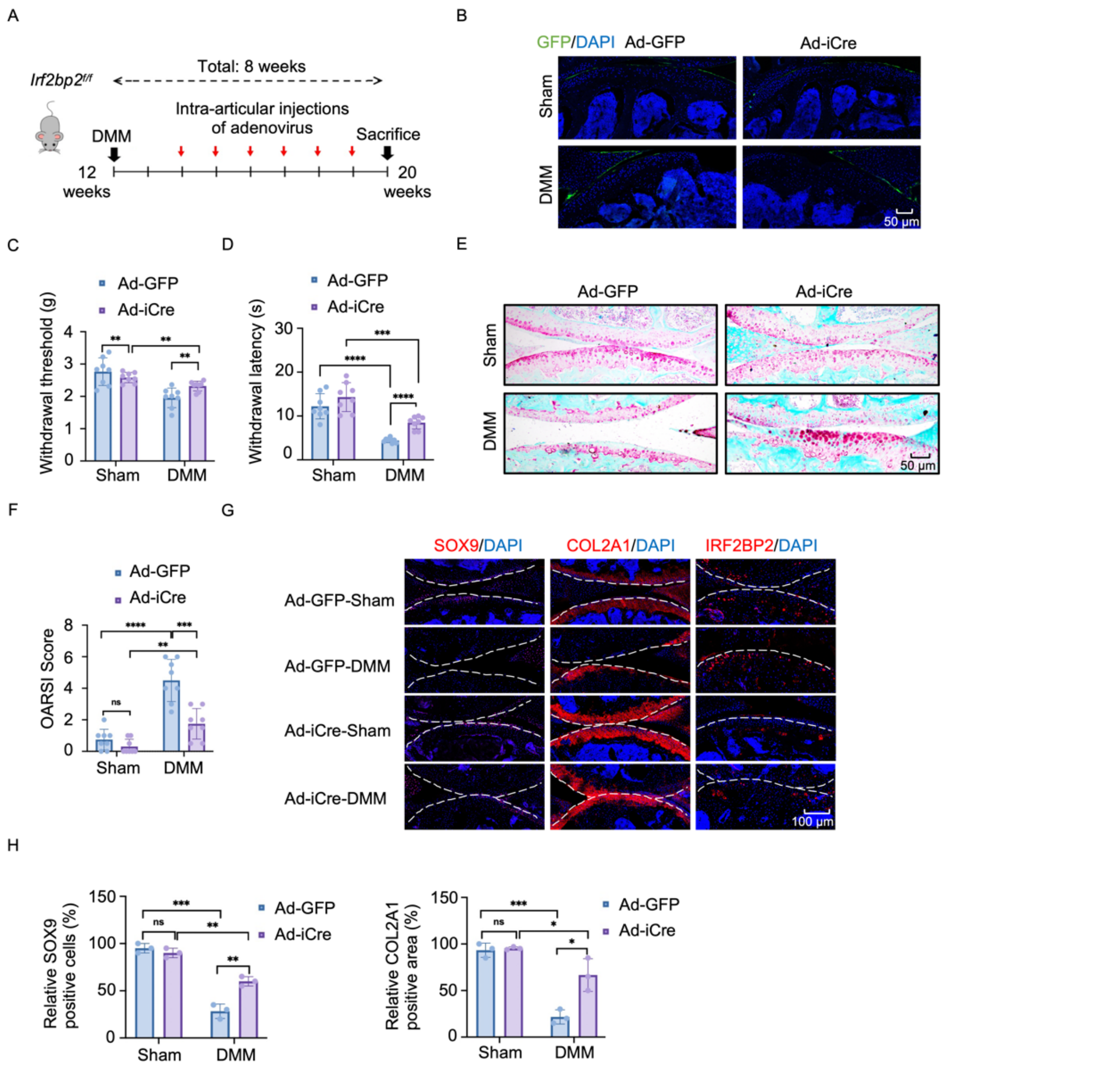

Figure S9 | Adenovirus-mediated *Irf2bp2* knockdown alleviates OA progression.

(A) Schematic outline of intra-articular injections of adenovirus (Ad-GFP or Ad-iCre, 1 × 10^{11} vg/mL) to knockout *Irf2bp2* after Sham and DMM surgery. **(B)** IF staining of GFP in *Irf2bp2*$^{f/f}$ mice following intra-articular injections of Ad-GFP or Ad-iCre after Sham or DMM surgery. **(C and D)** The pain was measured by von Frey assay (C) and radiant heat paw-withdrawal test (D) in *Irf2bp2*$^{f/f}$ mice following intra-articular injections of Ad-GFP or Ad-iCre after Sham or DMM surgery (n = 8). **(E)** S.O. staining of cartilage of the knee joints in *Irf2bp2*$^{f/f}$ mice following intra-articular injections of Ad-GFP or Ad-iCre after Sham or DMM surgery. **(F)** OARSI scores of samples in (E) (n = 8). **(G)** IF staining of SOX9, COL2A1, and IRF2BP2 in *Irf2bp2*$^{f/f}$ mice following intra-articular injections of

Ad-GFP or Ad-iCre after Sham or DMM surgery. **(H)** Quantification of SOX9 and COL2A1 expression in (G) (n = 4). In (C), (D), (F), and (H), data are shown as mean ± SD. *p < 0.05, **p < 0.01, ***p < 0.001, ****p < 0.0001. ns: no significance. Two-way ANOVA was used, followed by Tukey's multiple-comparisons test. In (G), dotted lines indicate the surface of articular cartilage.

Figure S10

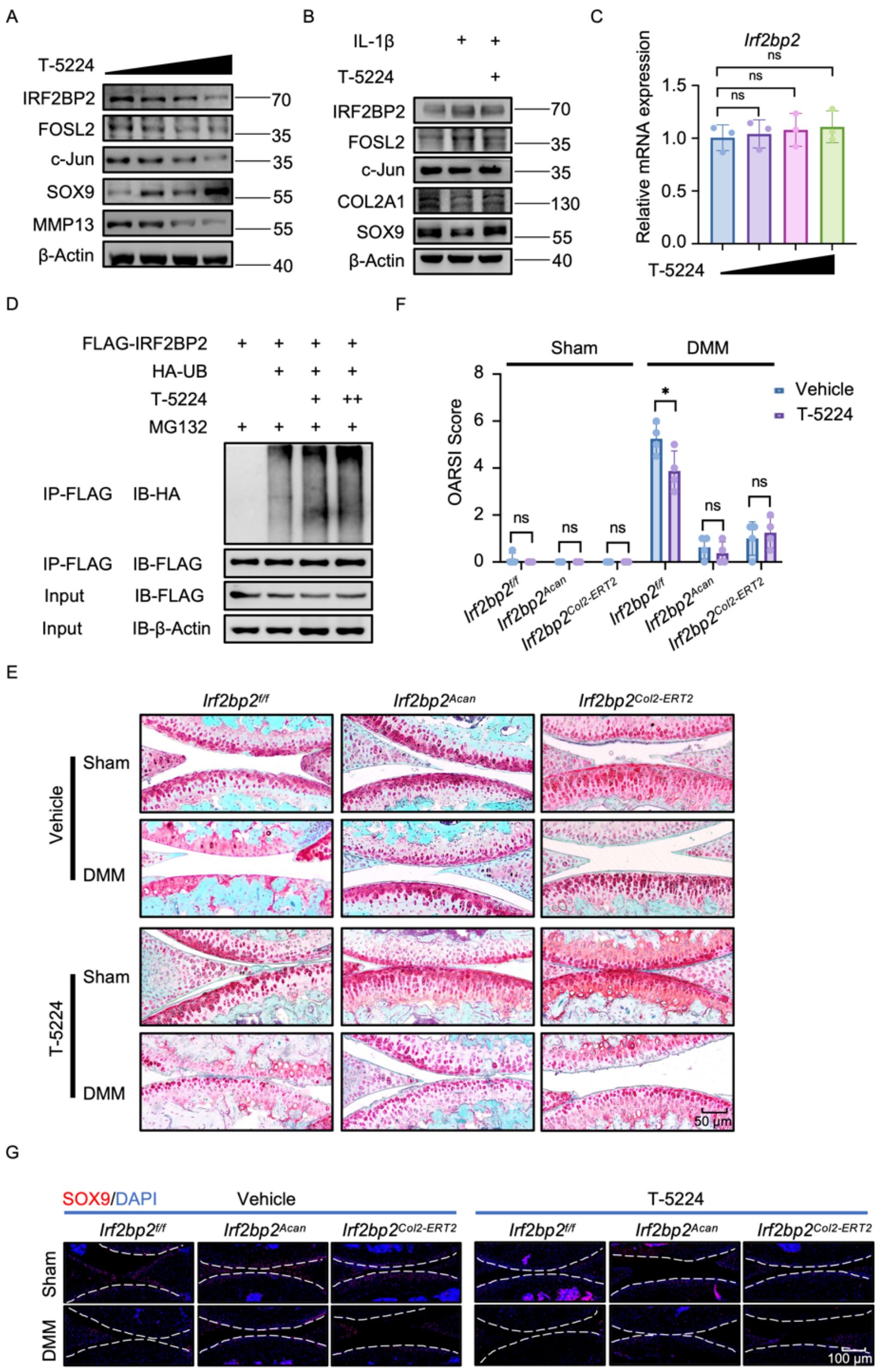

Figure S10 | T-5224 suppresses IRF2BP2 expression to promote cartilage repair.

(A) Western blot analysis of IRF2BP2, FOSL2, c-Jun, SOX9 and MMP13 protein levels in ATDC5 cells treated with increasing concentrations of T-5224 for 24 hours. **(B)** Western blot analysis of the effects of T-5224 (10 μM) on IL-1β (10 ng/mL) treatment in ATDC5 cells. **(C)** qRT-PCR analysis of *Irf2bp2* expression in ATDC5 cells following increasing concentrations of T-5224. **(D)** Ubiquitination assay of FLAG-IRF2BP2 in

ATDC5 cells following T-5224 treatment. **(E)** S.O. staining of cartilage of the knee joints in *Irf2bp2*$^{f/f}$, *Irf2bp2*^{Acan} and *Irf2bp2*$^{Col2\text{-}ERT2}$ mice treated with vehicle or T-5224 (10 mM) after Sham or DMM surgery. **(F)** OARSI scores of samples in (E). **(G)** IF staining of SOX9 in cartilage of the knee joints of *Irf2bp2*$^{f/f}$, *Irf2bp2*^{Acan} and *Irf2bp2*$^{Col2\text{-}ERT2}$ mice treated with vehicle or T-5224 after Sham or DMM surgery. In (C) and (F), data are shown as mean ± SD. *$p < 0.05$, **$p < 0.01$, ***$p < 0.001$, ****$p < 0.0001$. ns: no significance. In (C), one-way ANOVA was used, followed by Tukey's multiple-comparisons test. In (F), two-way ANOVA was used, followed by Tukey's multiple-comparisons test. In (G), dotted lines indicate the surface of articular cartilage.

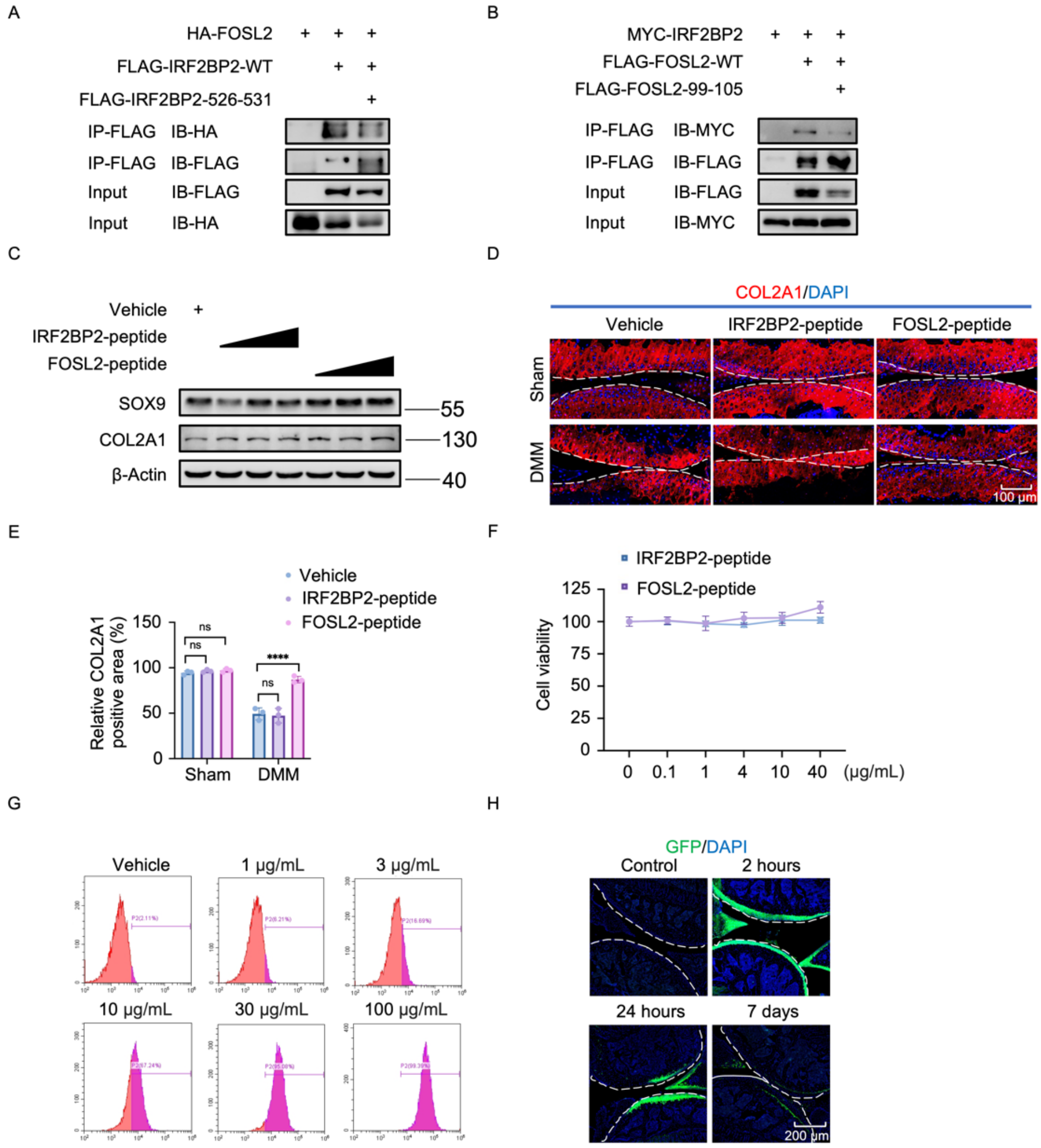

Figure S11 | Therapeutic peptides targeting the IRF2BP2-FOSL2 interface.

(A and B) Co-IP analysis of the inhibitory effects of IRF2BP2 truncation (A) and FOSL2 truncation (B) on the interaction between IRF2BP2 and FOSL2. **(C)** Western blot analysis of SOX9 and COL2A1 in ATDC5 cells treated with IRF2BP2-peptide or FOSL2-peptide (1, 4, and 16 μg/mL). **(D)** IF staining of COL2A1 in cartilage of the knee joints of mice treated with vehicle, IRF2BP2-peptide or FOSL2-peptide after Sham or DMM surgery. **(E)** Quantification of COL2A1 expression in (D). **(F)** Cell viability of ATDC5 cells treated with IRF2BP2-peptide or FOSL2-peptide for 48 h, assessed by CCK-8 assay. **(G)** Flow cytometry analysis of FITC-positive ATDC5 cells after 30 min incubation with FITC-labeled FOSL2-peptide. **(H)** IF staining images of knee joint

cartilage at 2 h, 24 h, and 7 d after intra-articular injections of FITC-labeled FOSL2-peptide (10 mg/mL). In (E) and (F) data are shown as mean ± SD. *p < 0.05, **p < 0.01, ***p < 0.001, ****p < 0.0001. ns: no significance. In (E), two-way ANOVA was used, followed by Tukey's multiple-comparisons test. In (D) and (H), dotted lines indicate the surface of articular cartilage.

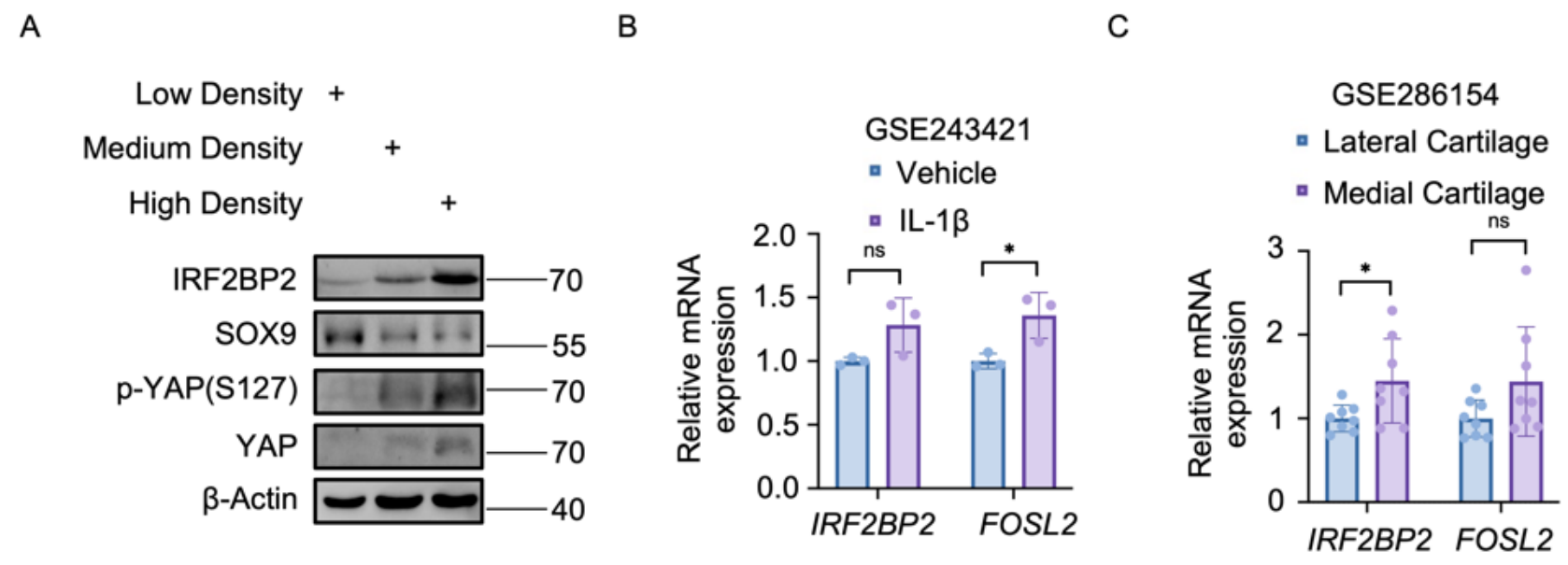

Figure S12 | IRF2BP2 expression is increased in human OA cartilage.

(A) Western blot analysis of IRF2BP2 expression in ATDC5 cells at low, medium, and high densities. **(B)** RNA-seq analysis of *IRF2BP2* and *FOSL2* mRNA levels in human chondrocytes treated with IL-1β (data from GEO Series GSE243421). **(C)** RNA-seq analysis of *IRF2BP2* and *FOSL2* mRNA levels in human OA cartilage from lateral cartilage and medial cartilage (data from GEO Series GSE286154). In (B) and (C) data are shown as mean ± SD. *$p < 0.05$, **$p < 0.01$, ***$p < 0.001$, ****$p < 0.0001$. ns: no significance. Two-tailed unpaired Student's t-test.

Supplemental Materials and Methods

Mouse Lines

Irf2bp2 knockout mice were generated by CRISPR/Cas9-mediated editing. Two single-guide RNAs targeting exon 1 and exon 2 of *Irf2bp2* were designed and co-injected with Cas9 into mouse zygotes to generate null alleles. *Irf2bp2* knockout mice were originally obtained from GemPharmatech Co., Ltd (Jiangsu, China).

Irf2bp2 floxed mice were generated by CRISPR/Cas9 to insert LoxP sites flanking exons 1-2 in the C57BL/6J mouse strain. *Irf2bp2* floxed mice were originally obtained from GemPharmatech Co., Ltd (Jiangsu, China).

HA-Irf2bp2 knock-in mice were generated through CRISPR/Cas9-mediated genome editing by the Chinese Academy of Sciences Genome Tagging Project (GTP, No. 20001016).

To generate cartilage-specific *Irf2bp2* knockout mice, female $Irf2bp2^{f/f}$ mice were crossed with the *Aggrecan-Cre* (*Acan-Cre*) strain to generate *Acan-Cre;* $Irf2bp2^{f/f}$ ($Irf2bp2^{Acan}$) mice, or with *Col2-CreERT2* mice to generate *Col2-CreERT2;* $Irf2bp2^{f/f}$ ($Irf2bp2^{Col2\text{-}ERT2}$) mice. $Irf2bp2^{Col2\text{-}ERT2}$ mice were treated with tamoxifen (100 mg/kg) at 10 weeks of age to induce Cre-mediated recombination. Tamoxifen (MCE, HY-13757) was first dissolved in sterile 100% ethanol, and then diluted with sterile corn oil (Beyotime, ST1177). Recombination was induced 2 weeks before Sham or DMM surgery by daily injection of tamoxifen for 3 days.

Acan-Cre and *Col2-CreERT2* mice were kindly provided by Prof. Weiguo Zou. All mice were on the C57BL/6J background and had undergone eight generations of backcrossing following standard procedures.

Mouse genotyping

Genomic DNA was extracted from mouse tissues with 50 mM NaOH at 100°C for 20 minutes, and then 1 M Tris-HCl (pH 7.5) was added. Mouse genotyping was performed by PCR. The genotyping primers were as follows:

Irf2bp2$^{-/-}$	IRF2BP2-WT-F	5’-GTCAACTACGAGGGAGCCGA-3’
	IRF2BP2-WT-R	5’-AACCGTTGACCAGAGGCAC-3’
	IRF2BP2-KO-F	5’-TCTTTGCTCTGGTGCCTCAGTTG-3’
	IRF2BP2-KO-R	5’-GTCTTGAGTTCCTGGGTCAGAAG-3’
Irf2bp2$^{f/f}$	IRF2BP2-flox-F	5’-AGACAGAGGATAGATGGAGACAGAGG-3’
	IRF2BP2-flox-R	5’-AGTCCTGAACTCAGCAAGCAGAC-3’
HA-Irf2bp2	HA-IRF2BP2-WT-F	5’-TGTGCGGACATGGCCGCG-3’
	HA-IRF2BP2-WT-R	5’-GCACCGTCTTAGGCCTGCTG-3’
	HA-IRF2BP2-F	5’-TATCCGTATGATGTGCCGGATTATGC-3’
	HA-IRF2BP2-R	5’-AGTTTGGAGAACCCGTTGGG-3’
Acan-Cre	Universal Cre-1-F	5’-GTATTGAATTGAAGCACCTTTGTTTGG-3’
	Universal Cre-1-R	5’-CTGCCCAAGGCTCCCCCAG-3’
	Universal Cre-2-F	5’-CCTGGAAAATGCTTCTGTCCG-3’
	Universal Cre-2-R	5’-CAGGGTGTTATAAGCAATCCC-3’

Col2-CreERT2	Col2-Cre-F	5’-CGATGCAACGAGTGATGAGG-3’
	Col2-Cre-R	5’-CGCATAACCAGTGAAACAGC-3’
	ERT-F	5’-GGGTGCCAGGCTTTGTGGATTT-3’
	ERT-R	5’-TTCAGGGTGCTGGACAGAAATG-3’

Meniscal injury scoring

We scored all menisci from the DMM model using a standardized, published histopathology scoring system for mouse meniscal degeneration (DOI: 10.1007/s11999-009-0921-8). Two independent observers, blinded to genotype and treatment, scored each sample**.**

Measurements of thermal hyperalgesia

Paw withdrawal latency was assessed according to established protocols (DOI: 10.1038/s41421-022-00382-6).

Intra-articular injections

AAV vectors were constructed and packaged by Hanbio Biotechnology (Shanghai, China). All intra-articular injections were performed in a volume of 6 μL per knee joint. The detailed information was as follows:

AAV2/9-FOSL2	1×10^{13} vg/mL	Single injection
AAV2/9-Cre	1×10^{13} vg/mL	Single injection
AAV2/9-sh-IRF2BP2	1×10^{13} vg/mL	Single injection

IRF2BP2-peptide	4 mg/mL	Once weekly for 6 weeks
FOSL2-peptide	4 mg/mL	Once weekly for 6 weeks
15S-HPETE	10 μg/mL	Once weekly for 6 weeks
T-5224	10 mM	Once weekly for 6 weeks
Ad-iCre	1×10^{11} vg/mL	Once weekly for 6 weeks

Antibodies

The antibodies used in IF, IHC, and Western blot were as follows:

Rabbit anti-HA (IF 1:200)	CST, 3724
Rabbit anti-SOX9 (IF 1:1000, IB 1:1000)	Millipore, AB5535
Rabbit anti-Ki67 (IF 1:1000)	Abcam, ab15580
Rabbit anti-IRF2BP2 (IF 1:200)	Abcam, ab114997
Rabbit anti-IRF2BP2 (IF 1:200, IB 1:1000)	Sigma, HPA027815
Rabbit anti-COL10A1 (IF 1:200)	Abcam, ab49945
Rabbit anti-COL2A1 (IF 1:200, IHC 1:200)	HuaAn Bio, HA722733
Rabbit anti-COL1A1 (IHC 1:200)	HuaAn Bio, HA722517
Rabbit anti-FOSL2 (IF 1:200, IHC 1:200, IB 1:1000)	Sigma, HPA004817
Rabbit anti-MMP3 (IF 1:200, IB 1:1000)	HuaAn Bio, ET1705-98
Rabbit anti-c-Jun (IHC 1:200, IB 1:1000)	Abclonal, A0246
Rabbit anti-COL2A1 (IB 1:1000)	Abclonal, A1560
Rabbit anti-MMP13 (IB 1:1000)	Abcam, ab39012
Rabbit anti-FLAG (IB 1:1000)	Sigma, F7425

Rabbit anti-MYC (IB 1:1000)	Sigma, C3956
Rabbit anti-HA (IB 1:1000)	Invitrogen, 71-5500
Rabbit anti-YAP (IB 1:1000)	Abclonal, A1002
Rabbit anti-p-YAP (S127) (IB 1:1000)	CST, 4911S
Rabbit anti-CYR61 (IB 1:1000)	Abclonal, A1111
Rabbit anti-ANKRD1 (IB 1:1000)	Abclonal, A6192
Rabbit anti-β-Actin (IB 1:10000)	Abclonal, AC026
Mouse anti-GAPDH (IB 1:10000)	Abclonal, AC033

Immunoprecipitation and immunoblotting

For the immunoprecipitation assay, HEK-293T cells were transiently transfected with the indicated plasmids. After 48 h, cells were harvested and lysed. The lysates were incubated with the indicated antibodies at 4°C overnight. Protein A/G beads (Santa Cruz) were then added for 1 hour, followed by washing with IP buffer for three times. Subsequently, the samples were prepared in SDS loading buffer and subjected to SDS–polyacrylamide gel electrophoresis (PAGE), followed by immunoblotting with the indicated antibodies. All Western blot experiments were independently repeated at least twice.

Ubiquitination assay

ATDC5 cells were transfected with the indicated plasmids and treated with MG132 (50 μM) for 4 hours before harvesting. Cells were then lysed with denaturing buffer

(100 mM Tris-Cl, pH 7.5, 10% SDS, 0.1 M DTT) and boiled at 100°C for 10 min. The lysates were then diluted to 1 mL with NP40 buffer. Immunoprecipitation and Western blotting were then carried out.

BioID

The MYC-IRF2BP2-BirA mass spectrometry (MS) screen was performed as described (DOI: 10.1038/s44318-024-00257-4).

Alcian blue staining

The Alcian blue staining was performed according to established protocols (DOI: 10.1038/s41421-022-00382-6).

shRNA-mediated knockdown of IRF2BP2

For stable knockdown of IRF2BP2 in ATDC5 cells, short hairpin RNA (shRNA) targeting mouse *Irf2bp2* was cloned into the pLKO.1-puro lentiviral vector. The targeting sequence was: 5'-TTGCTCCAGACAAAGCATTAA-3'.

Alizarin red and Alcian blue double staining

Whole-mount skeletal preparation was performed with Alcian blue and Alizarin red to evaluate cartilage and bone development at P1. After fixation in 95% ethanol for 48 hours, specimens were immersed in 0.015% Alcian blue (Sangon Biotech, A600298) dissolved in 80% ethanol and 20% acetic acid for 24 hours at room temperature

(RT). The specimens were then transferred to 0.005% Alizarin red (Sigma, A5533) in 1% KOH for 12 hours at RT. The stained specimens were then gradually dehydrated in graded glycerol/KOH solutions (20%, 50%, and 80% glycerol in 1% KOH; 24 hours for each step) until optimal tissue transparency was achieved.

Safranin O (S.O.) and Fast Green staining

Mouse knee joints were fixed in 4% paraformaldehyde (PFA), dehydrated through a graded ethanol series, cleared in xylene, and embedded in paraffin. Serial sections (8 μm) were stained with 0.2% Fast Green (10 min) and 0.1% Safranin O (30 min). The severity of osteoarthritis was graded using the OARSI scoring system based on Safranin O/Fast Green staining.

Histology, Immunofluorescence, and Immunohistochemistry

Tissue samples were fixed in 4% paraformaldehyde, decalcified in 0.5 M EDTA (pH 7.5), dehydrated in 30% sucrose, and embedded in OCT compound. Serial frozen sections (10 μm) were prepared for H&E and IF staining. H&E staining was performed following established protocols. For IF staining, sections were blocked with 5% BSA and 0.1% Triton X-100, incubated with primary antibodies overnight at 4°C, followed by incubation with Alexa Fluor secondary antibodies and DAPI. Imaging was performed using a Leica SP8 confocal microscope. For IHC, paraffin-embedded sections were deparaffinized, rehydrated, and subjected to antigen retrieval in sodium citrate buffer (pH 6.0). After blocking, sections were incubated

with primary antibodies overnight, followed by incubation with HRP-conjugated secondary antibody. Signals were developed using DAB buffer and counterstaining was performed with hematoxylin.

Primary articular chondrocyte culture and differentiation

Primary articular chondrocytes were isolated from the articular cartilage of knee joints of newborn mice. Cartilage tissues were minced into fragments and digested with 0.1% collagenase type II (Gibco, 17101015) in DMEM/F12 medium at 37°C for 12 hours. Primary chondrocytes were harvested by centrifugation and cultured at a density of 5×10^3 cells per mL at 37 °C with 5% CO_2. DMEM/F12 medium with 10% FBS and 1% penicillin–streptomycin was used to culture the primary chondrocytes. For chondrogenic differentiation, the cells were cultured in differentiation medium which contained insulin-transferrin-selenium (ITS) (Sigma, I3146), 10 ng/mL TGF-β3 (PeproTech, 100-36E), 100 nM dexamethasone (Sigma, D4902), 50 µg/mL vitamin C (Sigma, A92902), 1 mM sodium pyruvate (Sigma, P2256), and 40 µg/mL proline (Sigma, P0380) in DMEM/F12 for the indicated number of days.

RNA extraction, reverse transcription, and real-time PCR

Total RNA was extracted from samples using TRIzol reagent (Vazyme, R401-01). RNA was reverse-transcribed using HiScript III RT SuperMix for qPCR (Vazyme, R323-01). Real-time PCR was performed with AceQ Universal SYBR qPCR Master

Mix (Vazyme, Q511-02), with GAPDH serving as the internal control. The primer sequences used are listed as follows:

Mouse-GAPDH-F	5'-CTCCCACTCTTCCACCTTCG-3'
Mouse-GAPDH-R	5'-TAGGGCCTCTCTTGCTCAGT-3'
Mouse-IRF2BP2-F	5'-AGTTCTGTTTCCCTTGCTCC-3'
Mouse-IRF2BP2-R	5'-TCACTTTCACATCTCCGG-3'
Mouse-SOX9-F	5'-CAGCCCCTTCAACCTTCCTC-3'
Mouse-SOX9-R	5'-TGATGGTCAGCGTAGTCGTATT-3'
Mouse-COL2A1-F	5'-CCTCCGTCTACTGTCCACTGA-3'
Mouse-COL2A1-R	5'-ATTGGAGCCCTGGATGAGCA-3'
Mouse-MMP3-F	5'-ACATGGAGACTTTGTCCCTTTTG-3'
Mouse-MMP3-R	5'-TTGGCTGAGTGGTAGAGTCCC-3'
Mouse-MMP13-F	5'-CTTCTTCTTGTTGAGCTGGACTC-3'
Mouse-MMP13-R	5'-CTGTGGAGGTCACTGTAGACT-3'
Mouse-FOSL2-F	5'-CACGCCGAGTCCTACTCCA-3'
Mouse-FOSL2-R	5'-GTGGGCTGTACCATCCACTG-3'
Human-GAPDH-F	5'-CAGGTGGTCTCCTCTGACTT-3'
Human-GAPDH-R	5'-CCAAATTCGTTGTCATACCA-3'
Human-IRF2BP2-F	5'-CCCATGACTCCTACATCCTCTT-3'
Human-IRF2BP2-R	5'-GAGGGCGGACTGTTGCTATTC-3'
Human-FOSL2-F	5'-CAGAAATTCCGGGTAGATATGCC-3'
Human-FOSL2-R	5'-GGTATGGGTTGGACATGGAGG-3'

Human-MMP3-F	5’-AGTCTTCCAATCCTACTGTTGCT-3’
Human-MMP3-R	5’-TCCCCGTCACCTCCAATCC-3’
Human-MMP13-F	5’-ACTGAGAGGCTCCGAGAAATG-3’
Human-MMP13-R	5’-GAACCCCGCATCTTGGCTT-3’
Human-COL10A1-F	5’-ATGCTGCCACAAATACCCTTT-3’
Human-COL10A1-R	5’-GGTAGTGGGCCTTTTATGCCT-3’

Chromatin immunoprecipitation-qPCR (ChIP-qPCR)

Cells were cross-linked with 1% formaldehyde, quenched with glycine, and lysed. Chromatin was sheared into 200-500 bp fragments by sonication. Rabbit IgG and antibodies against the target proteins were used for immunoprecipitation overnight. Protein A/G beads were added to enrich protein-DNA complexes. Purified DNA was used for qPCR to quantify protein binding. The primer sequences used are listed as follows:

Human-SOX9-1-F	5’-GGCGTTGAGTCACCAAAACAT-3’
Human-SOX9-1-R	5’-CAGCCGCTTCTTAATGCGTAG-3’
Human-SOX9-2-F	5’-GGCGAACCCGAGCATGT-3’
Human-SOX9-2-R	5’-TTTTGGTGACTCAACGCCCG-3’
Mouse-SOX9-F	5’-TGCAAACTCCAGTGGCGAG-3’
Mouse-SOX9-R	5’-CTCTGACGCCCCTCGTAGTC-3’

Chemicals, enzymes and other kits

Safranin O	Sigma, S2255
Fast Green	Sigma, F7252
Paraformaldehyde (PFA)	Sigma, 158127
DAPI	Sigma, D9542
H&E Kit	Beyotime, C0105M
IHC Kit	Vazyme, HC-301-02
Cycloheximide (CHX)	MCE, HY-12320
T-5224	MCE, HY-12270
15S-HPETE	MCE, HY-113438
Anti-FLAG magnetic beads	Selleck, B26101
Anti-MYC magnetic beads	Selleck, B26201
Anti-HA magnetic beads	Selleck, B26301
