# Supplementary material for: IRF2BP2-FOSL2 interaction regulates SOX9-mediated cartilage regeneration in osteoarthritis models
Source: J Clin Invest. 2026 Oct 15;136(20):e200039. doi: 10.1172/JCI200039 (PMC13668630; doi:10.1172/JCI200039)

Full unedited blot for Figure 2A

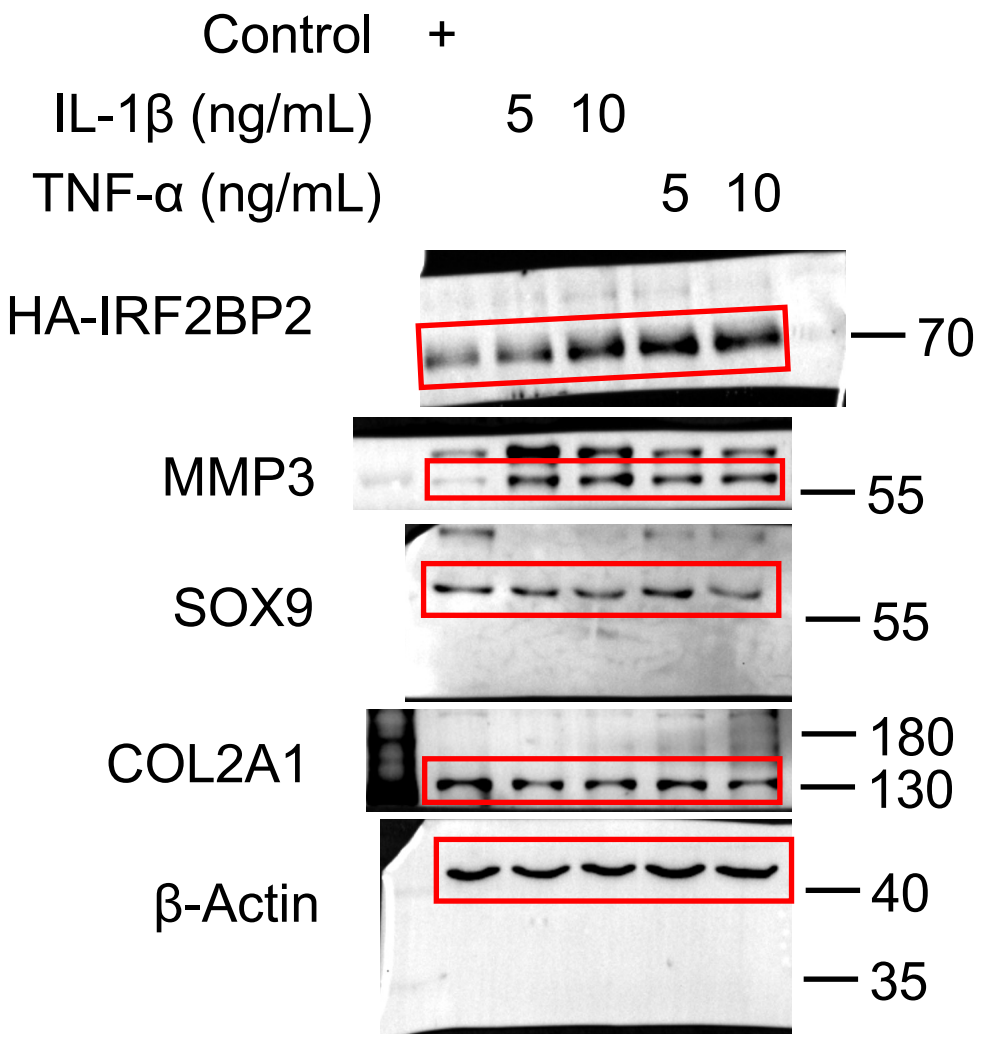

Full unedited blot for Figure 4A

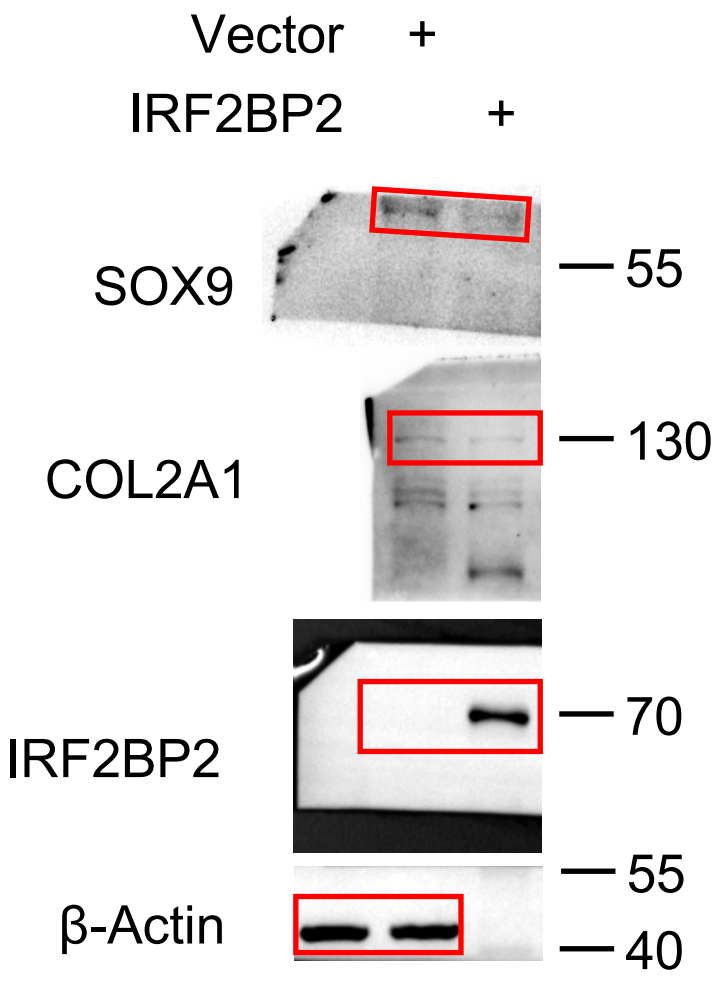

Full unedited blot for Figure 4B

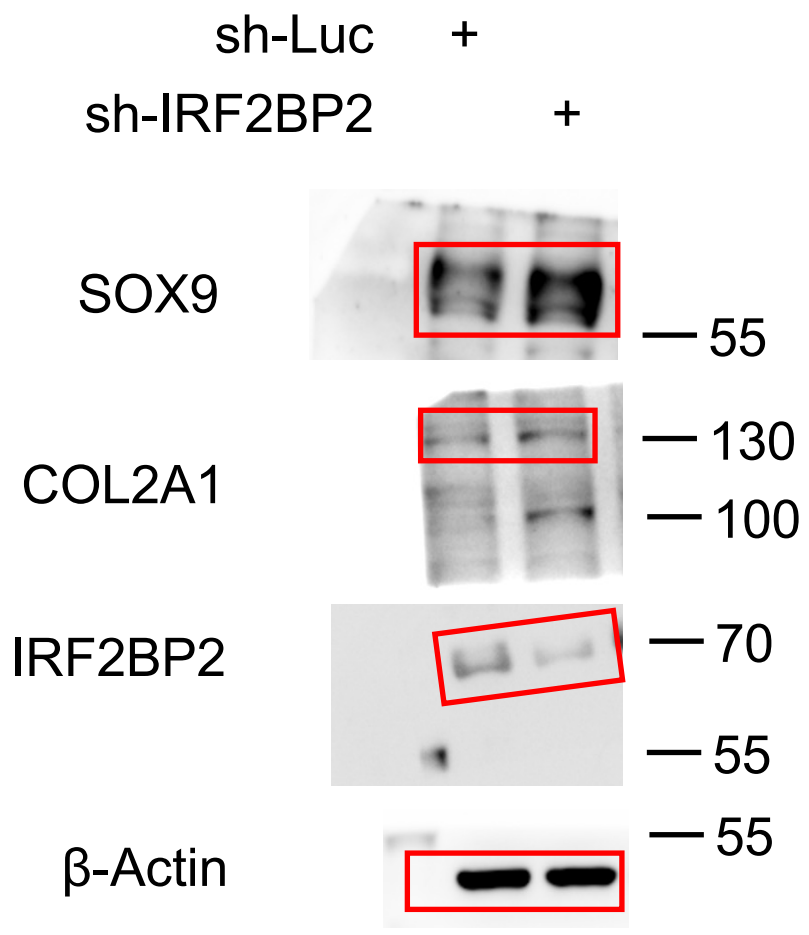

Full unedited blot for Figure 4E

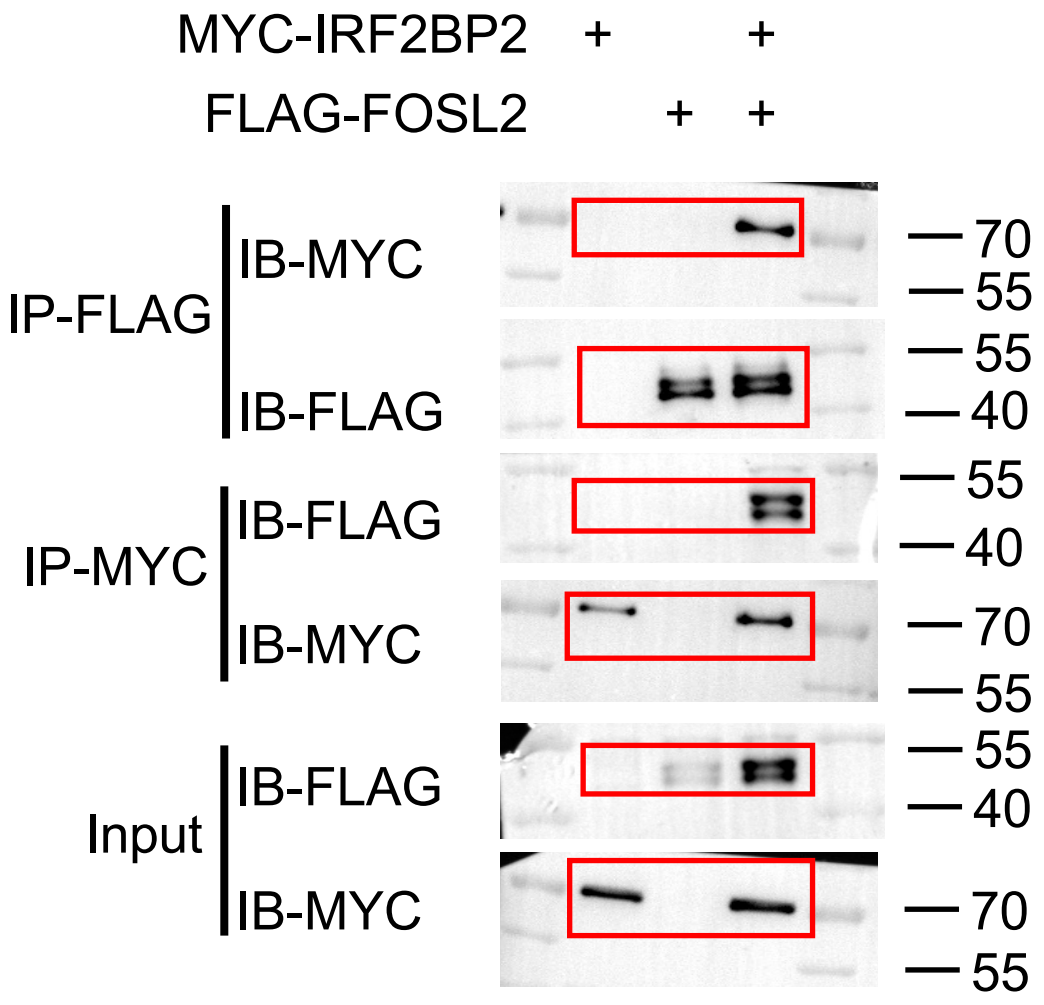

Full unedited blot for Figure 4G

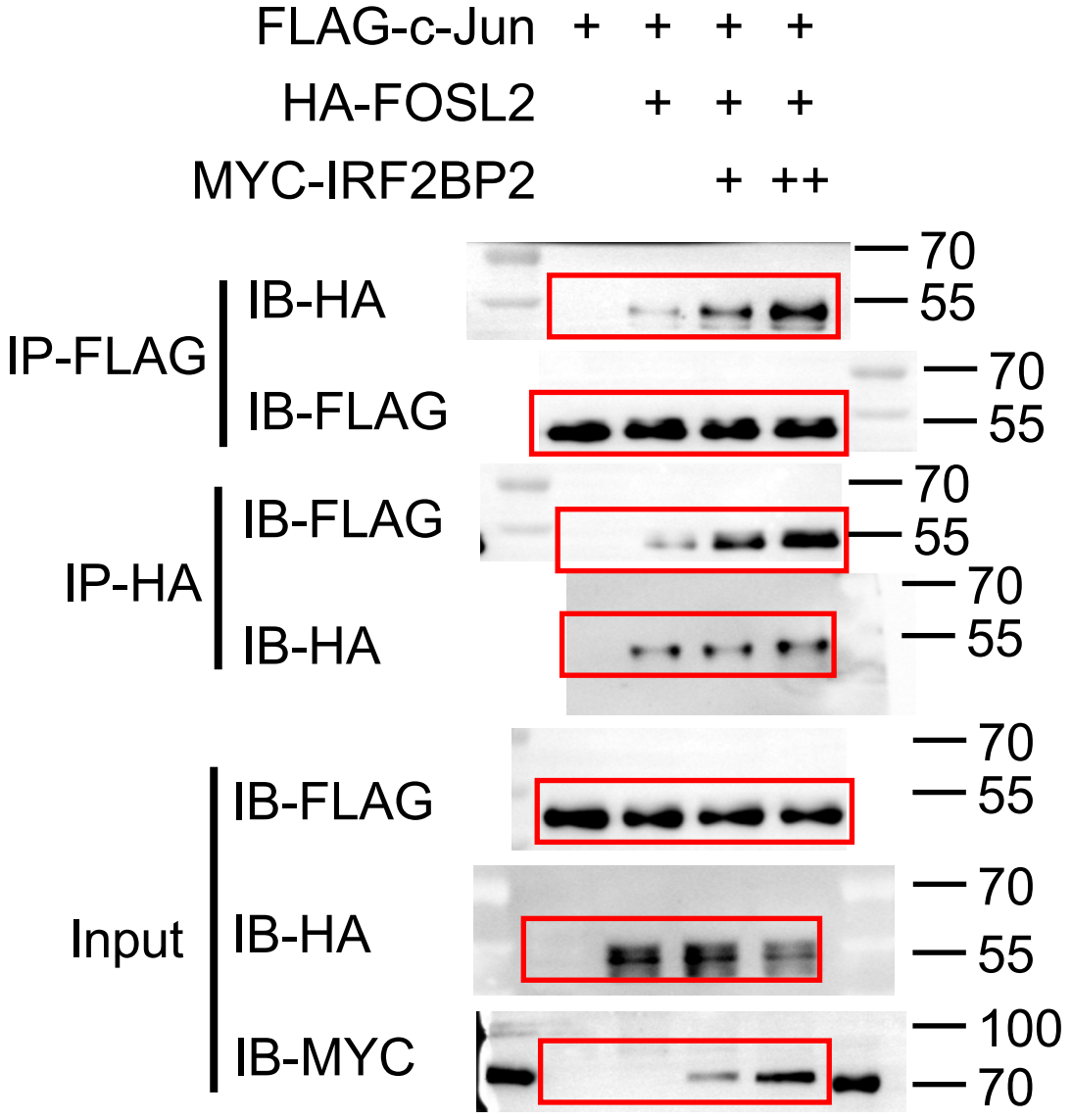

Full unedited blot for Figure 4I

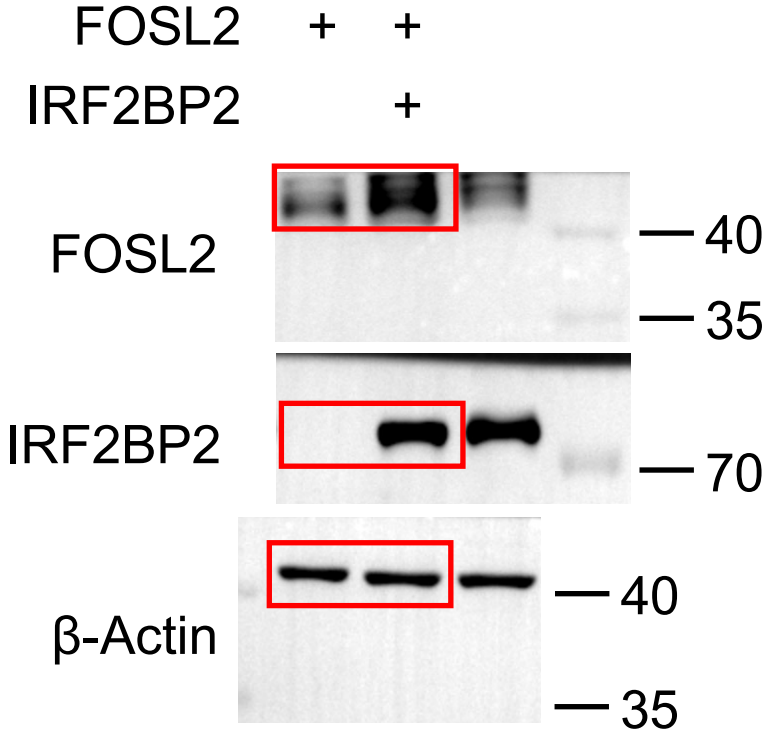

Full unedited blot for Figure 4J

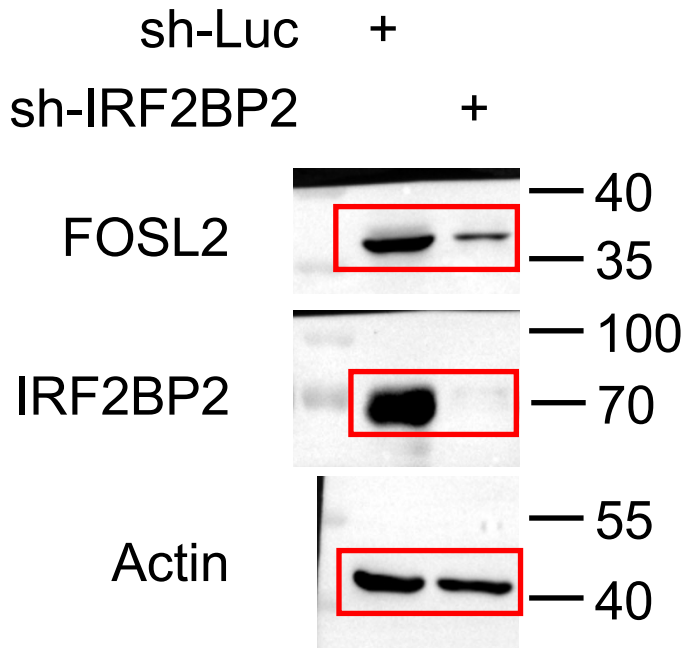

Full unedited blot for Figure 4M

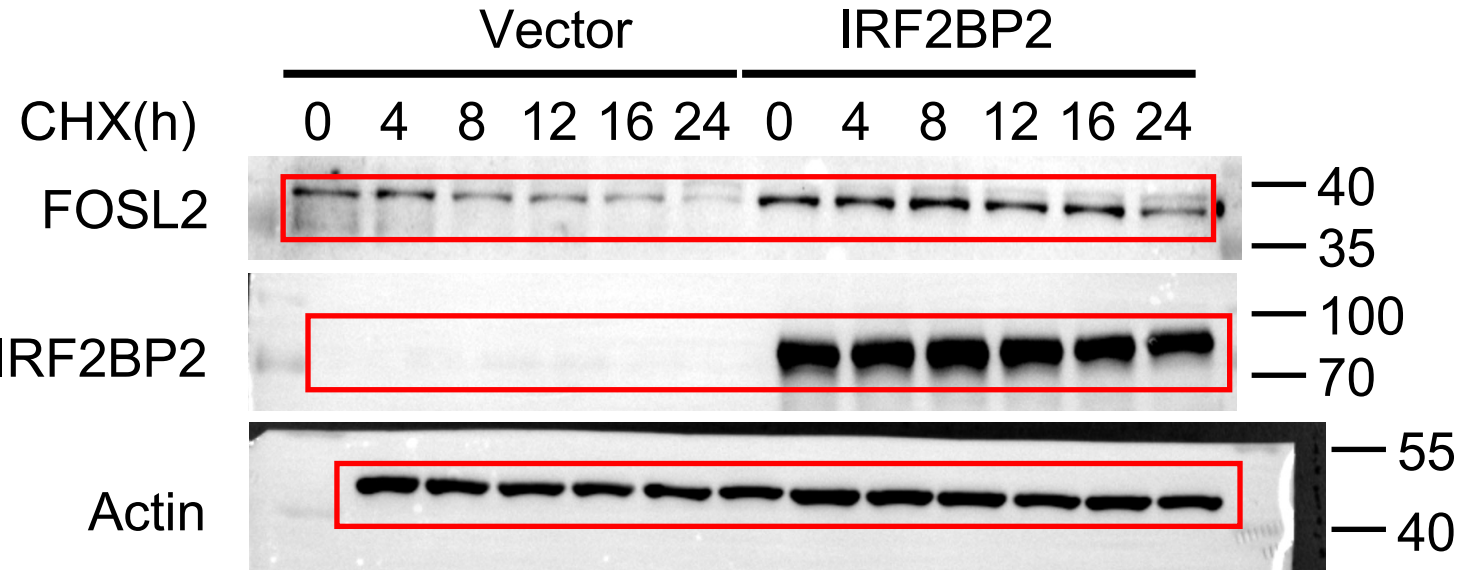

Full unedited blot for Figure 4O

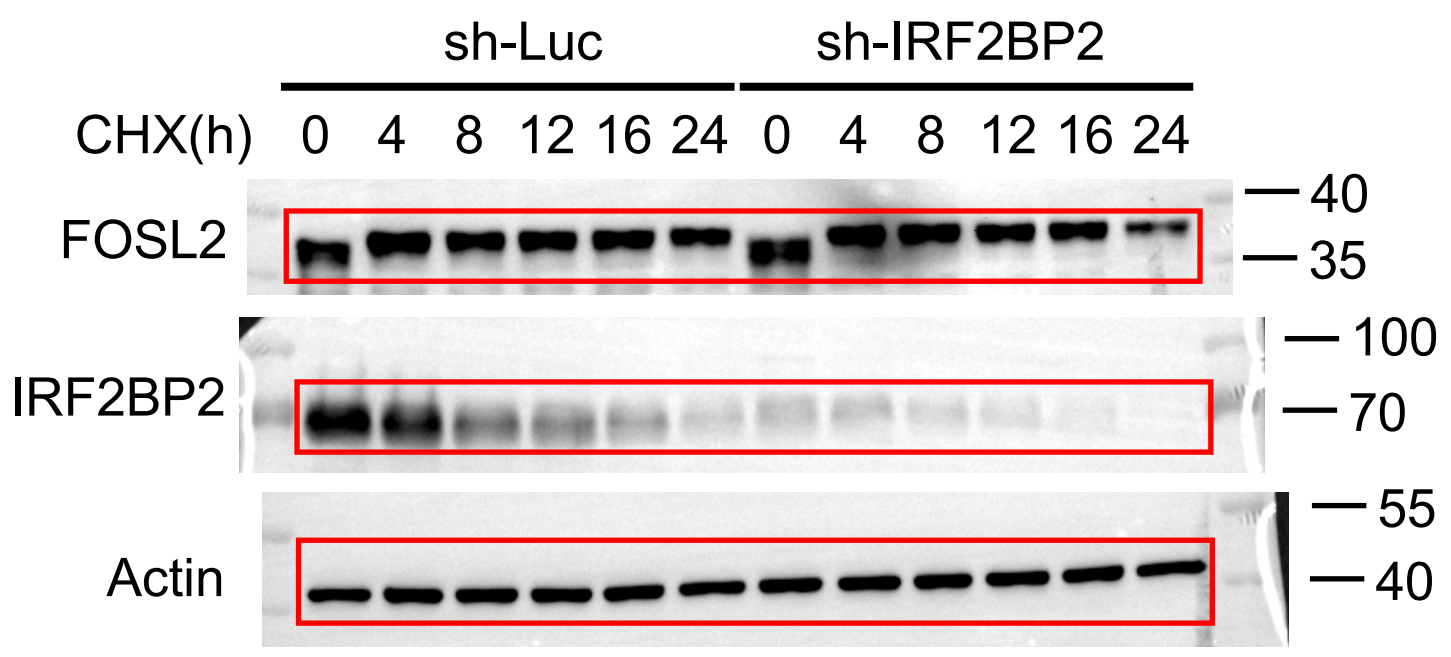

Full unedited blot for Figure 5B

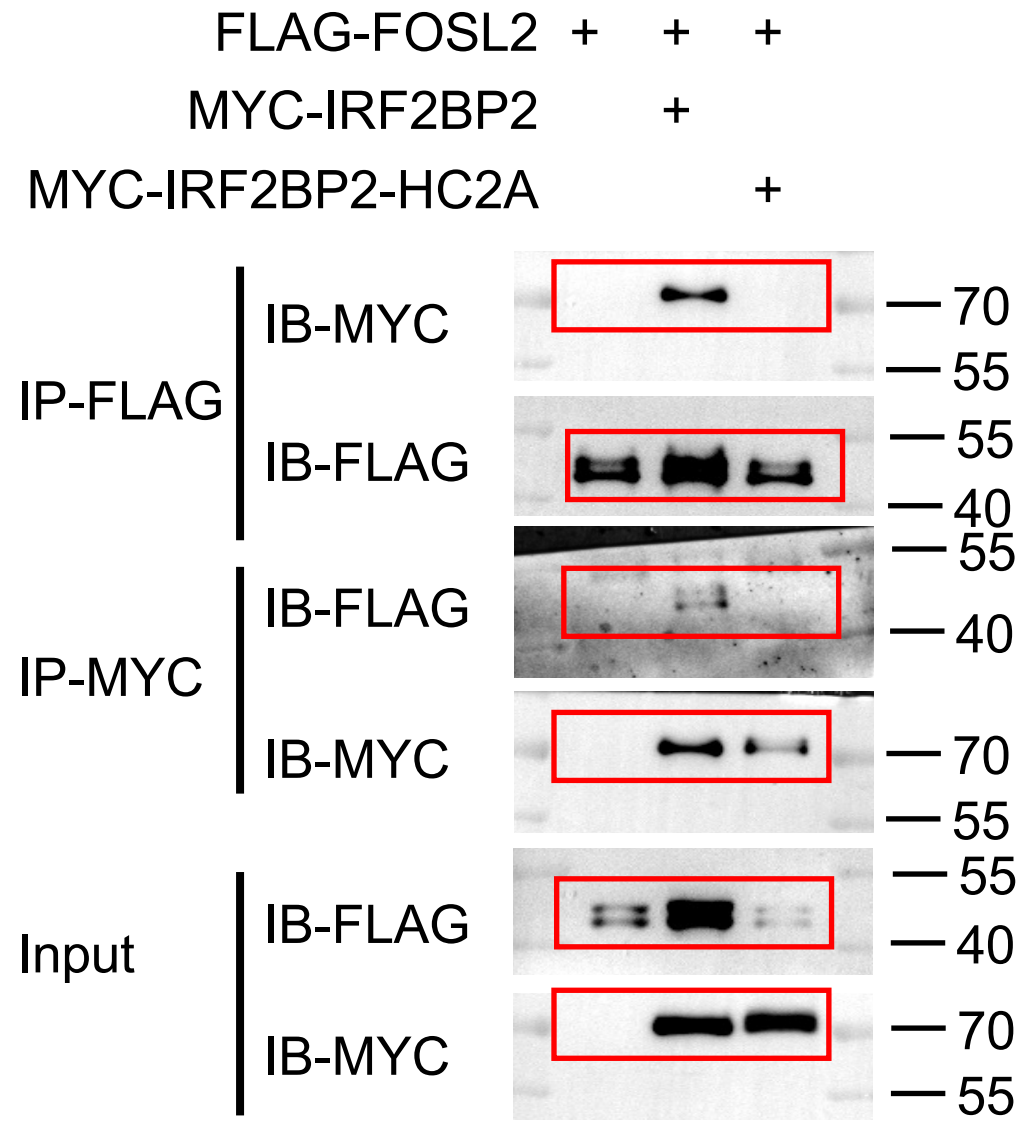

Full unedited blot for Figure 5C

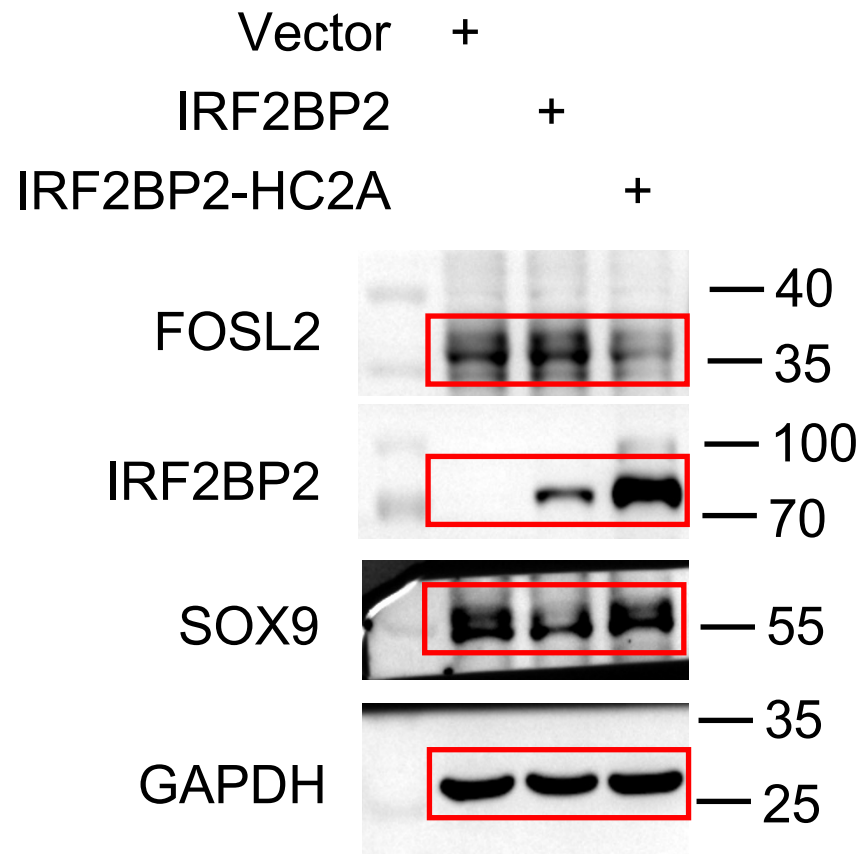

Full unedited blot for Figure 5F

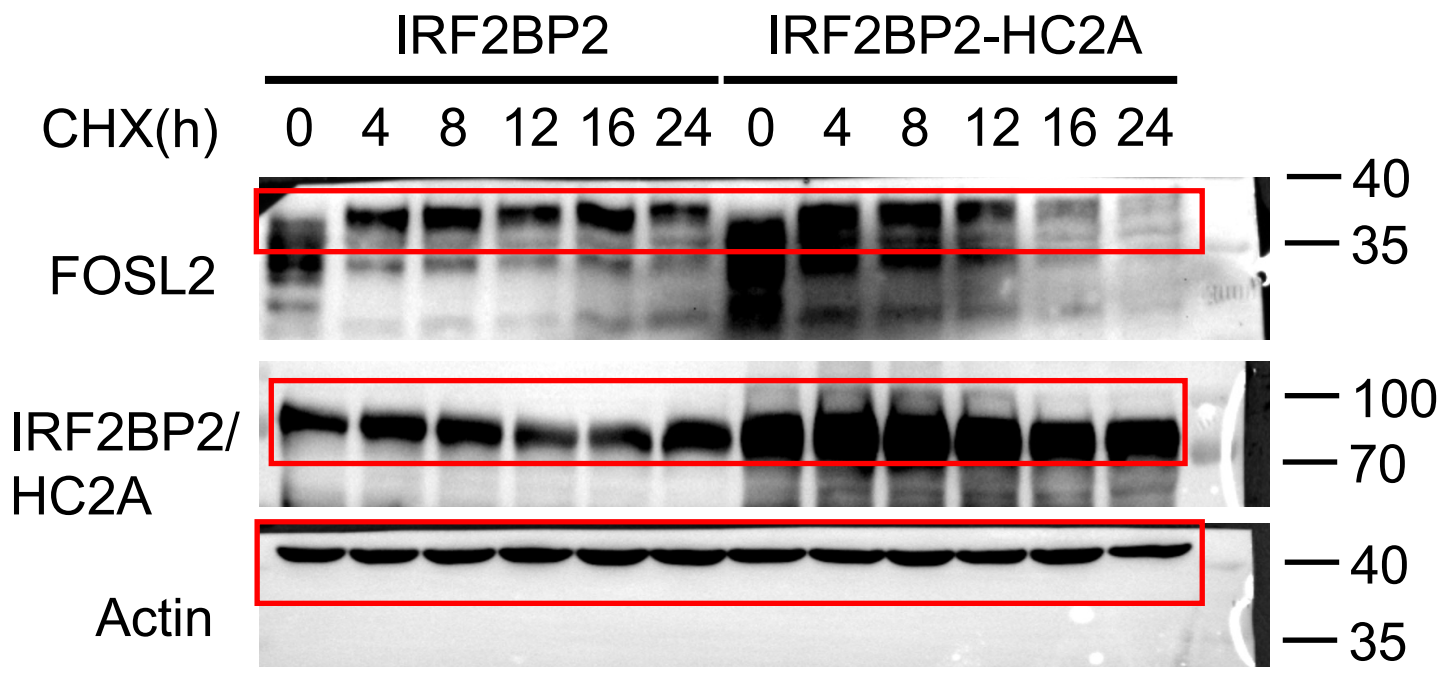

Full unedited blot for Figure 7A

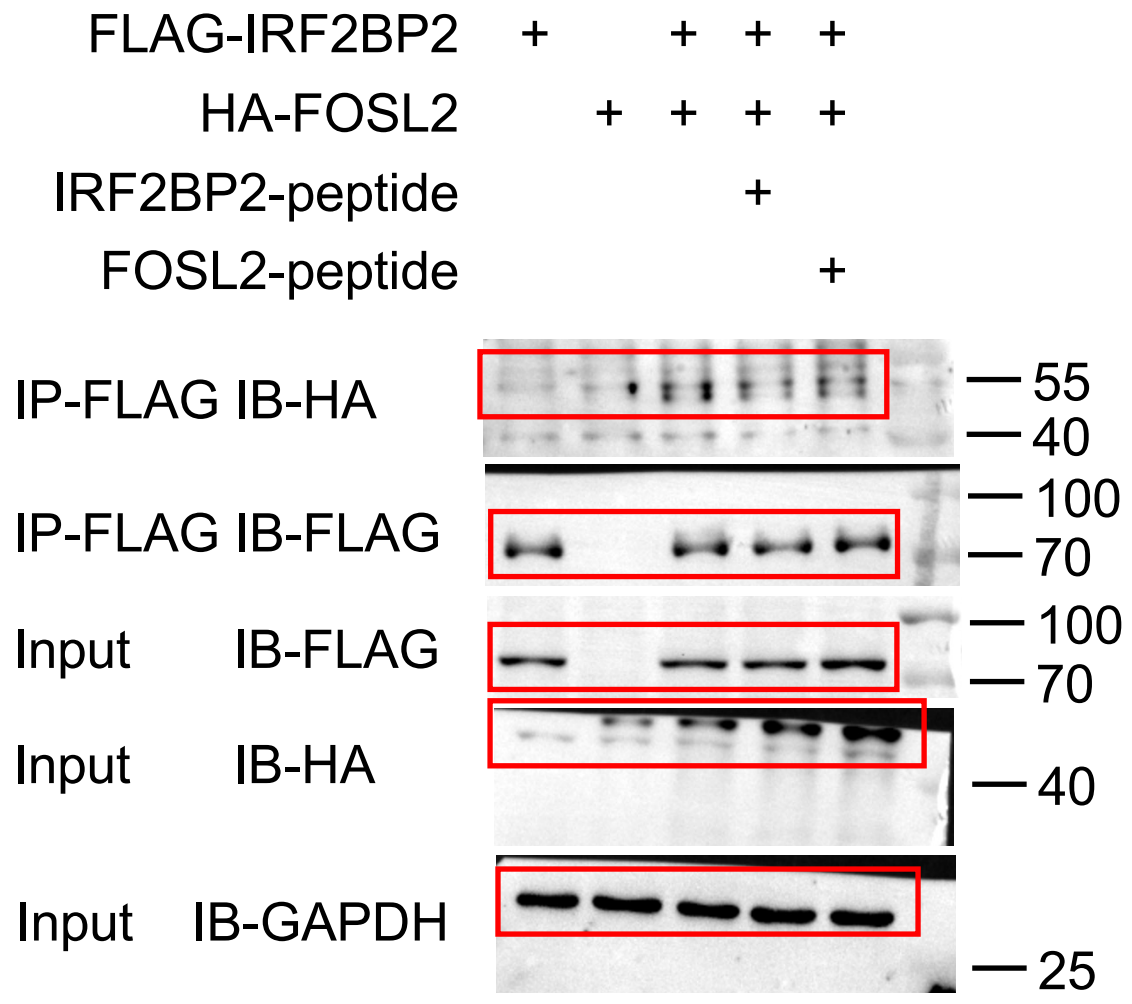

Full unedited blot for Figure 7B

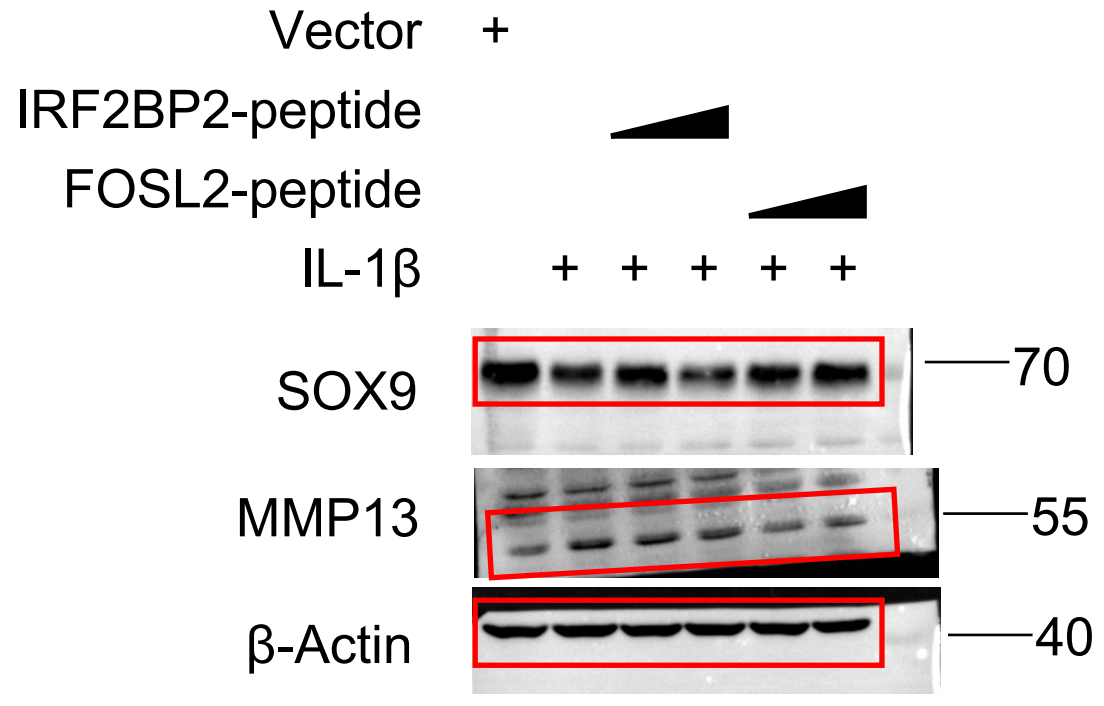

Full unedited blot for Figure 8A

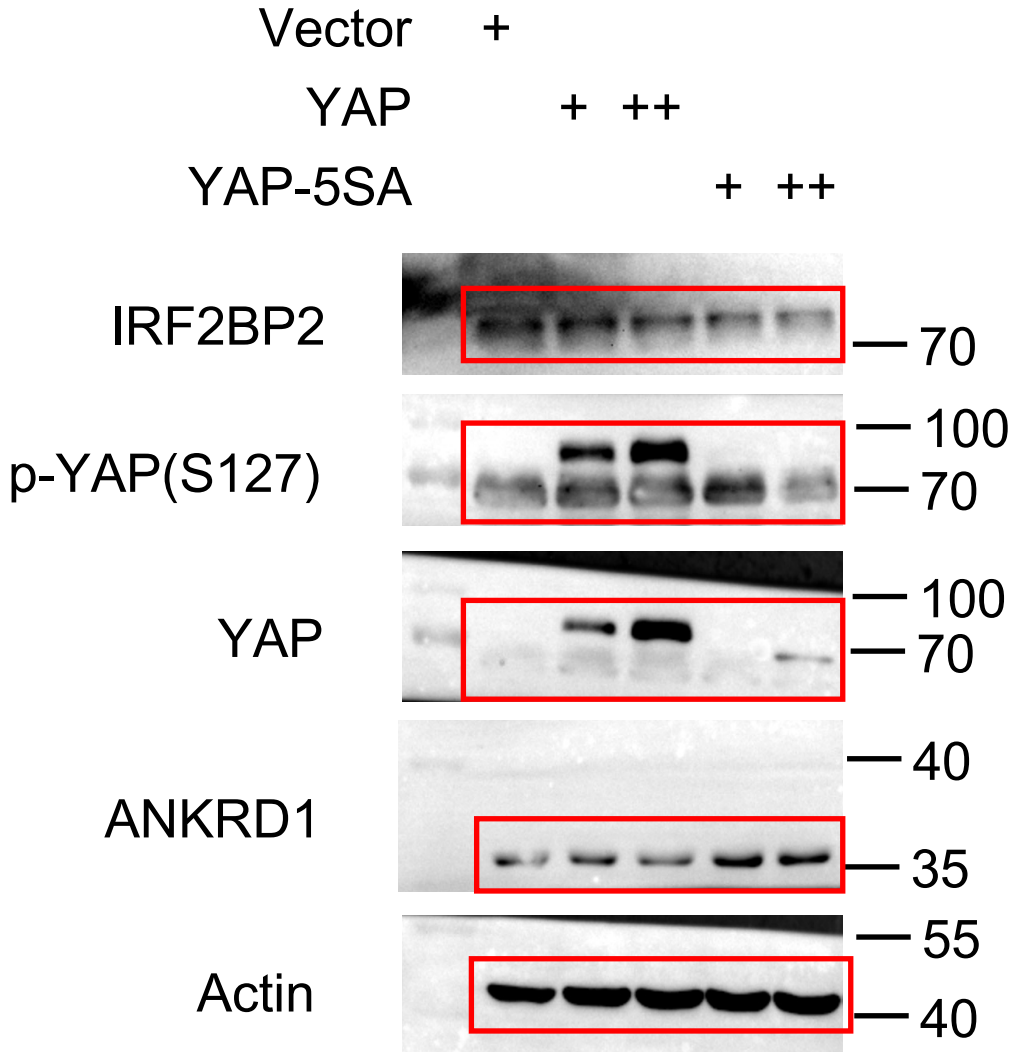

Full unedited blot for Figure 8C

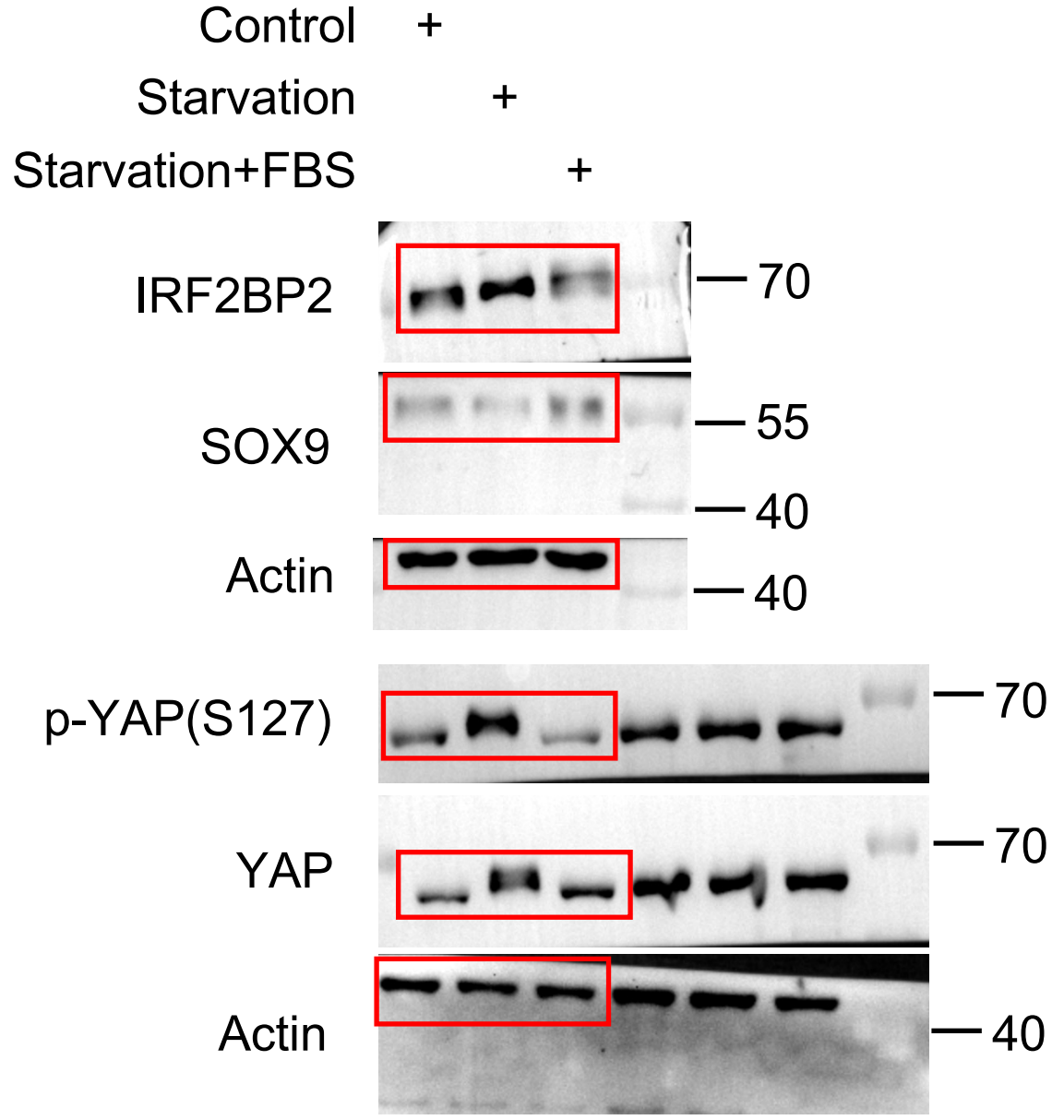

Full unedited blot for Figure 8D

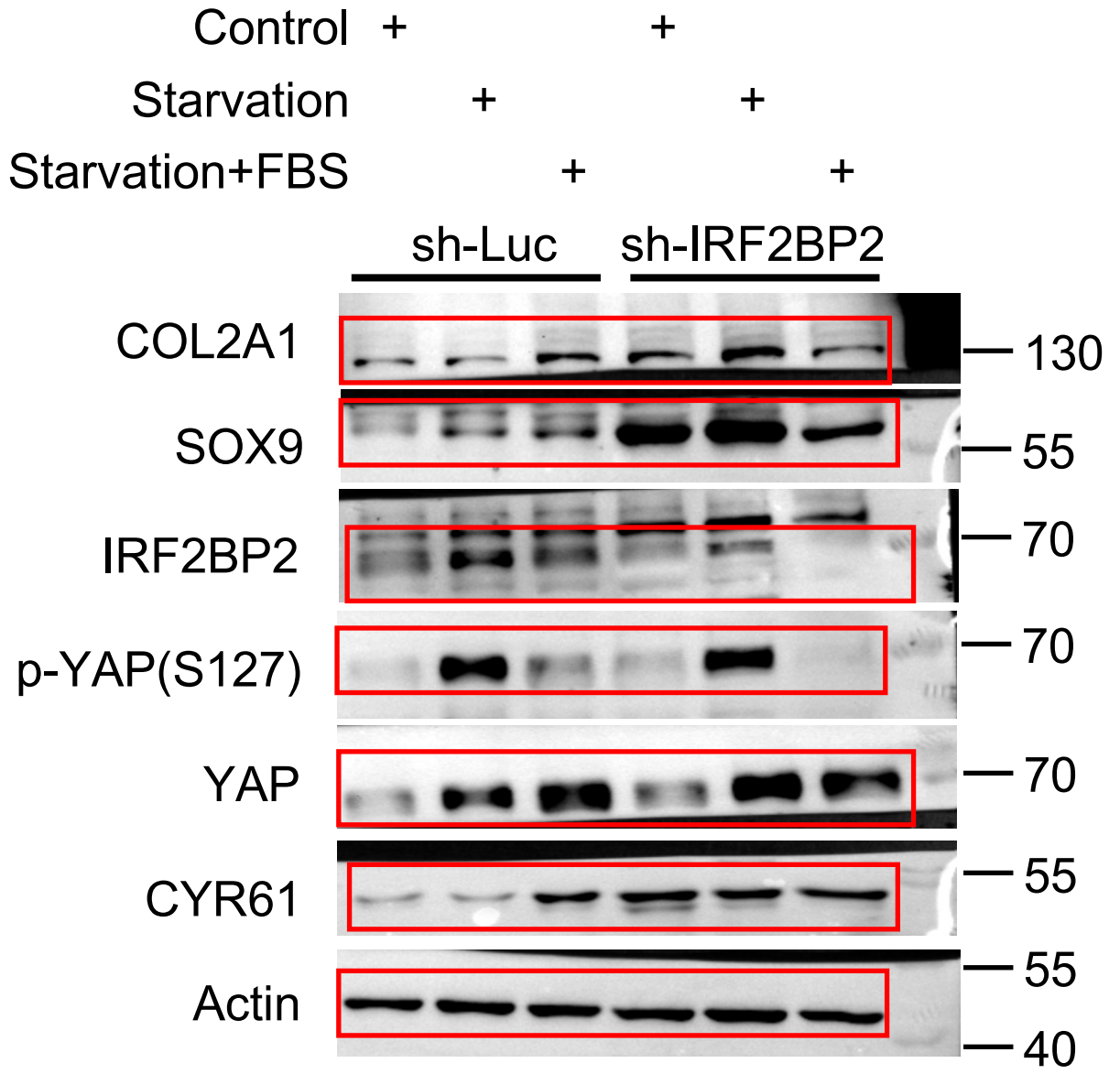

Full unedited blot for Figure 8E

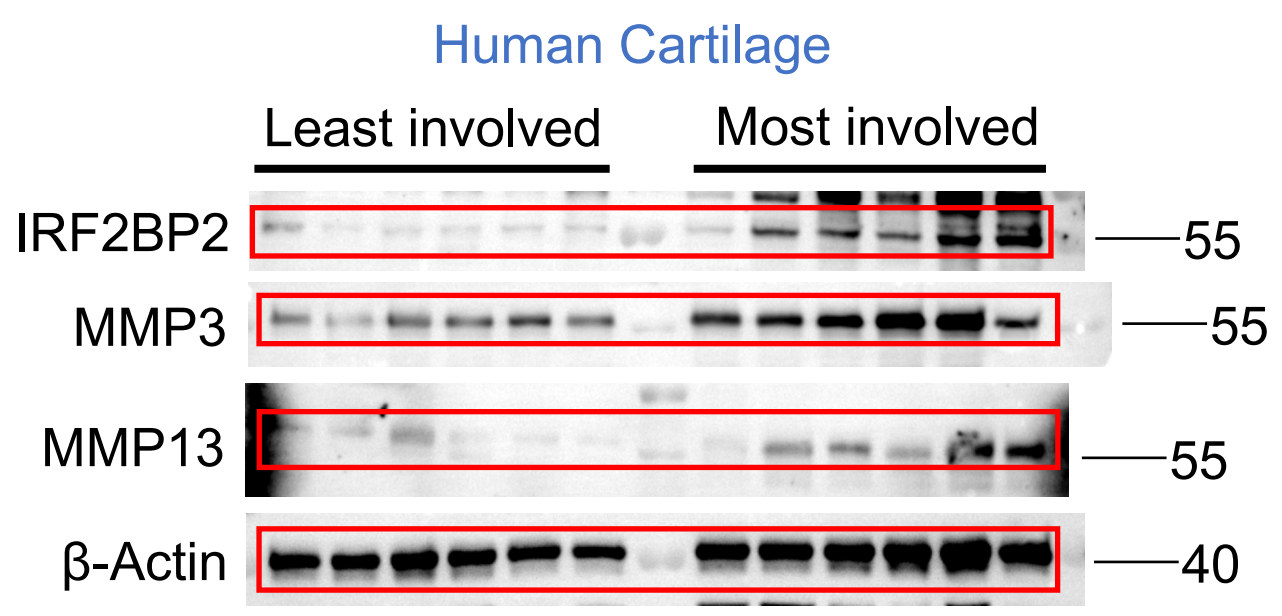

Full unedited blot for Figure S3N

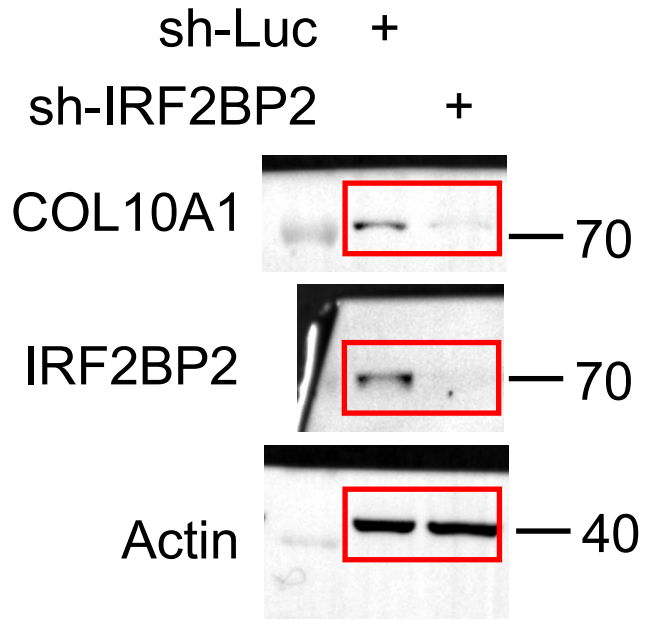

Full unedited blot for Figure S1H

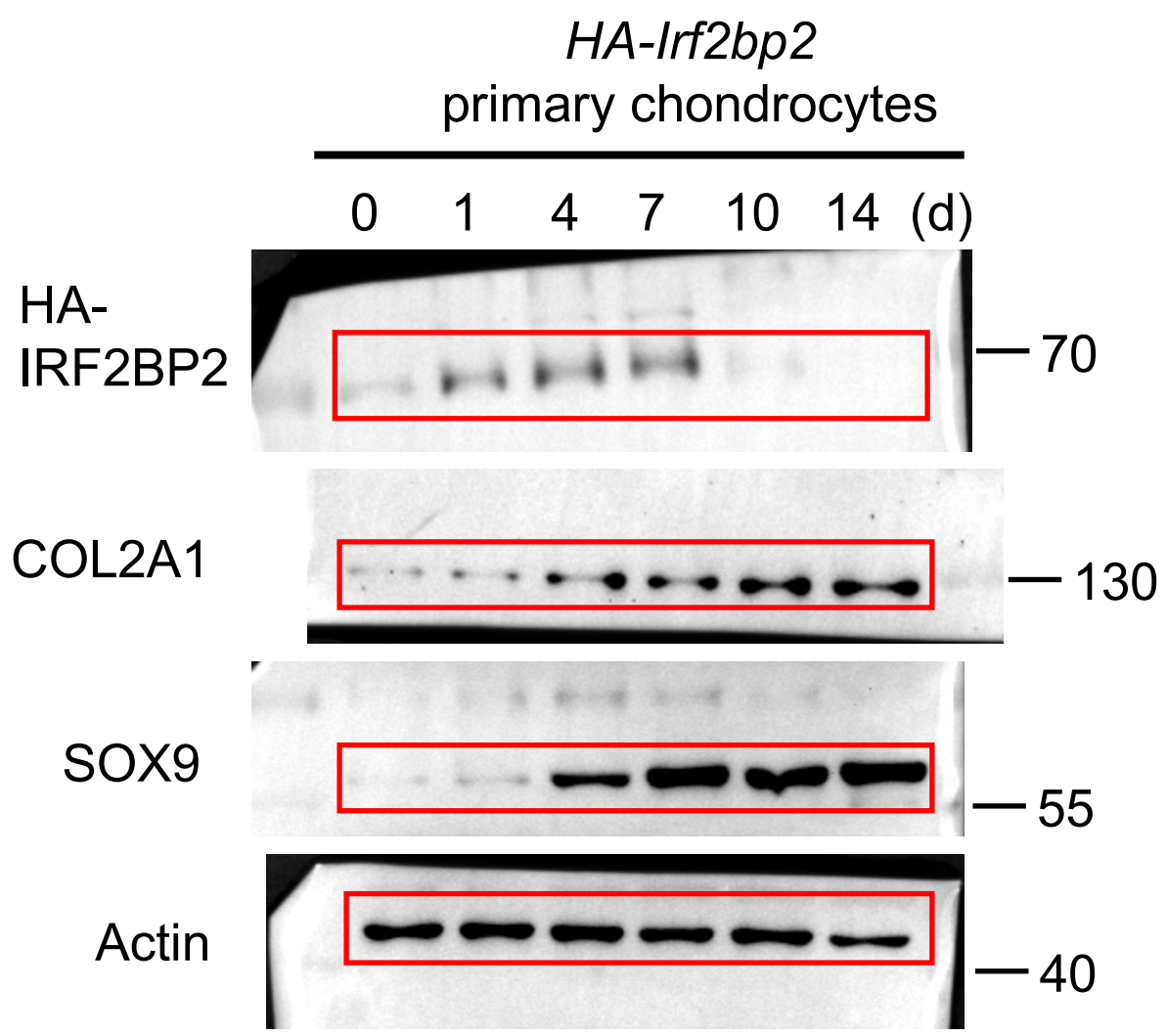

Full unedited blot for Figure S6C

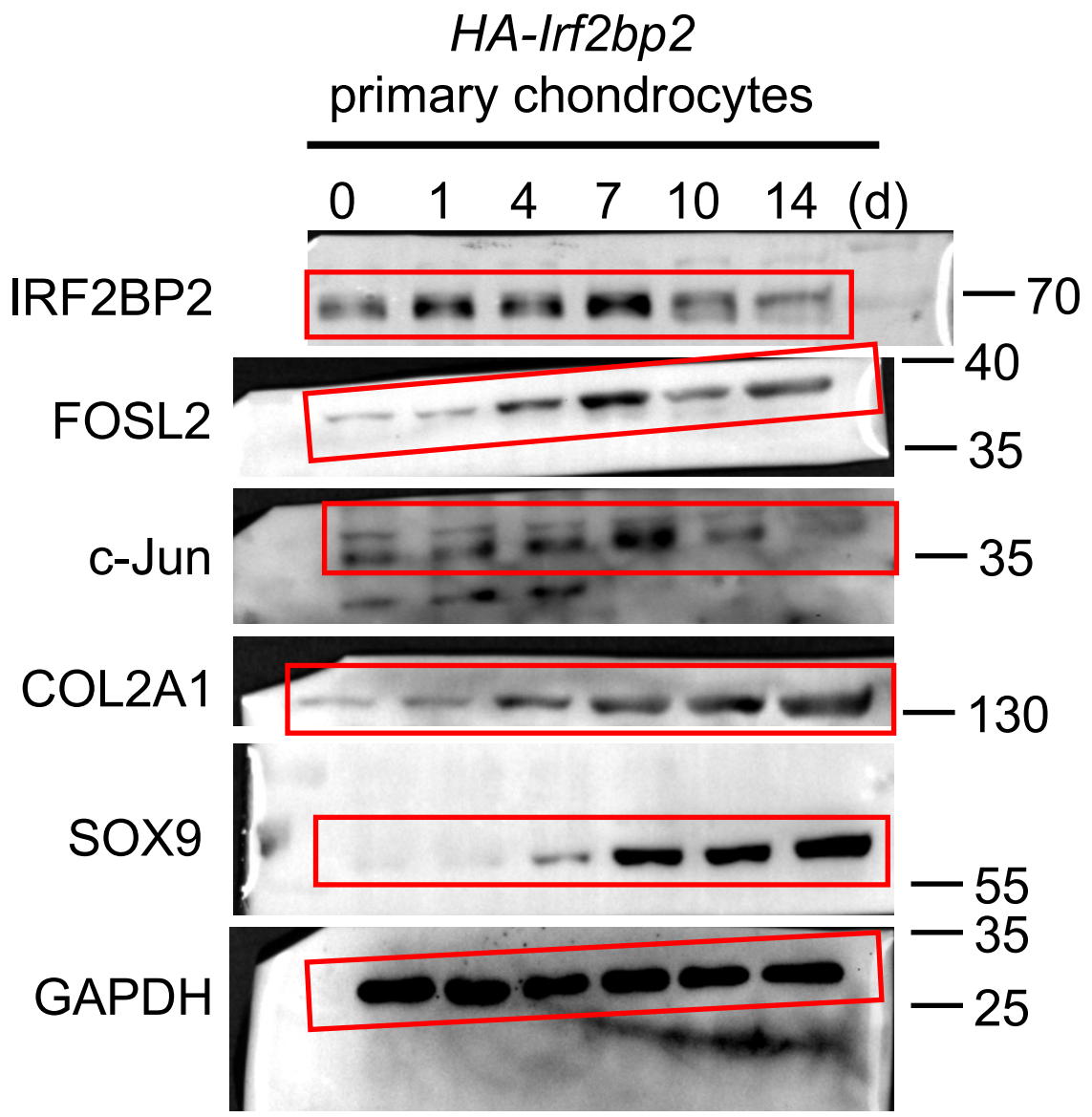

Full unedited blot for Figure S6J

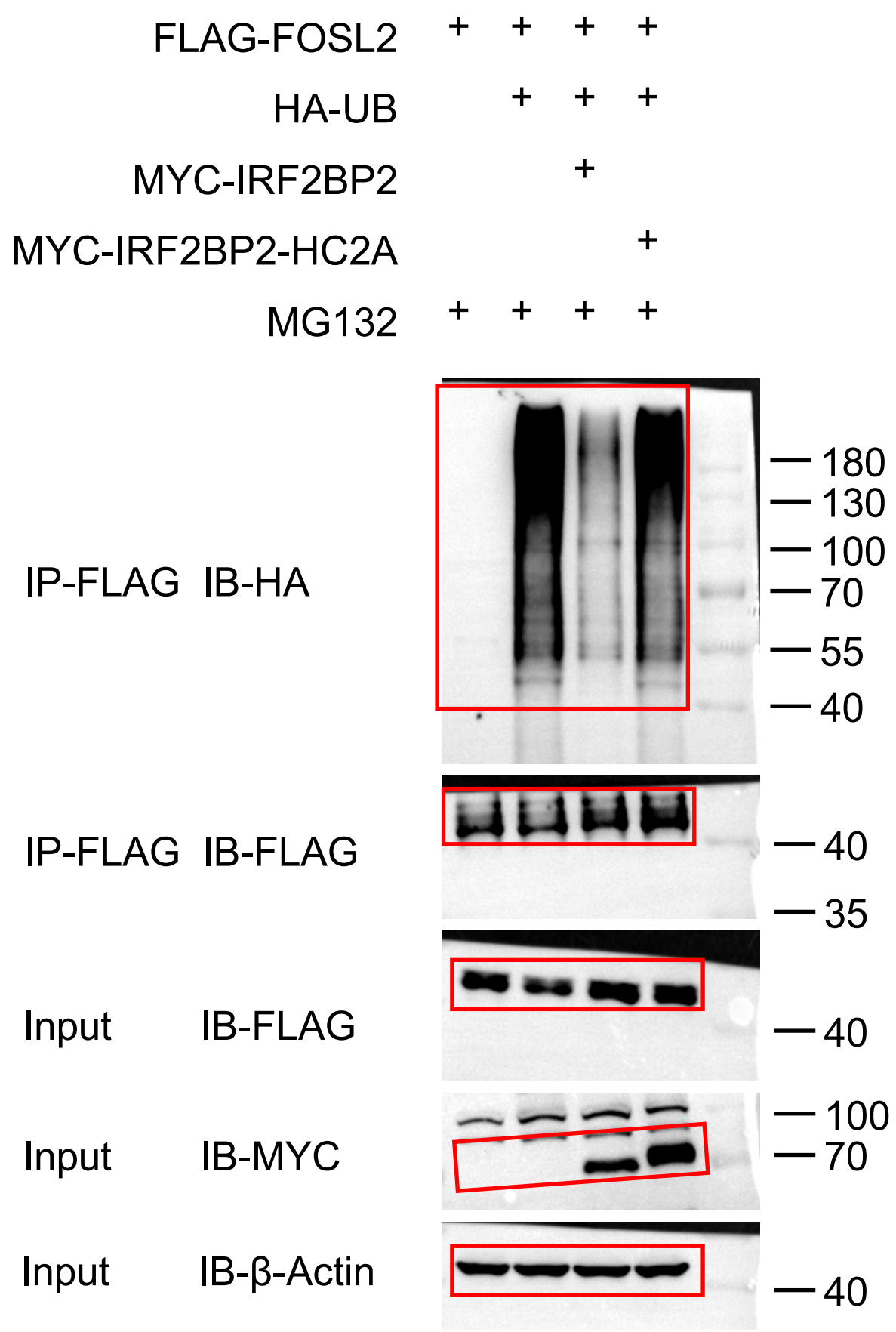

Full unedited blot for Figure S7A

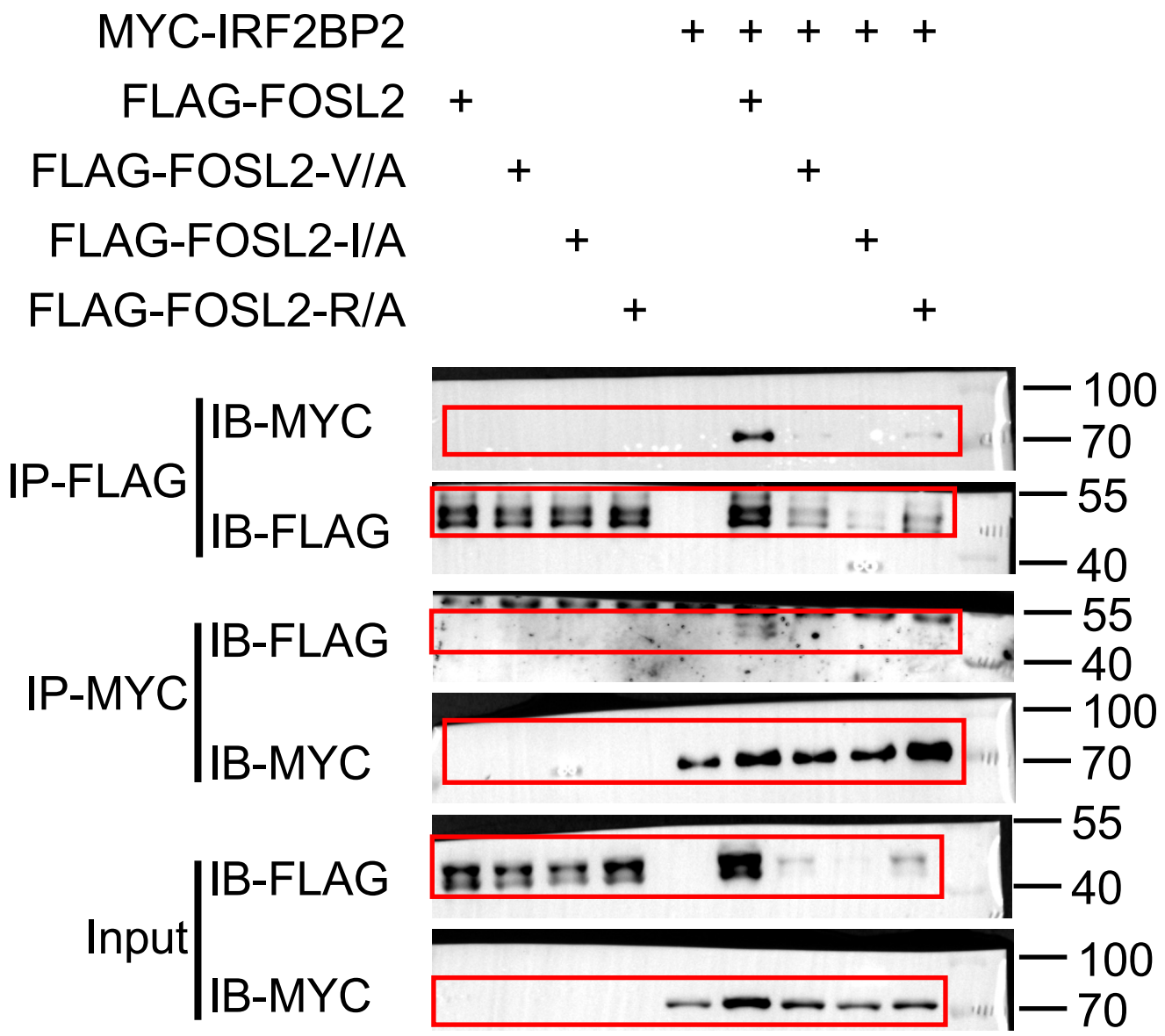

Full unedited blot for Figure S7B

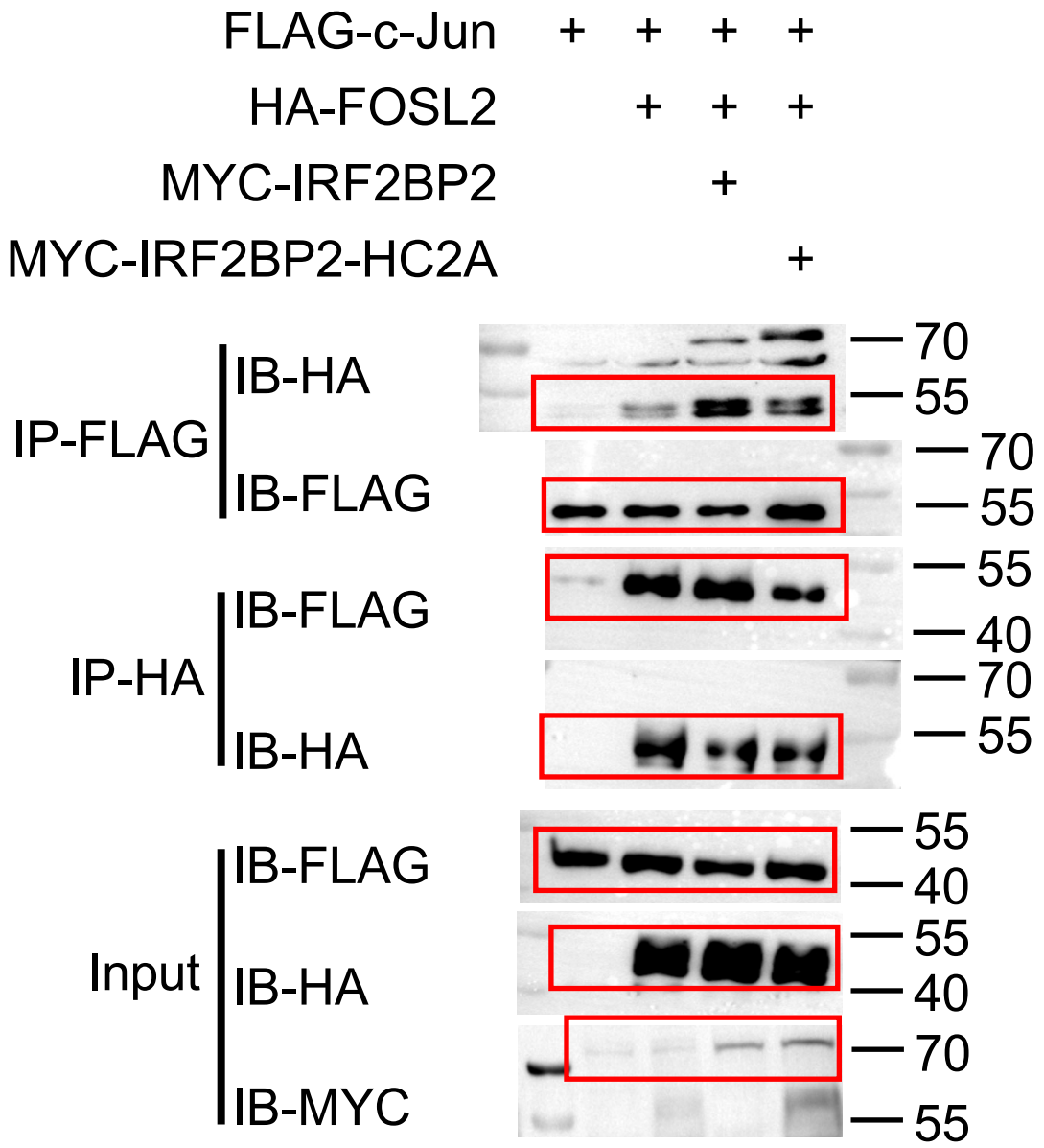

Full unedited blot for Figure S7C

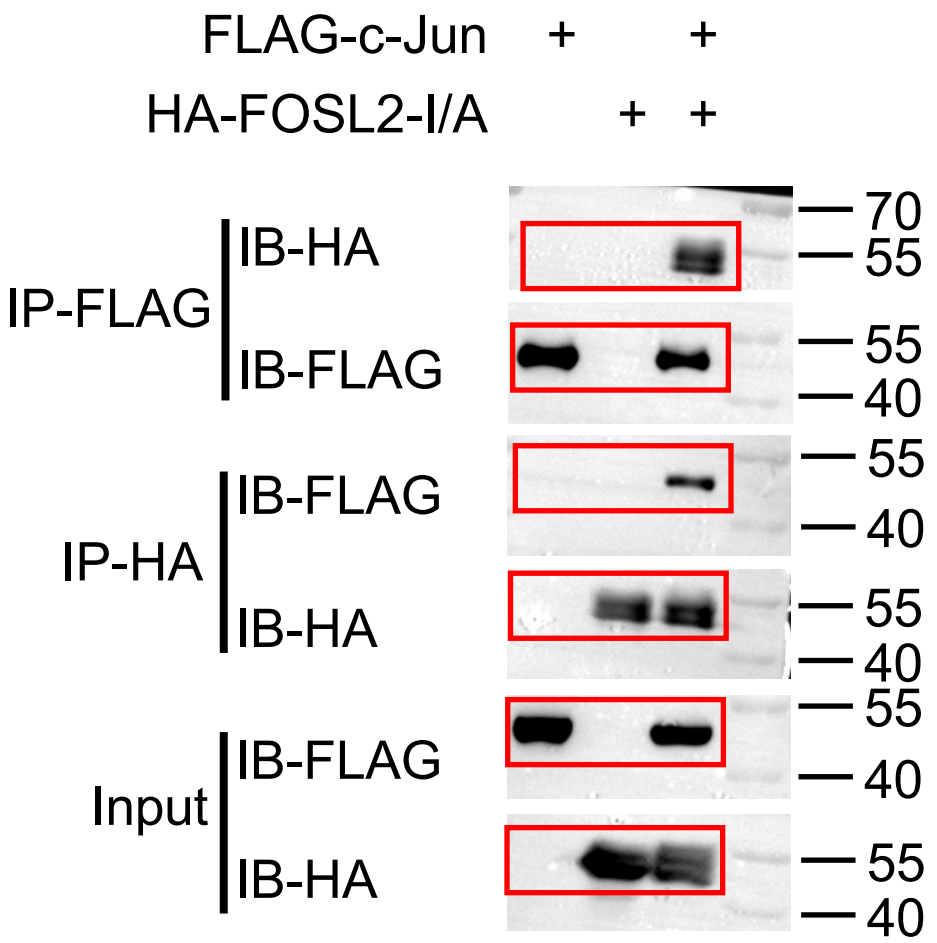

Full unedited blot for Figure S7D

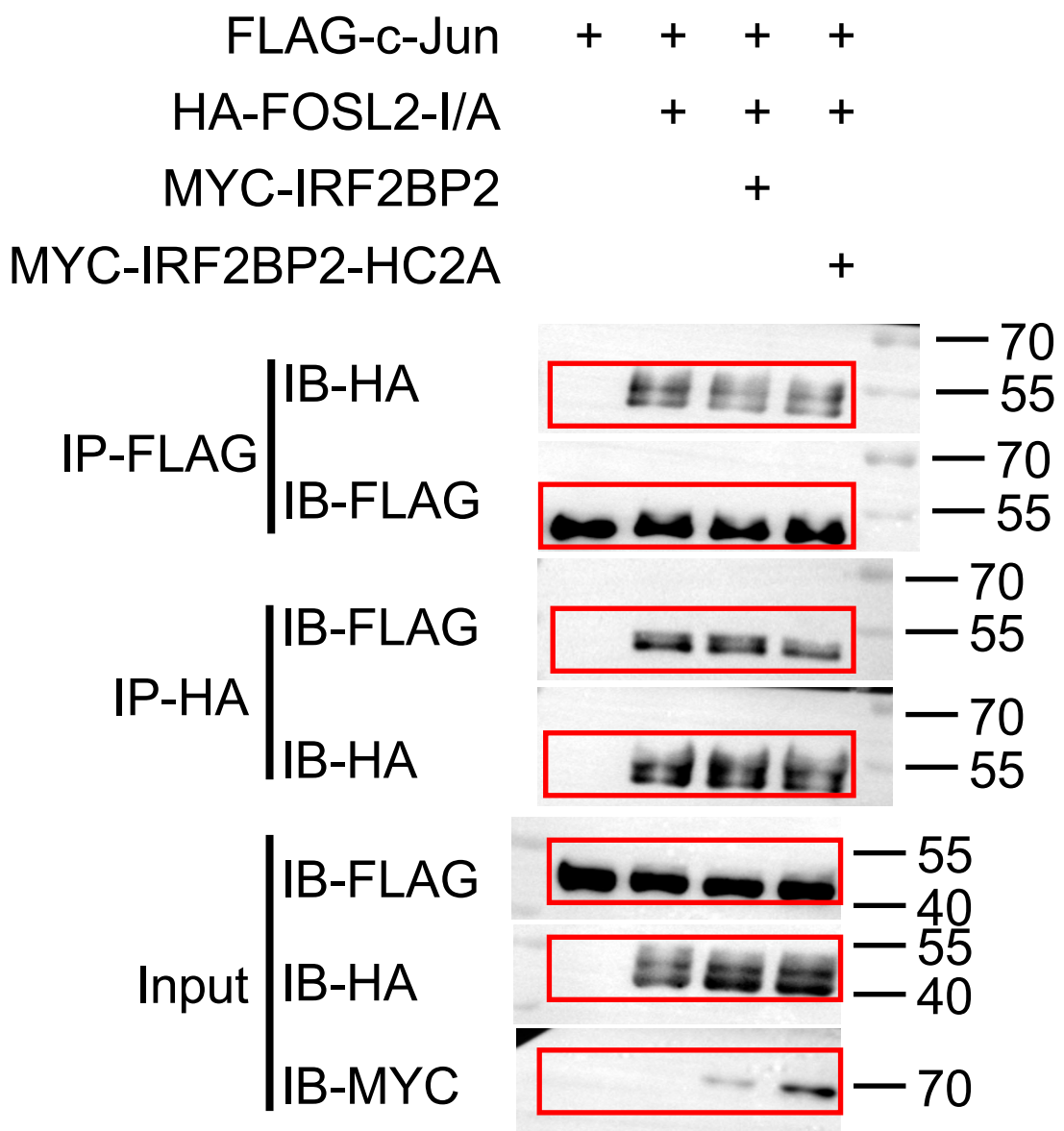

Full unedited blot for Figure S7E

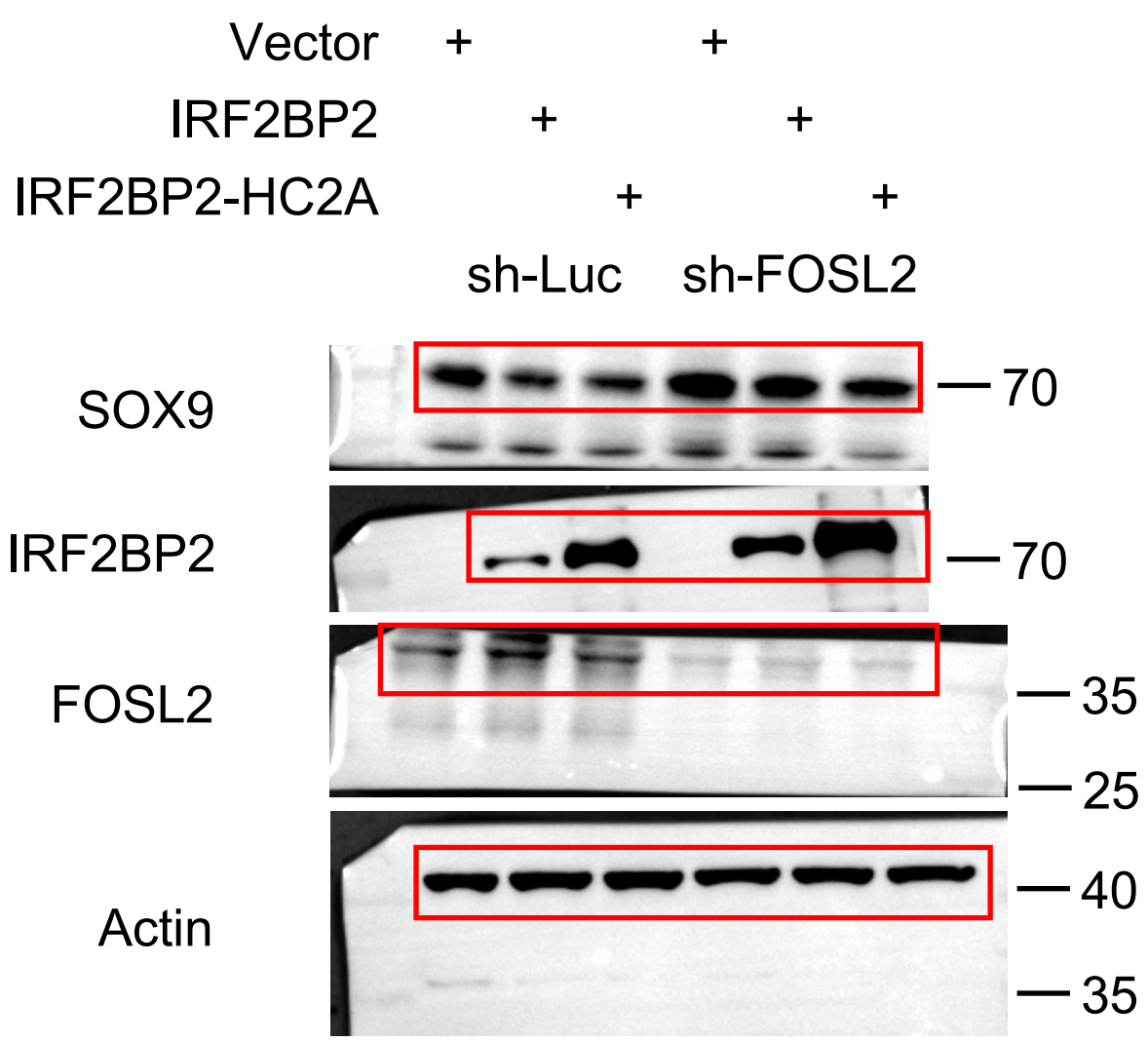

Full unedited blot for Figure S7F

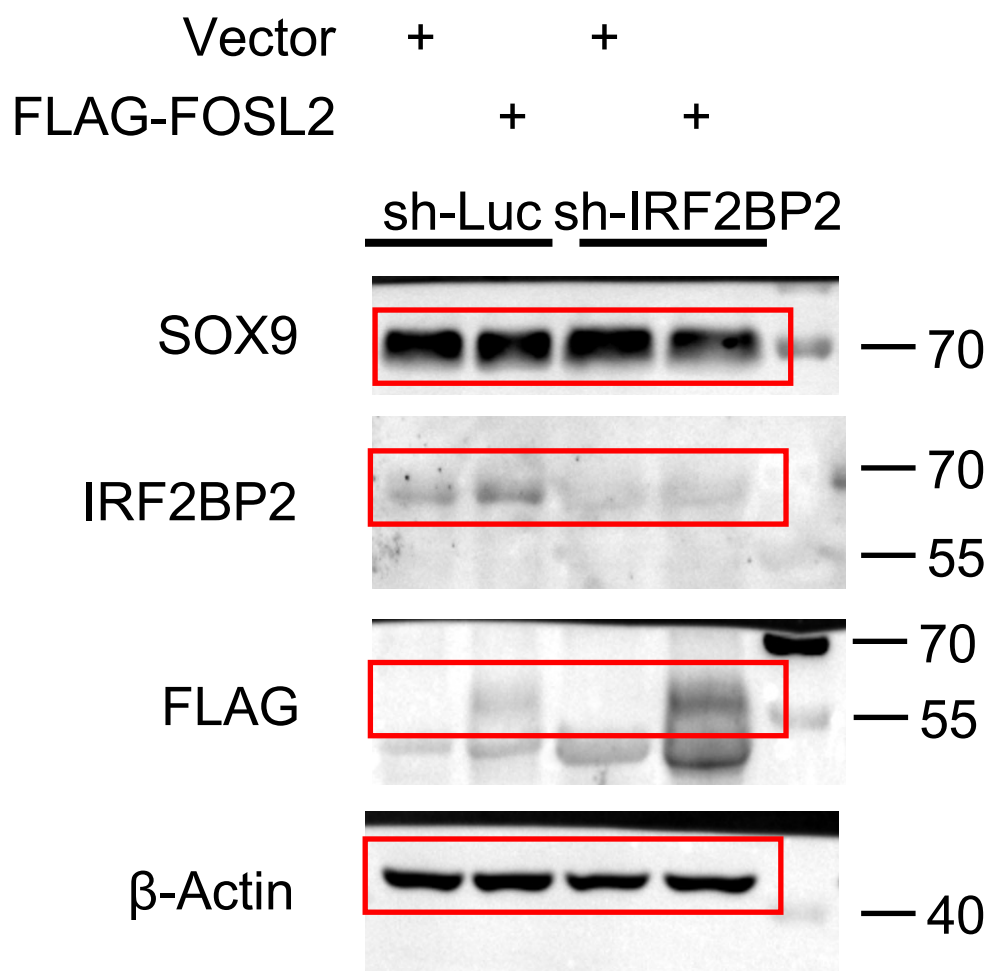

Full unedited blot for Figure S7G

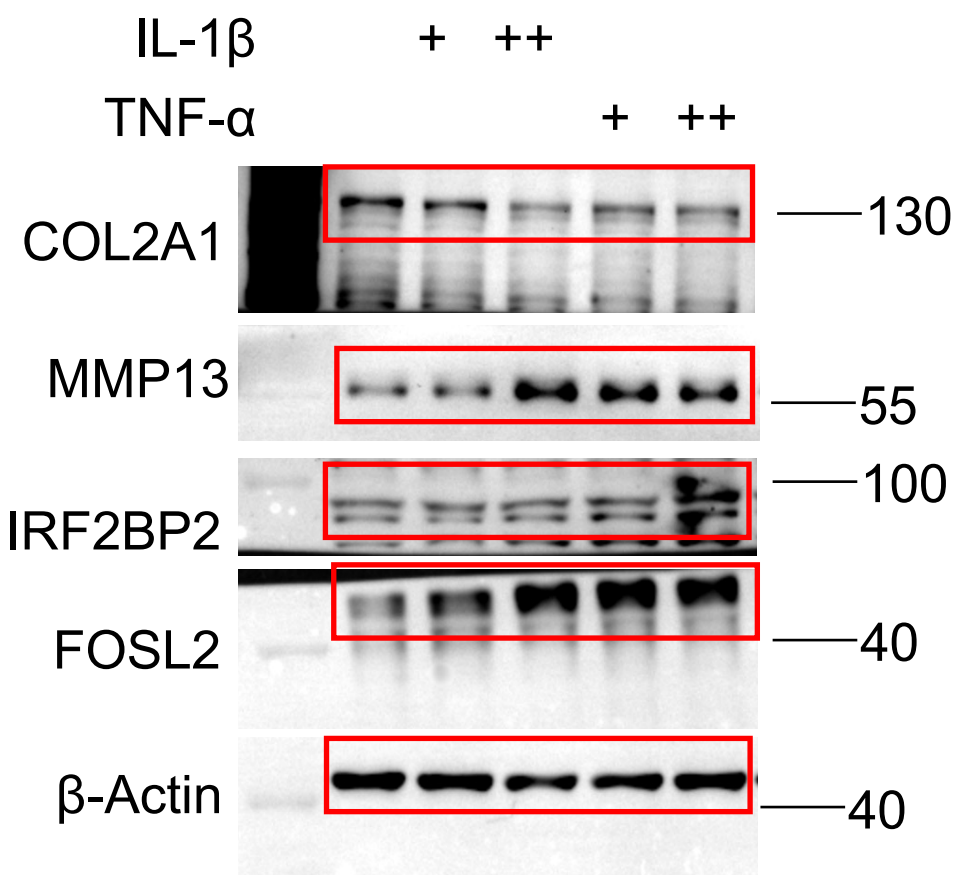

Full unedited blot for Figure S10A

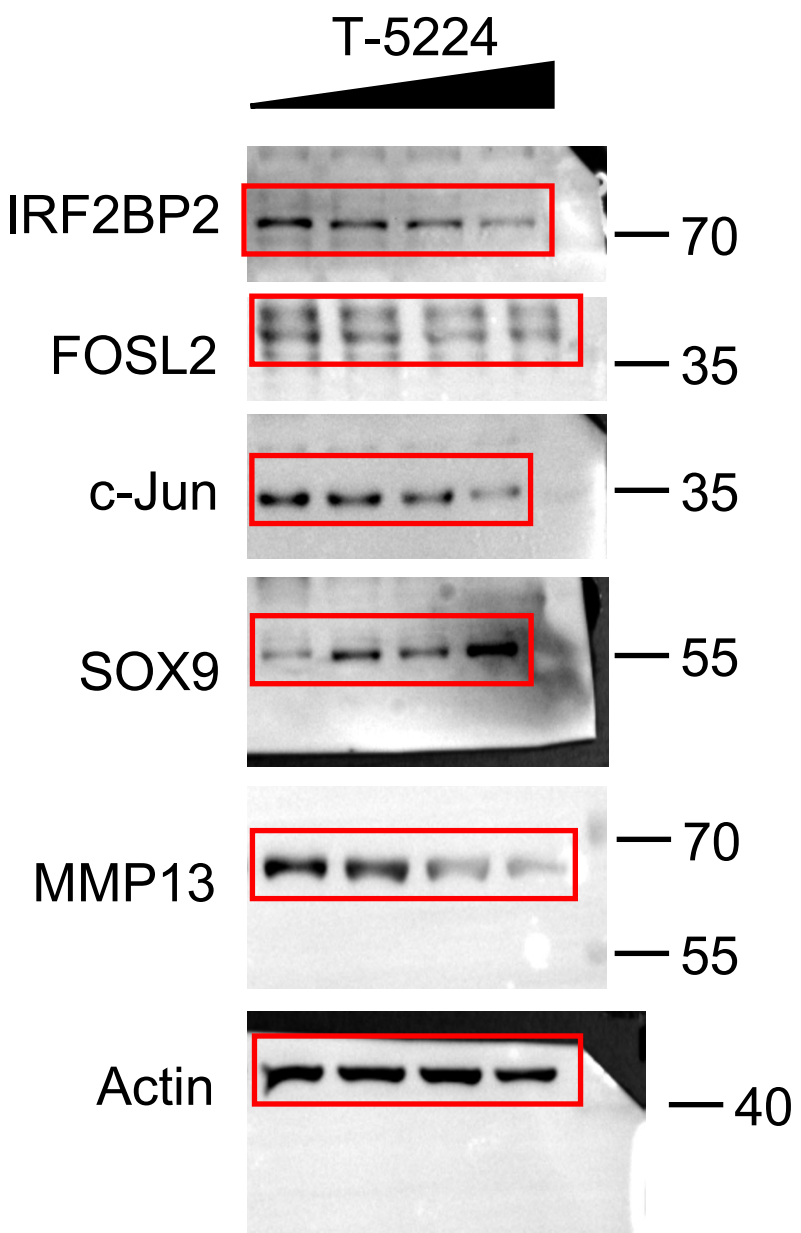

Full unedited blot for Figure S10B

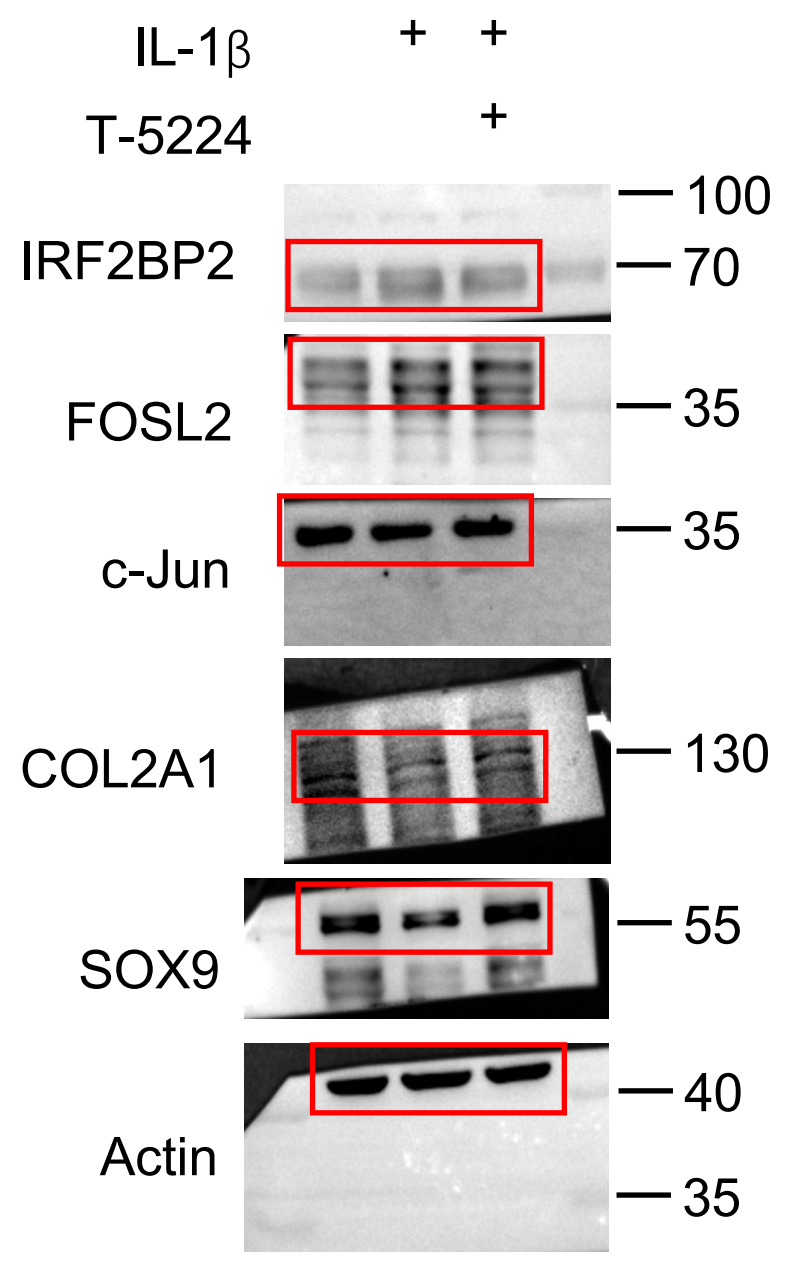

Full unedited blot for Figure S10D

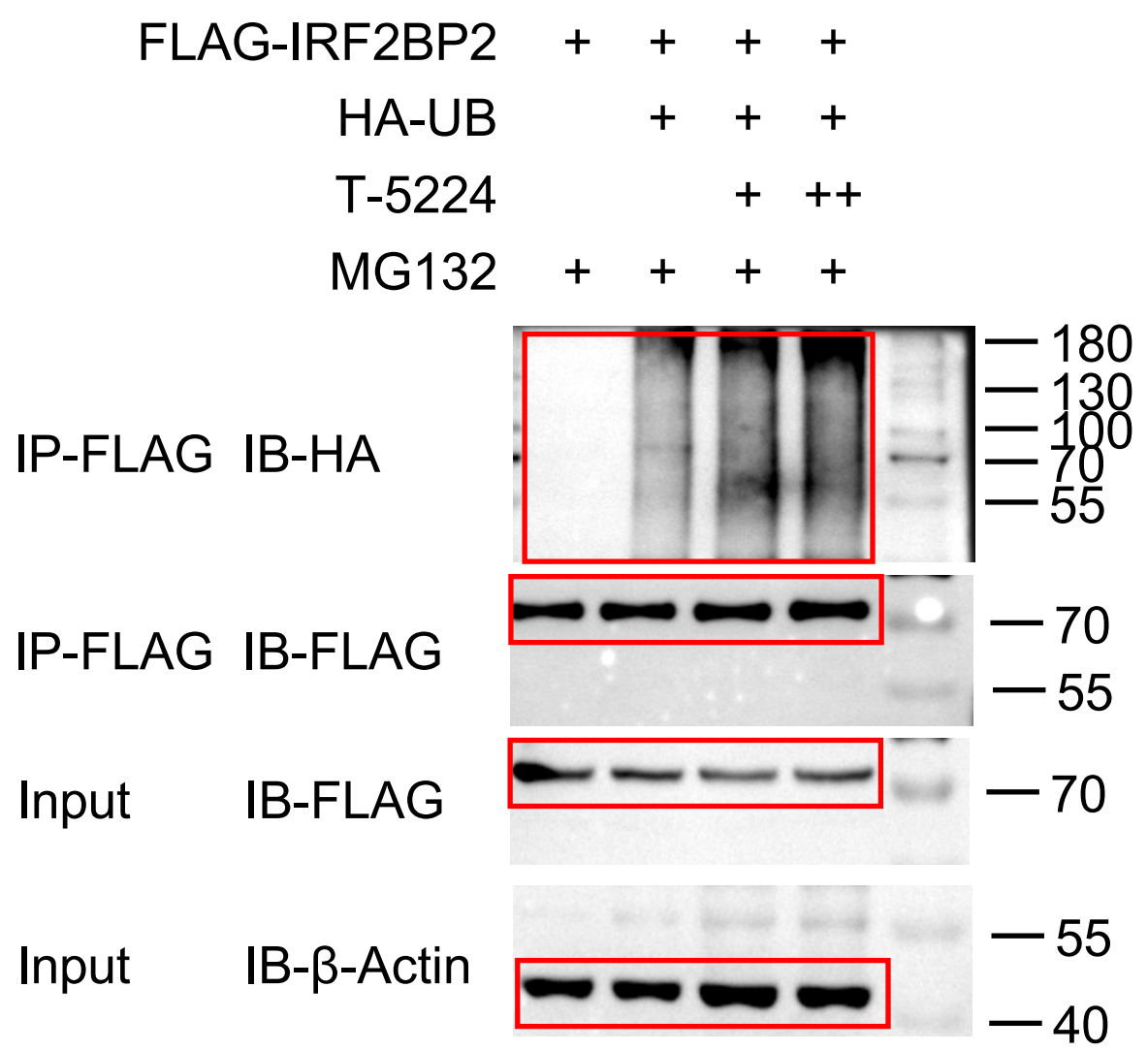

Full unedited blot for Figure S11A

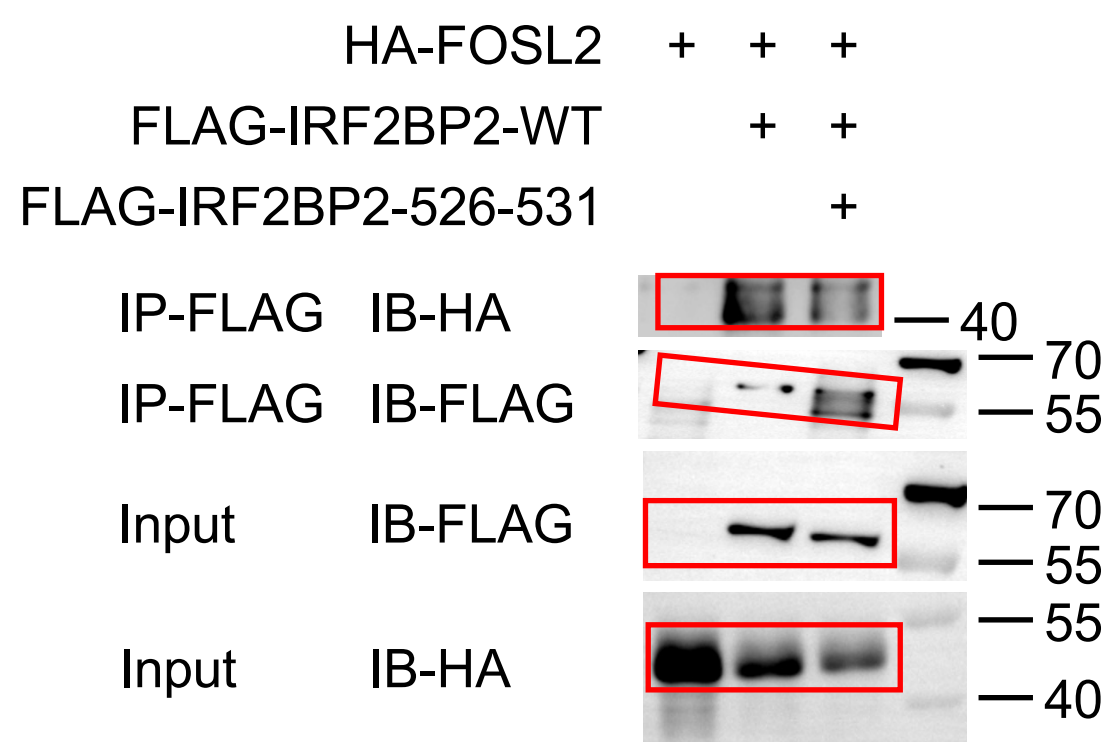

Full unedited blot for Figure S11B

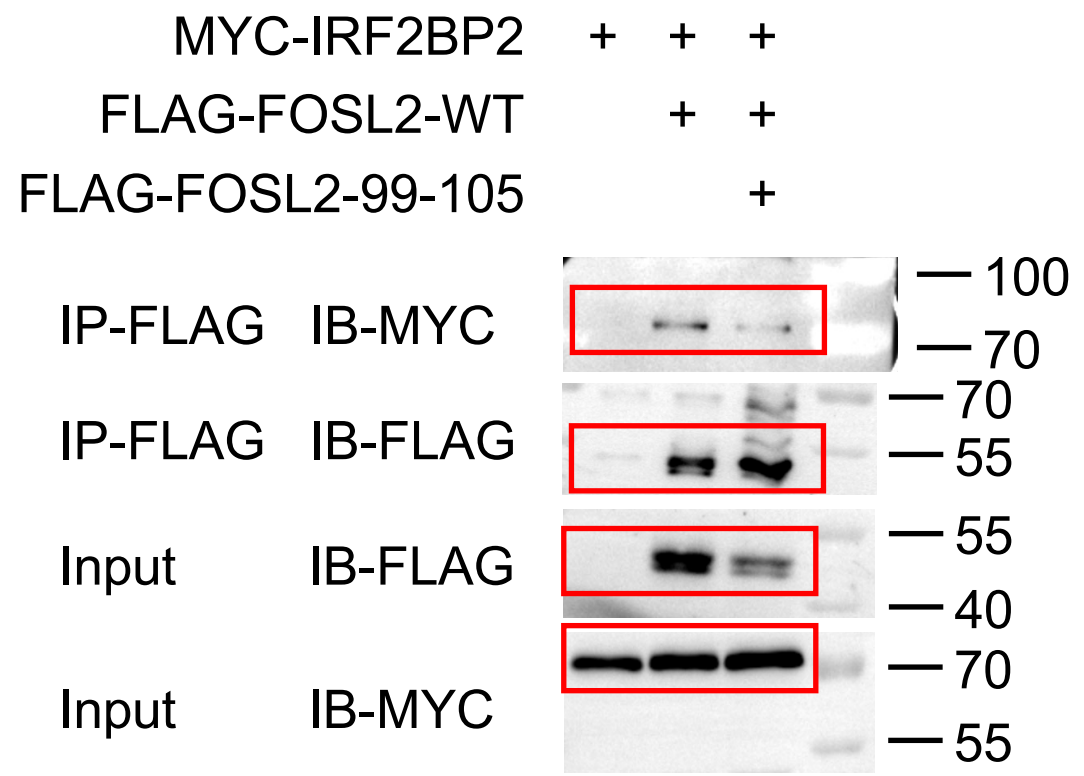

Full unedited blot for Figure S11C

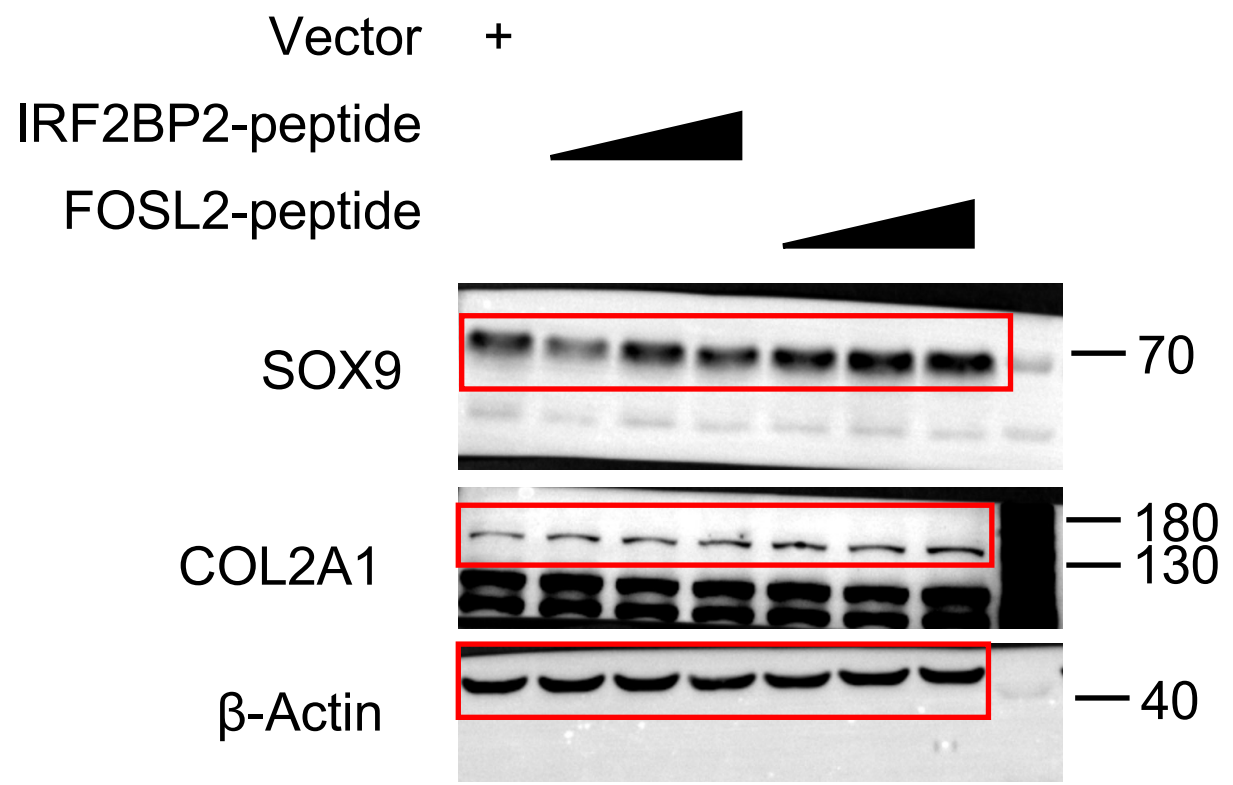

Full unedited blot for Figure S12A

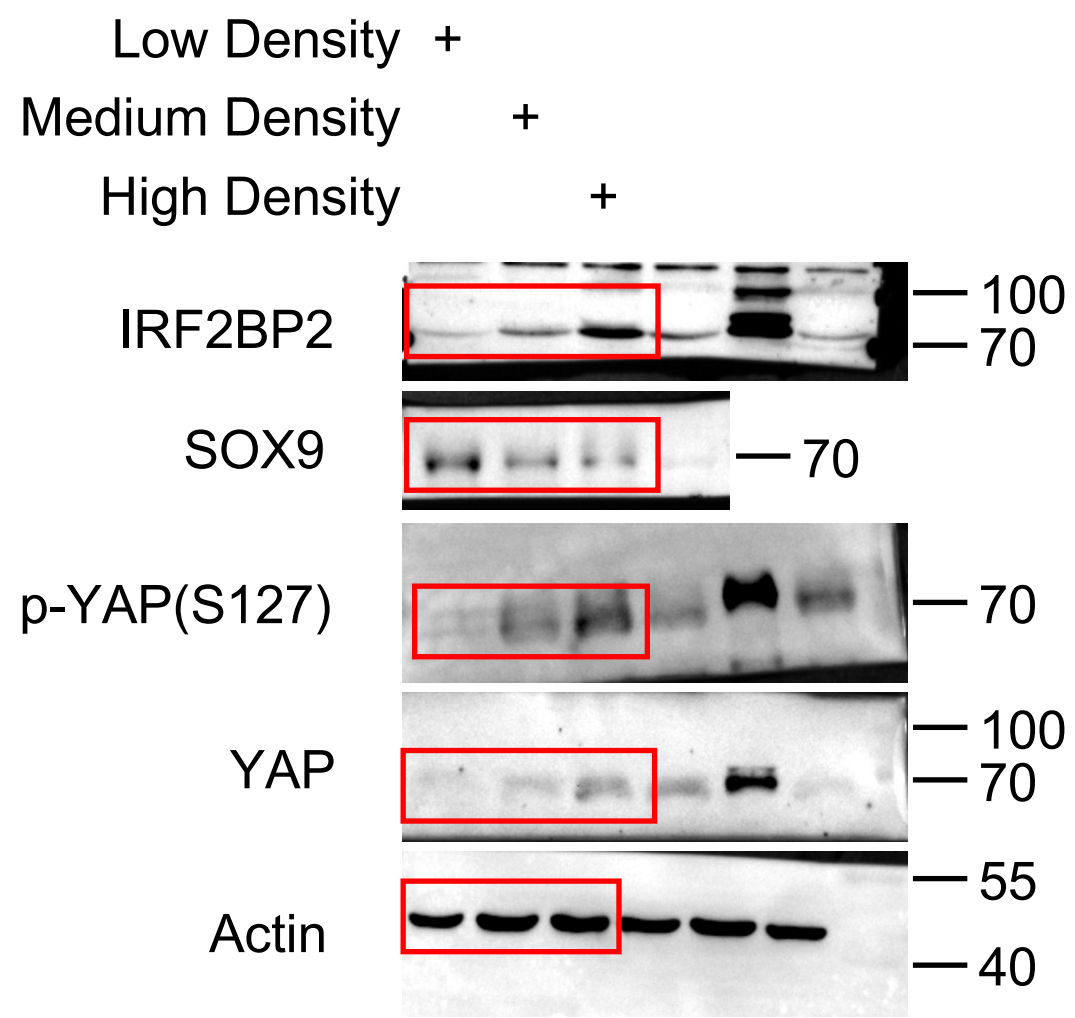

Supplement: Supplementary file 2 [file jci-136-200039-s065.pdf]
